# Evaluation of intestinal tissue safety during the compression process of circular end-to-end anastomosis stapler based on finite element simulation

**DOI:** 10.3389/fbioe.2025.1594969

**Published:** 2025-06-26

**Authors:** Yuanda Zhou, Zhen Tan, Peishi Jiang, Yi Sun, Dewang Wu

**Affiliations:** ^1^ Department of Colorectal Surgery, Tianjin Union Medical Center, The First Affiliated Hospital of Nankai University, Tianjin, China; ^2^ Nankai University School of Medicine, Nankai University, Tianjin, China; ^3^ The Institute of Translational Medicine, Tianjin Union Medical Center of Nankai University, Tianjin, China; ^4^ Tianjin Institute of Coloproctology, Tianjin, China; ^5^ Department of Anorectal Surgery, Gansu Provincial Hospital, Gansu Clinical Medical Research Center for Anorectal Diseases, Lanzhou, Gansu, China

**Keywords:** circular end-to-end anastomosis stapler, compression injury, finite element analysis, compression ratio, equivalent stress

## Abstract

**Objective:**

Currently, there is no standardized finite element analysis method for investigating the safe compression range of circular end-to-end anastomosis stapler. This study aims to develop a finite element analysis framework based on stress thresholds and the volumetric distribution of tissue states, and to investigate the effects of tissue thickness and compression ratio on the risk of compression-induced injury during anastomosis. The evaluation is conducted by calculating the proportion of the volume of elements categorized as “effective fixation” or “damaged” based on equivalent stress.

**Methods:**

A disposable circular end-to-end anastomosis stapler was used as the reference model to create a 1:1 scale 3D model of the key components at the contact surface, including the anvil, staple cartridge, and cutting washer. Finite element models of intestinal tissue with varying thicknesses were established within the environment of the circular stapler. Different compression ratios were applied to analyze the stress distribution in the intestinal tissue.

**Results:**

Across experiments with intestinal tissues of all thicknesses, the safe compression ratio consistently centered around 60%. The maximum equivalent stress on the lower intestinal segment was always greater than that on the upper segment, while the average equivalent stress of the upper and lower intestinal segments exhibited a collinear distribution across experiments with varying tissue thickness. An increase in total tissue thickness positively contributed to the expansion of the safe compression range. In asymmetric tissue thickness models, the side with greater thickness demonstrated a broader safe compression range.

**Conclusion:**

The safe compression range of staplers is closely related to the properties and thickness of the tissue. This study provides a framework for simulating and determining the safe compression range of staplers.

## 1 Introduction

In recent years, with continuous advancements in stapler technology, staplers have gradually replaced traditional manual suturing in colorectal surgeries (Lu Z. et al., 2016; Kajmolli et al., 2020). Circular end-to-end anastomosis stapler are primarily used in colorectal surgery, gastrointestinal surgery, bariatric surgery, and other procedures requiring circular anastomosis. The performance of circular end-to-end anastomosis stapler is influenced by multiple factors, including the compression area (Allen et al., 2018; Llorach-Perucho et al., 2024), compression distance (Son et al., 2020), compression speed (Kim et al., 2022), and the arrangement of the staples (Nakanishi et al., 2022). Therefore, evaluating the mechanical changes in tissues caused by staplers is of great significance for assessing the performance of anastomosis achieved by staplers (Myers et al., 2011). To address this, the authors designed a finite element analysis study to quantitatively evaluate the mechanical stress changes in intestinal wall tissues induced by circular end-to-end anastomosis stapler. The study analyzed the relationship between intestinal tissues of varying wall thicknesses and the optimal compression distance and compression ratio, aiming to identify a safe range of compression distance and ratio that prevents tissue injury.

Finite element method divides a continuum into discrete elements and uses shape functions within each element to approximate field variables, enabling efficient computation of stress and displacement distributions in complex geometries and multi-material systems (Liu et al., 2022). Consequently, finite element analysis has become a key method in medical device design and safety assessment, and is widely employed in the optimization of anastomotic staplers. Nováček et al. employed a finite-element model to evaluate the impact of varying staple heights on tissue stress and strain during colorectal end-to-end anastomosis; they found that increasing the outer-tier staple height significantly reduces tissue stress and strain compared with uniform-height staples across all three tiers (Nováček et al., 2012). Amano et al. demonstrated via finite-element analysis that novel filleted-corner Mg–2.5Nd–1Y alloy staples effectively mitigate stress and strain concentrations during both U to B forming and B shaped sealing processes, significantly reducing fracture risk (Amano et al., 2019).

Recent finite element analyses in biological tissues have predominantly employed equivalent stress measures, such as von Mises stress, along with direction-specific displacements as the primary evaluation metrics (Jiang et al., 2020; Mousavi et al., 2023; Teixeira and Martins, 2023). Although localized high stress can lead to tissue damage, biological tissues possess compensatory and self-repair mechanisms, such as cell necrosis and stem cell proliferation, to mitigate these effects. Staplers must not only minimize tissue injury but also ensure stable tissue positioning during deployment to prevent anastomotic failure. Given the challenges of conducting large-scale animal experiments for performance validation, we propose a finite element analysis approach based on tissue effective fixation and damage thresholds. This method offers a novel reference framework for optimizing stapler design, evaluating safety, and guiding clinical applications.

## 2 Materials and methods

### 2.1 Establishment of stapler and intestinal models

**FIGURE 1:**
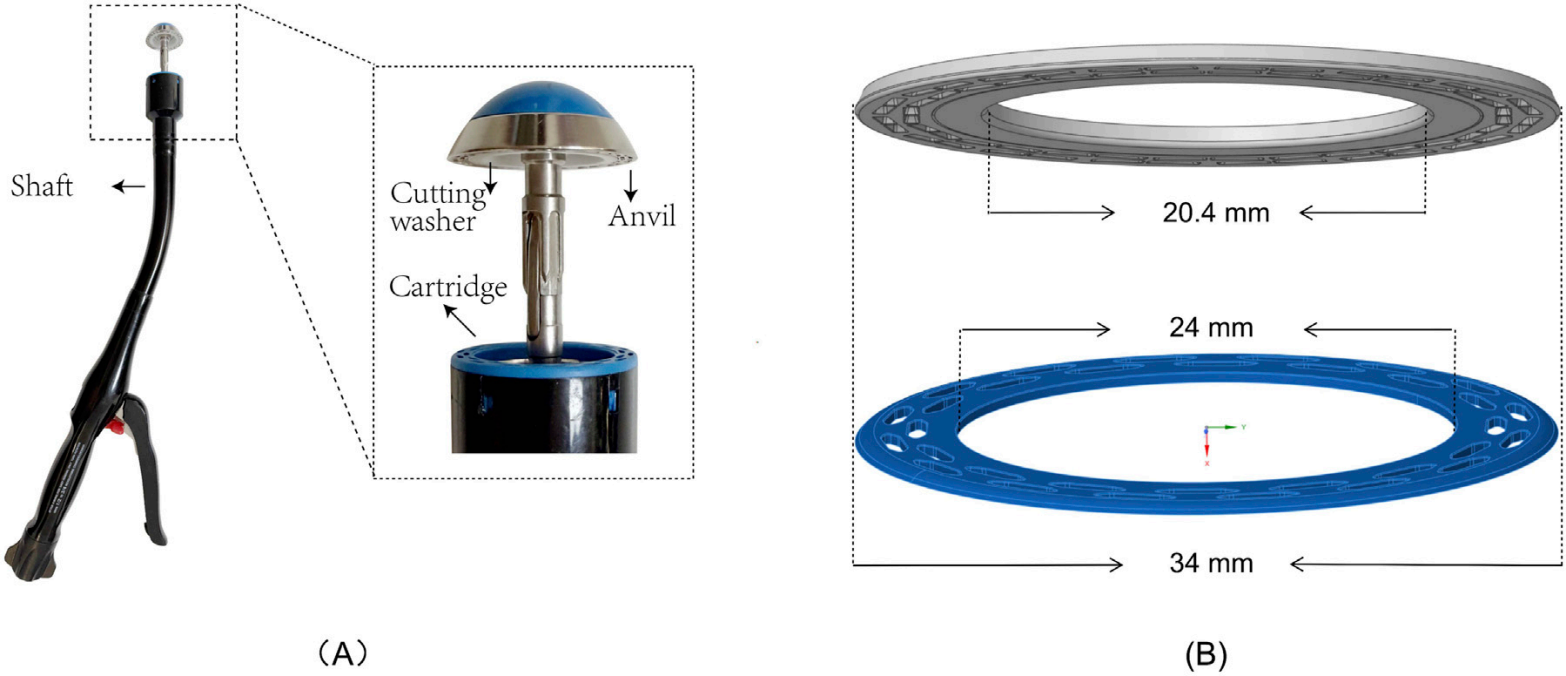
Schematic Diagram and 1:1 Modeling of Circular Stapler and Intestinal Tissue Contact Structure **(A)** Schematic diagram of the stapler (Model PYGX-34). **(B)** 1:1 modeling of the intestinal tissue contact structure, including the anvil and cutting washer (gray) and the staple cartridge (blue).

The Circular End-to-End Anastomosis Stapler mainly consists of a handle, a shaft, a cartridge, a cutting washer, and an anvil (Figure 1A). In practice, the cartridge is introduced into the distal bowel through the shaft, while the anvil is placed in the proximal bowel. Next, the adjustment knob on the handle is turned to bring the cartridge and anvil closer, with the real-time gap visible through an indicator window, until both cut ends are adequately clamped. After confirming correct positioning, depressing the trigger fires the stapler. Its built-in cutting blade simultaneously removes excess tissue on both sides, while the staples secure the bowel ends, completing the end-to-end anastomosis. In this study, a disposable circular end-to-end anastomosis stapler (manufacturer: Prestars Star Medical Devices Co., Ltd., Changzhou; model: PYGX-34) was used as the reference model. Key components at the contact interface, including (1) the anvil, (2) the staple cartridge, and (3) the cutting washer, were modeled in three dimensions at a 1:1 scale using Rhino seven software. Additionally, intestinal tissue of varying thicknesses was modeled as annular disk structures to facilitate subsequent assembly and analysis. The schematic diagram of the stapler and the 3D models is shown in Figure 1B.

According to the literature, the thickness of the colonic wall ranges from 0.2 mm to 2.5 mm (Wiesner et al., 2002), the rectal wall from 1.6 mm to 2.6 mm (Nylund et al., 2012), and the ileal wall from 1 mm to 2 mm (Cronin et al., 2010). To simplify the model, intestinal tissue in this study was represented as a hollow ring-shaped structure with an annular cross-section. The intestinal tissue adjacent to the anvil was designated as the upper layer, while the tissue near the staple cartridge was designated as the lower layer. The following thickness combinations were applied: upper and lower layers of 2.5 mm–2.5 mm, 2.0 mm–2.0 mm, 1.5 mm–1.5 mm, 2.5 mm–1.5 mm, and 1.5 mm–2.5 mm, respectively.

### 2.2 Material assignment

Based on prior *in vitro* experimental studies on porcine colonic tissue (Tran et al., 2011; Liao-yuan et al., 2018), in which tensile and compressive test data were fitted to an incompressible third-order Ogden hyperelastic constitutive model, the material parameters of the intestinal wall were modeled using constants μ_1_ = 8300 Pa, A_1_ = 7.625; μ_2_ = 200 Pa, A_2_ = 13.875; μ_3_ = 6200 Pa, A_3_ = 7.625. The finite element simulation analysis was performed using ANSYS 2023R1. To simplify computations, the anvil, cutting washer, and staple cartridge were treated as rigid bodies, neglecting deformation, whereas the intestinal tissue was modeled as a deformable body to capture its mechanical behavior.

### 2.3 Contact property settings

Three contact pairs were established: I. Bonded contact between the target geometry (anvil/cutting washer) and the contact geometry (upper layer tissue). II. Bonded contact between the target geometry (staple cartridge) and the contact geometry (lower layer tissue). III. Bonded contact between the target geometry (upper layer tissue) and the contact geometry (lower layer tissue).

### 2.4 Boundary condition settings

A remote displacement constraint was applied to the staple cartridge, with all degrees of freedom fixed (X, Y, Z translations and X, Y, Z rotations set to 0). A remote displacement constraint was also applied to the outer surface of the intestinal tissue, fixing Y, Z translations and X, Y, Z rotations while allowing freedom in the X direction (the X-axis is parallel to the direction of anastomosis compression). A displacement load was applied to the upper surface of both the anvil and the cutting washeralong the X-axis, which is the direction of tissue compression, with the number of steps set as the compression distance (mm) divided by 0.1 mm. The intestinal tissue was compressed by 0.1 mm per step, and the end time for each step was set to 1 s. The automatic time stepping was configured to program control. For the displacement load applied along the X-axis to the upper surfaces of both the anvil and cutting washer, the total movement was set to 0.8 × (upper tissue layer thickness + lower tissue layer thickness). Other settings in the Analysis Settings are based on the program’s default automatic control nonlinear finite element analysis method. Detailed settings are provided in Supplementary Table 1.

### 2.5 Mesh convergence study

The Ansys automatic meshing tool was used to generate meshes for the stapler components and intestinal tissue models, with mesh sizes set to 2 mm, 1.6 mm, 1.2 mm, 0.8 mm and 0.4 mm. A stepwise remote displacement load was applied to the anvil of the symmetric intestinal model with 2.5 mm–2.5 mm wall thickness until the stapler gap reached 1 mm. During this process, the variations in equivalent stress were monitored. When the variation in equivalent stress between two consecutive mesh sizes was less than 3% of the preceding value, mesh convergence was considered.

### 2.6 Mesh generation

The stapler and intestinal tissue models were meshed using Ansys automatic meshing tools with a uniform mesh applied to all components.

### 2.7 Model validation

The model was validated by applying a stepwise remote displacement load to the anvil of the 2 mm symmetrical intestinal model until the stapler gap reached 1 mm. The results were compared with the *in vitro* collagen block compression experiments (Son et al., 2020). The force exerted on the intestinal tissue at different compression ratios was observed and analyzed.

### 2.8 Study on Stress Distribution in Intestinal Tissue

According to the literature, the safe pressure range for dry collagen is reported to be 12–50 g/mm^2^ (i.e., 0.12–0.5 MPa) (Kim et al., 2022). During gastrointestinal anastomosis in pigs, a pressure range of 30–60 N/cm^2^ (i.e., 0.3–0.6 MPa) is considered an ideal safe range when the stapler gap is 2 mm (Cope, 1995; Myers et al., 2010). Based on this data, this study assumes that equivalent stress within the range of 0.3–0.6 MPa ensures that tissue within the elements space does not sustain damage and achieves effective fixation. The following criterion was established for well-fixed tissue: the proportion of elements volume with equivalent stress in the range of 0.3–0.6 MPa exceeds 20% of the total volume of fixed tissue.

Considering factors such as non-convergence of maximum equivalent stress, potential abnormal stress concentration caused by stapler structural details, and the tissue’s natural healing capacity, the study established the following threshold for potential irreversible tissue damage: when the elements volume with equivalent stress exceeding 0.6 MPa accounts for more than 5% of the total volume of fixed tissue. Additionally, elements volumes with equivalent stress greater than 0.1 MPa were defined as the total volume of fixed tissue.

Displacement loads were applied to symmetrical intestinal models with thicknesses of 1.5 mm, 2.0 mm, and 2.5 mm, as well as asymmetrical models with thicknesses of 2.5 mm–1.5 mm and 1.5 mm–2.5 mm, until a compression ratio of 80% was reached. Changes in stress and its distribution were observed by varying the compression distance and ratio. The ANSYS Parametric Design Language (APDL) code for calculating the elements volume of stress ranges for different components is provided in Supplementary Data Sheet 1.

## 3 Results

### 3.1 Mesh convergence study

Mesh convergence in Fig. A showed only a 1.5% change in mean equivalent stress when refining from 0.8 mm to 0.4 mm, below our 3% convergence threshold (Figure 3A). Therefore, we adopted a 0.8 mm mesh.

### 3.2 Mesh generation results

**FIGURE 2:**
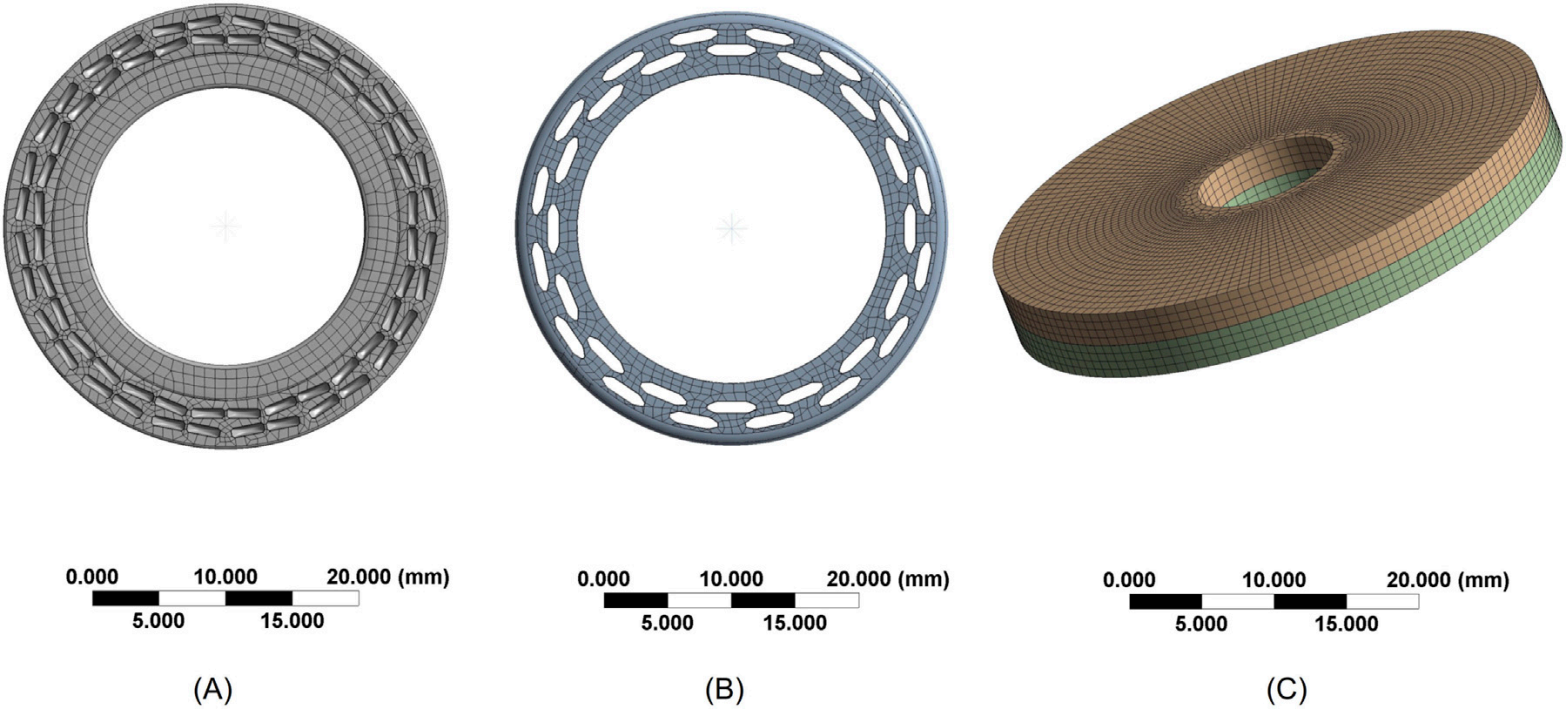
Mesh Generation Diagrams **(A)** Mesh of the contact surface between the anvil and cutting washer **(B)** Mesh of the staple cartridge contact surface **(C)** Mesh of the intestinal tissue.

The automatic meshing produced a mixed-element mesh: solid regions were meshed predominantly with Hex8 (8-node linear hexahedral) elements; transition zones where hexahedral layout was infeasible used Wedge6 (6-node linear wedge) elements; and thin-walled features were modeled with TriShell3/QuadShell4 shell elements. The final numbers of nodes and elements are presented in Table 1. The meshing diagrams of each component after grid division are presented in Figure 2.

**TABLE 1:** Mesh generation information.

Component	Anvil/Cutting washer	Staple cartridge	Intestinal tissue 1.5 mm	Intestinal tissue 2 mm	Intestinal tissue 2.5 mm
Number of Nodes	2,698	1843	7,814	15,380	20,125
Number of Elements	2,380	1,589	5,088	11,241	15,712

### 3.3 Model comparison

**FIGURE 3:**
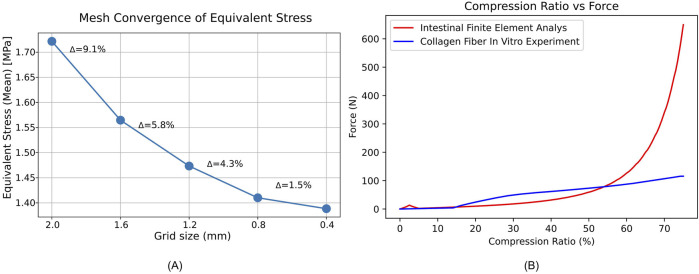
Mesh convergence and force compression behavior of intestinal tissue. **(A)** Mean equivalent stress (MPa) versus mesh size (mm); Δ shows the percent change from the previous point. **(B)** Force-Compression Ratio Trend Graph for Intestinal Tissue with 2 mm–2 mm Thickness.

In the Force-Compression Ratio trend graph for 2 mm–2 mm intestinal tissue, the results of this study were compared with previous *in vitro* experimental findings (Son et al., 2020). Our study exhibited strong concordance with previous research when the compression ratio was below 60%. Between 60% and 75%, the pressure predicted by the hyperelastic finite element model was significantly higher than that observed in the collagen fiber *in vitro* experiments under the stapler, yet it remained within the same order of magnitude. This suggests that the developed finite element model demonstrates reasonable validity and has the potential for further analysis (Figure 3B).

### 3.4 Maximum and average equivalent stress

**FIGURE 4:**
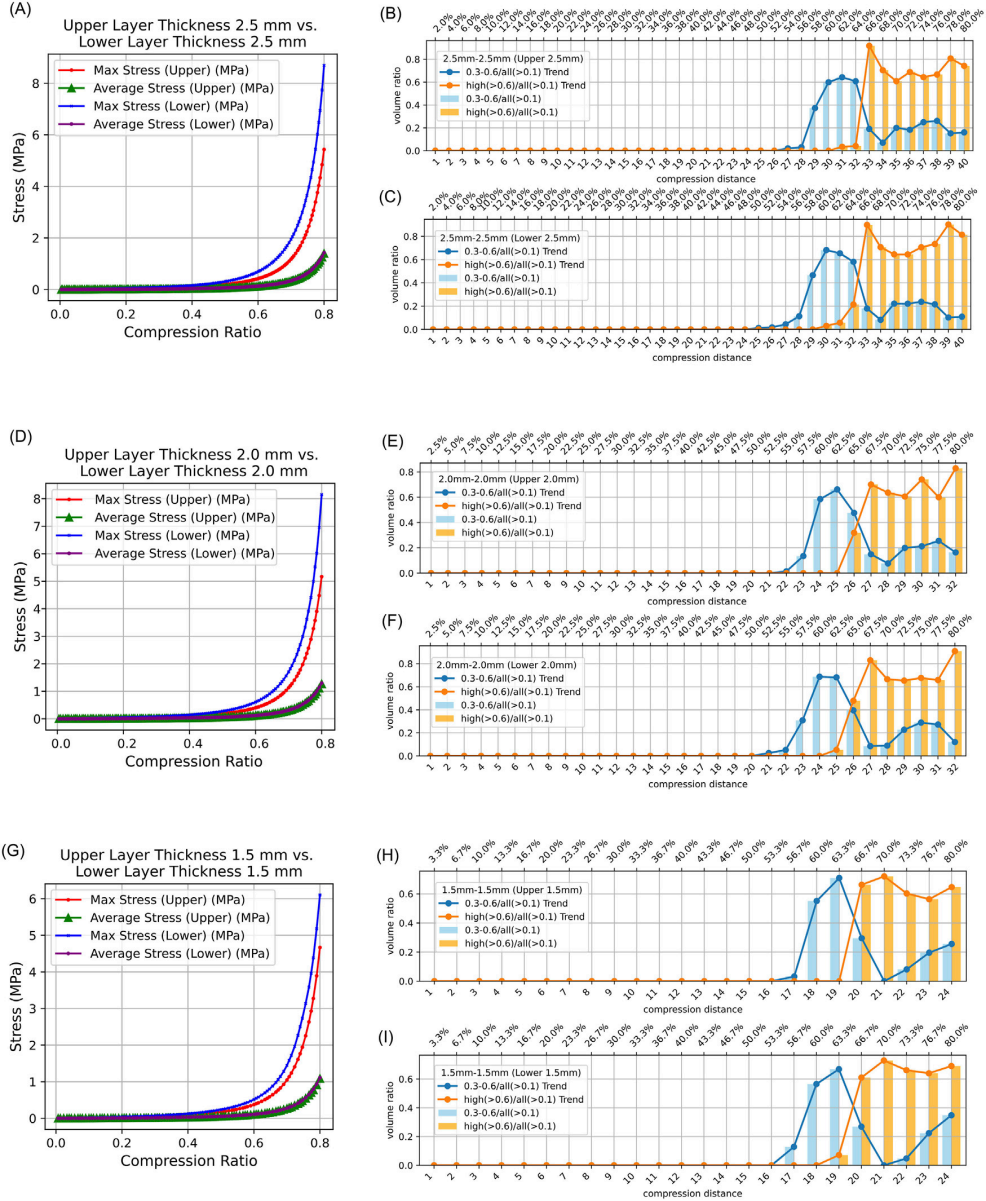
Stress Distribution in Intestinal Tissue with Symmetrical Thickness. (0.3–0.6/all (>0.1): The proportion of tissue volume where equivalent stress falls within 0.3 MPa–0.6 MPa, relative to the total tissue volume where stress exceeds 0.1 MPa; high (>0.6)/all (>0.1): The proportion of tissue volume where equivalent stress is greater than 0.6 MPa, relative to the total tissue volume where stress exceeds 0.1 MPa). **(A)** Average and maximum equivalent stress in the upper layer and lower layer of 2.5 mm–2.5 mm intestinal tissue under different compression ratios. **(B)** Proportion of elements volumes with equivalent stress between 0.3 MPa and 0.6 MPa and above 0.6 MPa in the upper intestinal tissue relative to all elements volumes exceeding 0.1 MPa under different compression ratios. **(C)** Proportion of elements volumes with equivalent stress between 0.3 MPa and 0.6 MPa and above 0.6 MPa in the lower intestinal tissue relative to all elements volumes exceeding 0.1 MPa under different compression ratios. **(D)** Average and maximum equivalent stress in the upper layer and lower layer of 2.0 mm–2.0 mm intestinal tissue under different compression ratios. **(E)** Proportion of elements volumes with equivalent stress between 0.3 MPa and 0.6 MPa and above 0.6 MPa in the upper intestinal tissue of 2.0 mm–2.0 mm relative to all elements volumes exceeding 0.1 MPa under different compression ratios. **(F)** Proportion of elements volumes with equivalent stress between 0.3 MPa and 0.6 MPa and above 0.6 MPa in the lower intestinal tissue of 2.0 mm–2.0 mm relative to all elements volumes exceeding 0.1 MPa under different compression ratios. **(G)** Average and maximum equivalent stress in the upper layer and lower layer of 1.5 mm–1.5 mm intestinal tissue under different compression ratios. **(H)** Proportion of elements volumes with equivalent stress between 0.3 MPa and 0.6 MPa and above 0.6 MPa in the upper intestinal tissue of 1.5 mm–1.5 mm relative to all elements volumes exceeding 0.1 MPa under different compression ratios. **(I)** Proportion of elements volumes with equivalent stress between 0.3 MPa and 0.6 MPa and above 0.6 MPa in the lower intestinal tissue of 1.5 mm–1.5 mm relative to all elements volumes exceeding 0.1 MPa under different compression ratios.

**FIGURE 5:**
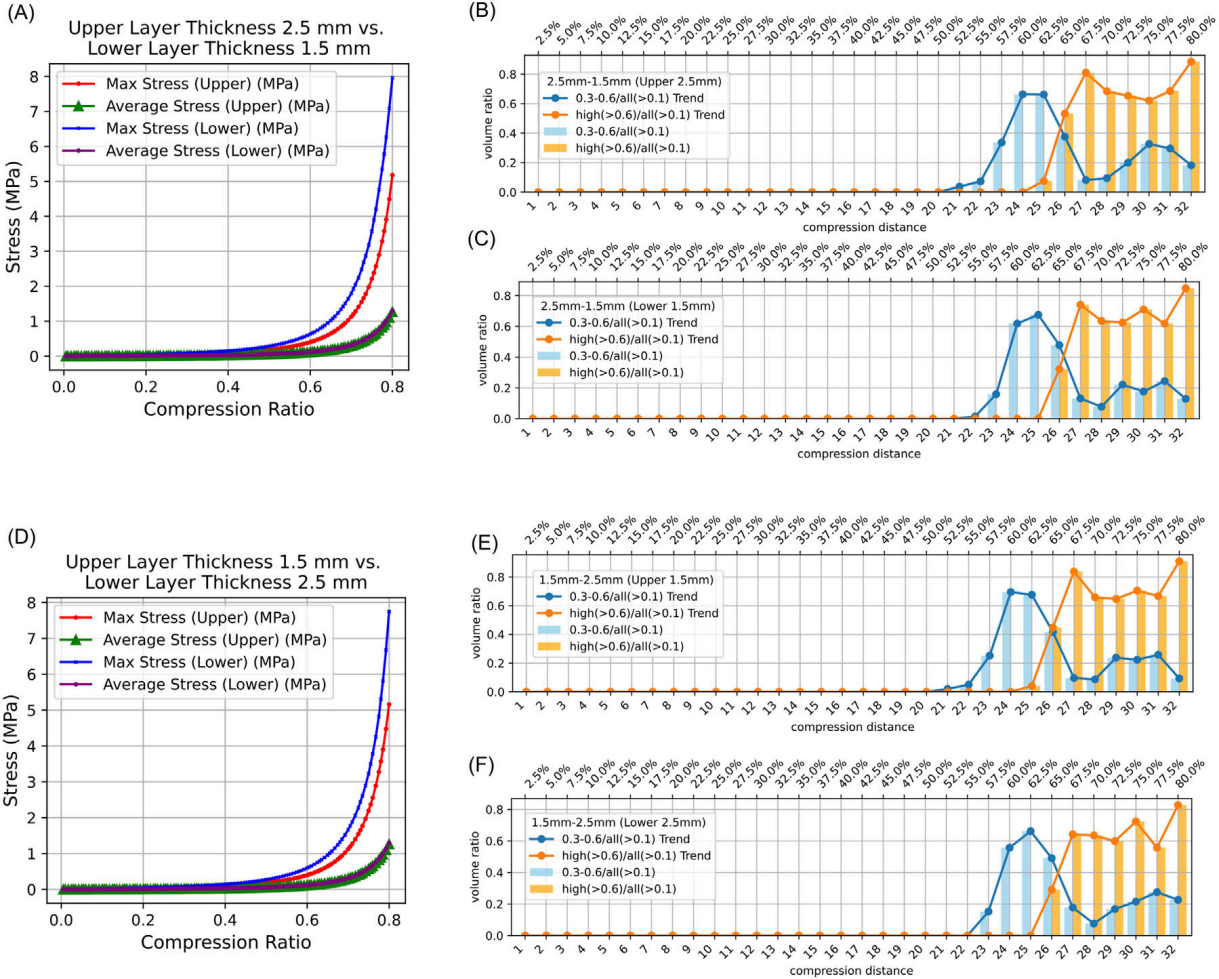
Stress Distribution in Intestinal Tissue with Asymmetrical Thickness. (0.3–0.6/all (>0.1): The proportion of tissue volume where equivalent stress falls within 0.3 MPa–0.6 MPa, relative to the total tissue volume where stress exceeds 0.1 MPa; high (>0.6)/all (>0.1): The proportion of tissue volume where equivalent stress is greater than 0.6 MPa, relative to the total tissue volume where stress exceeds 0.1 MPa). **(A)** Average and maximum equivalent stress in the upper and lower layers of 2.5 mm–1.5 mm intestinal tissue under different compression ratios. **(B)** Proportion of elements volumes with equivalent stress between 0.3 MPa and 0.6 MPa, and above 0.6 MPa, in the upper intestinal tissue relative to all elements volumes exceeding 0.1 MPa under different compression ratios. **(C)** Proportion of elements volumes with equivalent stress between 0.3 MPa and 0.6 MPa, and above 0.6 MPa, in the lower intestinal tissue relative to all elements volumes exceeding 0.1 MPa under different compression ratios. **(D)** Average and maximum equivalent stress in the upper and lower layers of 1.5 mm–2.5 mm intestinal tissue under different compression ratios. **(E)** Proportion of elements volumes with equivalent stress between 0.3 MPa and 0.6 MPa, and above 0.6 MPa, in the upper intestinal tissue of 2.0 mm–2.0 mm relative to all elements volumes exceeding 0.1 MPa under different compression ratios. **(F)** Proportion of elements volumes with equivalent stress between 0.3 MPa and 0.6 MPa, and above 0.6 MPa, in the lower intestinal tissue of 2.0 mm–2.0 mm relative to all elements volumes exceeding 0.1 MPa under different compression ratios.

In both symmetrical and asymmetrical thickness experiments on intestinal tissue, the maximum equivalent stress in the lower intestinal tissue was always greater than that in the upper intestinal tissue. Additionally, the average equivalent stress in the lower and upper intestinal tissues was consistently collinear (Figures 4A,D,G, 5A,D). Detailed data can be found in Supplementary Table 2.

### 3.5 Results of compression ratio, intestinal tissue thickness, and safety analysis

The results indicate that regardless of whether the intestinal tissue thickness is symmetrical or asymmetrical, the safe compression ratio remains approximately 60%. In experiments with symmetrical thickness, a larger total thickness generally results in a broader safe compression range. For example, when the total thickness is 3 mm, the safe compression distance for the upper tissue is only 1.8 mm and 1.9 mm. However, when the total thickness increases to 5 mm, the safe compression distance for the upper tissue expands to 2.9 mm, 3.0 mm, 3.1 mm, and 3.2 mm (Table 2). Moreover, in symmetrical thickness combinations such as 2.5 mm–2.5 mm and 1.5 mm–1.5 mm, the safe compression range for the upper tissue is observed to be greater than that of the lower tissue. In contrast, for asymmetrical thickness combinations (1.5 mm–2.5 mm and 1.5 mm–1.5 mm), when the thickness on one side increases, the safe compression range on that side significantly expands. This demonstrates that variations in thickness have a substantial impact on compression performance (Figures 4B,C,E,F,H,I, 5B,C,E,F). Detailed data can be found in Supplementary Table 3. The equivalent stress contour maps under different compression ratios are presented in Supplementary Video S1, and the equivalent strain contour maps are shown in Supplementary Video S2.

**TABLE 2:** Safe compression distance, ratio, and gap.

Tissue pair	Tissue layer	Safe compression distance (mm)	Safe compression ratio	Safe gap (mm)
1.5mm-1.5 mm	Upper (1.5 mm)	1.8, 1.9	0.60,0.63	1.2
	Lower (1.5 mm)	1.8	0.60	
1.5 mm–2.5 mm	Upper (1.5 mm)	2.4, 2.5	0.60,0.63	1.6,1.5
	Lower (2.5 mm)	2.3, 2.4, 2.5	0.58,0.60,0.63	
2.0 mm–2.0 mm	Upper (2.0 mm)	2.4, 2.5	0.60,0.63	1.6
	Lower (2.0 mm)	2.3, 2.4	0.58,0.60	
2.5 mm–1.5 mm	Upper (2.5 mm)	2.4, 2.5	0.60,0.63	1.6
	Lower (1.5 mm)	2.3, 2.4	0.58,0.60	
2.5 mm–2.5 mm	Upper (2.5 mm)	2.9, 3.0, 3.1, 3.2	0.58,0.60.0.62,0.64	2.0,2.1
	Lower (2.5 mm)	2.9, 3.0	0.58,0.60	

## 4 Discussion

Anastomotic leakage is one of the severe complications following rectal cancer resection. It not only leads to systemic infections and prolonged hospitalization but also promotes tumor recurrence and metastasis (Lu Z. R. et al., 2016; Wang et al., 2017). Therefore, early prevention of anastomotic leakage is critically important. Current clinical strategies to prevent anastomotic leakage include rectal suspension suturing techniques (Ge et al., 2024), prophylactic stoma creation (Zheng et al., 2023), and pre-positioned anal tube drainage (Kawada et al., 2018). When using circular end-to-end anastomosis stapler, excessive tissue deformation can result in tissue damage (Kim et al., 2022). Surgeons must consider staple height, tissue thickness, and tissue type comprehensively when selecting the compression distance to improve patient outcomes (Chekan and Whelan, 2014).

Finite element biomechanical analysis involves the application of the finite element method in the field of biomechanics to simulate and investigate the mechanical behavior of biological tissues and organs under external forces. By constructing mathematical models of biological structures, numerical calculations can be performed to predict their responses under various conditions. Ngoc et al. (Tran et al., 2011) conducted tensile experiments on six fresh porcine transverse colon rectangular samples in both circumferential and longitudinal directions. They utilized a third-order Ogden incompressible hyperelastic model to fit the experimental data and obtain the material parameters of intestinal tissue. Additionally, they performed finite element simulation analysis to evaluate tissue displacement around the staples under leakage pressure (3.82 MPa). Building on the intestinal tissue material parameters provided by Nováček et al. (2012) conducted a finite element analysis of staple arrangement and simulated conditions with a 10 MPa pressure applied. The results indicated that a single row of staples was insufficient to resist leakage. Both studies focused on the effects of intraluminal pressure changes on the mechanical performance of intestinal tissue post-anastomosis. While finite-element simulations have extensively explored the effects of intraluminal pressure on anastomotic biomechanics, to our knowledge no prior studies have specifically focused on compression-induced damage in intestinal tissue. In this work, we introduce an initial finite-element analysis framework aimed at evaluating such damage. Clinical guidelines from China recommend that, when using a circular end-to-end anastomosis stapler for rectal tumor surgery, the final staple height after operation should be maintained at 1.5–1.8 mm (Chinese Society of Surgery, 2019). This study demonstrates that for intestinal tissue pairs of 1.5 mm–2.5 mm, 2.0 mm–2.0 mm, and 2.5 mm–1.5 mm, the safe staple height falls within the recommended range. However, for 1.5 mm–1.5 mm tissue pairs, the lower limit of the safe range is too high, while for 2.5 mm–2.5 mm tissue pairs, the safe staple height exceeds this range (Table 2). These observations suggest that adjusting staple height according to the patient’s intestinal tissue thickness may help improve clinical outcomes. These results suggest that current clinical guidelines might benefit from additional investigation and potential refinement to more effectively address variations in tissue thickness.

Numerous studies have reported no correlation between the outer diameter of circular end-to-end anastomosis stapler and the occurrence of anastomotic leakage (Park et al., 2013; Shinji et al., 2018). In this study, for symmetrical intestinal tissue pairs, the maximum equivalent stress in the lower intestinal tissue was consistently greater than that in the upper tissue. It was also observed that when the upper and lower tissues had equal thickness, the safe compression range for the lower tissue was generally smaller than that for the upper tissue, probably because the contact area between the lower tissue and the stapler was smaller than that between the upper tissue and the stapler. In the current stapler design, the cartridge must reserve space for the movement of the cutting components, resulting in a relatively smaller contact area with the tissue. Consequently, the lower tissue is subjected to higher localized stress and has a narrower safe compression range, highlighting the need for particular care to protect blood supply to the cartridge-side tissue during surgery. Therefore, the authors suggest that, without compromising functionality, reducing the inner diameter of the circular end-to-end anastomosis stapler’s contact surface and increasing its outer diameter to enlarge the contact area in the design of the circular stapler may help mitigate compression-induced damage.

The reliability of finite element analysis experiments is closely related to the material parameters used. In this study, the material parameters were derived from *ex vivo* porcine experiments (Tran et al., 2011; Liao-yuan et al., 2018). The results showed reasonable agreement with the study by Tran et al. (2011). for compression rates below 60%. For compression rates between 60% and 75%, the pressure predicted by the hyperelastic finite element model was notably higher than that observed in *ex vivo* collagen fiber experiments under stapler compression, though remaining within the same order of magnitude. These findings suggest that the results of this study may provide useful reference insights. Due to the small structure of stapler staple holes and limited computational resources, stress concentration is an unavoidable phenomenon in finite element simulations, leading to localized stress levels higher than actual conditions. Additionally, biological tissues possess compensatory abilities, where stress-induced damage and death of a small number of cells can be compensated by surrounding healthy tissue. Therefore, this study set the damage volume threshold at 5% to more accurately reflect physiological conditions while conserving computational resources. By incorporating a damage volume threshold into our finite element analysis simulations, this study offers a preliminary approach that may aid in assessing biological tissue damage.

Limitations of this study: Only static loading conditions were simulated, without considering the effects of varying compression speeds on intestinal tissue. To simplify calculations, a bonded contact model was used between the intestinal tissue and the stapler, which differs from real clinical scenarios. The material parameters were derived from *ex vivo* porcine experiments, without accounting for individualized differences such as tissue edema and fat content. Furthermore, the damage volume threshold was based on assumptions and has not been experimentally validated. Finally, physiological factors such as tissue perfusion and tensile stresses arising from tissue traction were not incorporated; these have been shown to affect anastomotic viability and leak risk (Boni et al., 2016; Lam et al., 2020).

## 5 Conclusion

By incorporating a damage volume threshold into finite element analyses, we propose a preliminary framework for estimating safe compression ranges of circular end-to-end anastomosis stapler. It was observed that when the upper and lower tissues have equal thickness, the cartridge side tissue experiences a more severe stress environment, thus requiring particular attention to protect its blood supply during surgery. Our findings suggest that tissue thickness and compression ratio influence the risk of anastomotic tissue damage. This approach may inform future stapler design and clinical practice.

## Data Availability

The original contributions presented in the study are included in the article/Supplementary Material, further inquiries can be directed to the corresponding author.
