# Supplementary material for: Evaluation of intestinal tissue safety during the compression process of circular end-to-end anastomosis stapler based on finite element simulation
Source: Front Bioeng Biotechnol. 2025 Jun 26;13:1594969. doi: 10.3389/fbioe.2025.1594969 (PMC12241024; doi:10.3389/fbioe.2025.1594969)
Supplement: Supplementary file 6 [file DataSheet1.docx]

# Stress Volume Analysis Code

*dim, VOLUME_TABLE, array, 40 ! Create an array to store the volume for each load step, totaling 40 steps

/post1 ! Enter the post-processing module
*do, step, 1, 40, 1 ! Loop through each load step
 SET, step ! Set the current load step
 ALLSEL ! Select all elements and nodes

 ! Select elements with IDs in the range 1 to 15712
 ESEL, S, ELEM, , 1, 15712
 eplo ! Plot elements to visualize the selection

 *get, edtol, elem, 0, count ! Get the total number of selected elements (edtol)
 *get, edmin, elem, 0, num, min ! Get the minimum element ID (edmin)
 ETABLE, ETAB_STRESS, s, eqv ! Define equivalent stress (von Mises stress) as a variable in the stress table

 ! Initialize equivalent stress array
 ESTRESS =
 *dim, ESTRESS,, edtol ! Define the dimensions of the ESTRESS array as the total number of selected elements
 *do, ie, edmin, edtol, 1 ! Loop through all elements (from edmin to edtol)
 *if, ESEL(ie), eq, 1, then ! If the element is selected
 *GET, von, ELEM, ie, ETAB, ETAB_STRESS ! Get the equivalent stress of the current element
 *if, von, gt, 0.0, then ! If the equivalent stress is greater than 0.0
 *if, von, lt, 0.1, then ! And less than 0.1
 ESTRESS(ie) = 1 ! Set the corresponding position in the ESTRESS array to 1 (meets condition)
 *endif
 *endif
 *endif
 *enddo

 ESEL, S, ELEM, , 1, 15712 ! Ensure only elements with IDs in the range 1 to 15712 are selected
 eplo ! Plot elements to visualize the selection
 ESEL, NONE ! Clear all selections
 *do, ie, edmin, edtol, 1 ! Loop through all elements again
 *if, ESTRESS(ie), eq, 1, then ! If the value in the ESTRESS array is 1 (element meets condition)
 ESEL, A,,, ie, ! Add the element to the selection
 *endif
 *enddo
 cm, ee3, elem ! Create a component named "ee3" to store the selected elements

 ! Calculate the total volume
 cmsel, s, ee3, elem ! Select the component "ee3" with the selected elements
 etable, evol, volu ! Define the volume variable
 ssum ! Sum the selected elements to calculate the total volume
 *get, VOLUME_TABLE(step), ssum,, item, evol ! Store the total volume in the VOLUME_TABLE array at the current step position
*enddo

! Output the volume table
*cfopen, volume_output1, txt ! Open a file to output the results
*do, step, 1, 40, 1
 *vwrite, step, VOLUME_TABLE(step)
 (F8.0, ', ', E15.6) ! Output format: first number is the step number, second number is the volume
*enddo
*cfclose

*dim, VOLUME_TABLE, array, 40 ! Create an array to store the volume for each load step, totaling 40 steps

/post1 ! Enter the post-processing module
*do, step, 1, 40, 1 ! Loop through each load step
 SET, step ! Set the current load step
 ALLSEL ! Select all elements and nodes

 ! Select elements with IDs in the range 1 to 15712
 ESEL, S, ELEM, , 1, 15712
 eplo ! Plot elements to visualize the selection

 *get, edtol, elem, 0, count ! Get the total number of selected elements (edtol)
 *get, edmin, elem, 0, num, min ! Get the minimum element ID (edmin)
 ETABLE, ETAB_STRESS, s, eqv ! Define equivalent stress (von Mises stress) as a variable in the stress table

 ! Initialize equivalent stress array
 ESTRESS =
 *dim, ESTRESS,, edtol ! Define the dimensions of the ESTRESS array as the total number of selected elements
 *do, ie, edmin, edtol, 1 ! Loop through all elements (from edmin to edtol)
 *if, ESEL(ie), eq, 1, then ! If the element is selected
 *GET, von, ELEM, ie, ETAB, ETAB_STRESS ! Get the equivalent stress of the current element
 *if, von, gt, 0.1, then ! If the equivalent stress is greater than 0.1
 *if, von, lt, 0.2, then ! And less than 0.2
 ESTRESS(ie) = 1 ! Set the corresponding position in the ESTRESS array to 1 (meets condition)
 *endif
 *endif
 *endif
 *enddo

 ESEL, S, ELEM, , 1, 15712 ! Ensure only elements with IDs in the range 1 to 15712 are selected
 eplo ! Plot elements to visualize the selection
 ESEL, NONE ! Clear all selections
 *do, ie, edmin, edtol, 1 ! Loop through all elements again
 *if, ESTRESS(ie), eq, 1, then ! If the value in the ESTRESS array is 1 (element meets condition)
 ESEL, A,,, ie, ! Add the element to the selection
 *endif
 *enddo
 cm, ee3, elem ! Create a component named "ee3" to store the selected elements

 ! Calculate the total volume
 cmsel, s, ee3, elem ! Select the component "ee3" with the selected elements
 etable, evol, volu ! Define the volume variable
 ssum ! Sum the selected elements to calculate the total volume
 *get, VOLUME_TABLE(step), ssum,, item, evol ! Store the total volume in the VOLUME_TABLE array at the current step position
*enddo

! Output the volume table
*cfopen, volume_output2, txt ! Open a file to output the results
*do, step, 1, 40, 1
 *vwrite, step, VOLUME_TABLE(step)
 (F8.0, ', ', E15.6) ! Output format: first number is the step number, second number is the volume
*enddo
*cfclose

*dim, VOLUME_TABLE, array, 40 ! Create an array to store the volume for each load step, totaling 40 steps

/post1 ! Enter the post-processing module
*do, step, 1, 40, 1 ! Loop through each load step
 SET, step ! Set the current load step
 ALLSEL ! Select all elements and nodes

 ! Select elements with IDs in the range 1 to 15712
 ESEL, S, ELEM, , 1, 15712
 eplo ! Plot elements to visualize the selection

 *get, edtol, elem, 0, count ! Get the total number of selected elements (edtol)
 *get, edmin, elem, 0, num, min ! Get the minimum element ID (edmin)
 ETABLE, ETAB_STRESS, s, eqv ! Define equivalent stress (von Mises stress) as a variable in the stress table

 ! Initialize equivalent stress array
 ESTRESS =
 *dim, ESTRESS,, edtol ! Define the dimensions of the ESTRESS array as the total number of selected elements
 *do, ie, edmin, edtol, 1 ! Loop through all elements (from edmin to edtol)
 *if, ESEL(ie), eq, 1, then ! If the element is selected
 *GET, von, ELEM, ie, ETAB, ETAB_STRESS ! Get the equivalent stress of the current element
 *if, von, gt, 0.2, then ! If the equivalent stress is greater than 0.2
 *if, von, lt, 0.3, then ! And less than 0.3
 ESTRESS(ie) = 1 ! Set the corresponding position in the ESTRESS array to 1 (meets condition)
 *endif
 *endif
 *endif
 *enddo

 ESEL, S, ELEM, , 1, 15712 ! Ensure only elements with IDs in the range 1 to 15712 are selected
 eplo ! Plot elements to visualize the selection
 ESEL, NONE ! Clear all selections
 *do, ie, edmin, edtol, 1 ! Loop through all elements again
 *if, ESTRESS(ie), eq, 1, then ! If the value in the ESTRESS array is 1 (element meets condition)
 ESEL, A,,, ie, ! Add the element to the selection
 *endif
 *enddo
 cm, ee3, elem ! Create a component named "ee3" to store the selected elements

 ! Calculate the total volume
 cmsel, s, ee3, elem ! Select the component "ee3" with the selected elements
 etable, evol, volu ! Define the volume variable
 ssum ! Sum the selected elements to calculate the total volume
 *get, VOLUME_TABLE(step), ssum,, item, evol ! Store the total volume in the VOLUME_TABLE array at the current step position
*enddo

! Output the volume table
*cfopen, volume_output3, txt ! Open a file to output the results
*do, step, 1, 40, 1
 *vwrite, step, VOLUME_TABLE(step)
 (F8.0, ', ', E15.6) ! Output format: first number is the step number, second number is the volume
*enddo
*cfclose

*dim, VOLUME_TABLE, array, 40 ! Create an array to store the volume for each load step, totaling 40 steps

/post1 ! Enter the post-processing module
*do, step, 1, 40, 1 ! Loop through each load step
 SET, step ! Set the current load step
 ALLSEL ! Select all elements and nodes

 ! Select elements with IDs in the range 1 to 15712
 ESEL, S, ELEM, , 1, 15712
 eplo ! Plot elements to visualize the selection

 *get, edtol, elem, 0, count ! Get the total number of selected elements (edtol)
 *get, edmin, elem, 0, num, min ! Get the minimum element ID (edmin)
 ETABLE, ETAB_STRESS, s, eqv ! Define equivalent stress (von Mises stress) as a variable in the stress table

 ! Initialize equivalent stress array
 ESTRESS =
 *dim, ESTRESS,, edtol ! Define the dimensions of the ESTRESS array as the total number of selected elements
 *do, ie, edmin, edtol, 1 ! Loop through all elements (from edmin to edtol)
 *if, ESEL(ie), eq, 1, then ! If the element is selected
 *GET, von, ELEM, ie, ETAB, ETAB_STRESS ! Get the equivalent stress of the current element
 *if, von, gt, 0.3, then ! If the equivalent stress is greater than 0.3
 *if, von, lt, 0.4, then ! And less than 0.4
 ESTRESS(ie) = 1 ! Set the corresponding position in the ESTRESS array to 1 (meets condition)
 *endif
 *endif
 *endif
 *enddo

 ESEL, S, ELEM, , 1, 15712 ! Ensure only elements with IDs in the range 1 to 15712 are selected
 eplo ! Plot elements to visualize the selection
 ESEL, NONE ! Clear all selections
 *do, ie, edmin, edtol, 1 ! Loop through all elements again
 *if, ESTRESS(ie), eq, 1, then ! If the value in the ESTRESS array is 1 (element meets condition)
 ESEL, A,,, ie, ! Add the element to the selection
 *endif
 *enddo
 cm, ee3, elem ! Create a component named "ee3" to store the selected elements

 ! Calculate the total volume
 cmsel, s, ee3, elem ! Select the component "ee3" with the selected elements
 etable, evol, volu ! Define the volume variable
 ssum ! Sum the selected elements to calculate the total volume
 *get, VOLUME_TABLE(step), ssum,, item, evol ! Store the total volume in the VOLUME_TABLE array at the current step position
*enddo

! Output the volume table
*cfopen, volume_output4, txt ! Open a file to output the results
*do, step, 1, 40, 1
 *vwrite, step, VOLUME_TABLE(step)
 (F8.0, ', ', E15.6) ! Output format: first number is the step number, second number is the volume
*enddo
*cfclose

*dim, VOLUME_TABLE, array, 40 ! Create an array to store the volume for each load step, totaling 40 steps

/post1 ! Enter the post-processing module
*do, step, 1, 40, 1 ! Loop through each load step
 SET, step ! Set the current load step
 ALLSEL ! Select all elements and nodes

 ! Select elements with IDs in the range 1 to 15712
 ESEL, S, ELEM, , 1, 15712
 eplo ! Plot elements to visualize the selection

 *get, edtol, elem, 0, count ! Get the total number of selected elements (edtol)
 *get, edmin, elem, 0, num, min ! Get the minimum element ID (edmin)
 ETABLE, ETAB_STRESS, s, eqv ! Define equivalent stress (von Mises stress) as a variable in the stress table

 ! Initialize equivalent stress array
 ESTRESS =
 *dim, ESTRESS,, edtol ! Define the dimensions of the ESTRESS array as the total number of selected elements
 *do, ie, edmin, edtol, 1 ! Loop through all elements (from edmin to edtol)
 *if, ESEL(ie), eq, 1, then ! If the element is selected
 *GET, von, ELEM, ie, ETAB, ETAB_STRESS ! Get the equivalent stress of the current element
 *if, von, gt, 0.4, then ! If the equivalent stress is greater than 0.4
 *if, von, lt, 0.5, then ! And less than 0.5
 ESTRESS(ie) = 1 ! Set the corresponding position in the ESTRESS array to 1 (meets condition)
 *endif
 *endif
 *endif
 *enddo

 ESEL, S, ELEM, , 1, 15712 ! Ensure only elements with IDs in the range 1 to 15712 are selected
 eplo ! Plot elements to visualize the selection
 ESEL, NONE ! Clear all selections
 *do, ie, edmin, edtol, 1 ! Loop through all elements again
 *if, ESTRESS(ie), eq, 1, then ! If the value in the ESTRESS array is 1 (element meets condition)
 ESEL, A,,, ie, ! Add the element to the selection
 *endif
 *enddo
 cm, ee3, elem ! Create a component named "ee3" to store the selected elements

 ! Calculate the total volume
 cmsel, s, ee3, elem ! Select the component "ee3" with the selected elements
 etable, evol, volu ! Define the volume variable
 ssum ! Sum the selected elements to calculate the total volume
 *get, VOLUME_TABLE(step), ssum,, item, evol ! Store the total volume in the VOLUME_TABLE array at the current step position
*enddo

! Output the volume table
*cfopen, volume_output5, txt ! Open a file to output the results
*do, step, 1, 40, 1
 *vwrite, step, VOLUME_TABLE(step)
 (F8.0, ', ', E15.6) ! Output format: first number is the step number, second number is the volume
*enddo
*cfclose

*dim, VOLUME_TABLE, array, 40 ! Create an array to store the volume for each load step, totaling 40 steps

/post1 ! Enter the post-processing module
*do, step, 1, 40, 1 ! Loop through each load step
 SET, step ! Set the current load step
 ALLSEL ! Select all elements and nodes

 ! Select elements with IDs in the range 1 to 15712
 ESEL, S, ELEM, , 1, 15712
 eplo ! Plot elements to visualize the selection

 *get, edtol, elem, 0, count ! Get the total number of selected elements (edtol)
 *get, edmin, elem, 0, num, min ! Get the minimum element ID (edmin)
 ETABLE, ETAB_STRESS, s, eqv ! Define equivalent stress (von Mises stress) as a variable in the stress table

 ! Initialize equivalent stress array
 ESTRESS =
 *dim, ESTRESS,, edtol ! Define the dimensions of the ESTRESS array as the total number of selected elements
 *do, ie, edmin, edtol, 1 ! Loop through all elements (from edmin to edtol)
 *if, ESEL(ie), eq, 1, then ! If the element is selected
 *GET, von, ELEM, ie, ETAB, ETAB_STRESS ! Get the equivalent stress of the current element
 *if, von, gt, 0.5, then ! If the equivalent stress is greater than 0.5
 *if, von, lt, 0.6, then ! And less than 0.6
 ESTRESS(ie) = 1 ! Set the corresponding position in the ESTRESS array to 1 (meets condition)
 *endif
 *endif
 *endif
 *enddo

 ESEL, S, ELEM, , 1, 15712 ! Ensure only elements with IDs in the range 1 to 15712 are selected
 eplo ! Plot elements to visualize the selection
 ESEL, NONE ! Clear all selections
 *do, ie, edmin, edtol, 1 ! Loop through all elements again
 *if, ESTRESS(ie), eq, 1, then ! If the value in the ESTRESS array is 1 (element meets condition)
 ESEL, A,,, ie, ! Add the element to the selection
 *endif
 *enddo
 cm, ee3, elem ! Create a component named "ee3" to store the selected elements

 ! Calculate the total volume
 cmsel, s, ee3, elem ! Select the component "ee3" with the selected elements
 etable, evol, volu ! Define the volume variable
 ssum ! Sum the selected elements to calculate the total volume
 *get, VOLUME_TABLE(step), ssum,, item, evol ! Store the total volume in the VOLUME_TABLE array at the current step position
*enddo

! Output the volume table
*cfopen, volume_output6, txt ! Open a file to output the results
*do, step, 1, 40, 1
 *vwrite, step, VOLUME_TABLE(step)
 (F8.0, ', ', E15.6) ! Output format: first number is the step number, second number is the volume
*enddo
*cfclose

*dim, VOLUME_TABLE, array, 40 ! Create an array to store the volume for each load step, totaling 40 steps

/post1 ! Enter the post-processing module
*do, step, 1, 40, 1 ! Loop through each load step
 SET, step ! Set the current load step
 ALLSEL ! Select all elements and nodes

 ! Select elements with IDs in the range 1 to 15712
 ESEL, S, ELEM, , 1, 15712
 eplo ! Plot elements to visualize the selection

 *get, edtol, elem, 0, count ! Get the total number of selected elements (edtol)
 *get, edmin, elem, 0, num, min ! Get the minimum element ID (edmin)
 ETABLE, ETAB_STRESS, s, eqv ! Define equivalent stress (von Mises stress) as a variable in the stress table

 ! Initialize equivalent stress array
 ESTRESS =
 *dim, ESTRESS,, edtol ! Define the dimensions of the ESTRESS array as the total number of selected elements
 *do, ie, edmin, edtol, 1 ! Loop through all elements (from edmin to edtol)
 *if, ESEL(ie), eq, 1, then ! If the element is selected
 *GET, von, ELEM, ie, ETAB, ETAB_STRESS ! Get the equivalent stress of the current element
 *if, von, gt, 0.6, then ! If the equivalent stress is greater than 0.6
 *if, von, lt, 0.7, then ! And less than 0.7
 ESTRESS(ie) = 1 ! Set the corresponding position in the ESTRESS array to 1 (meets condition)
 *endif
 *endif
 *endif
 *enddo

 ESEL, S, ELEM, , 1, 15712 ! Ensure only elements with IDs in the range 1 to 15712 are selected
 eplo ! Plot elements to visualize the selection
 ESEL, NONE ! Clear all selections
 *do, ie, edmin, edtol, 1 ! Loop through all elements again
 *if, ESTRESS(ie), eq, 1, then ! If the value in the ESTRESS array is 1 (element meets condition)
 ESEL, A,,, ie, ! Add the element to the selection
 *endif
 *enddo
 cm, ee3, elem ! Create a component named "ee3" to store the selected elements

 ! Calculate the total volume
 cmsel, s, ee3, elem ! Select the component "ee3" with the selected elements
 etable, evol, volu ! Define the volume variable
 ssum ! Sum the selected elements to calculate the total volume
 *get, VOLUME_TABLE(step), ssum,, item, evol ! Store the total volume in the VOLUME_TABLE array at the current step position
*enddo

! Output the volume table
*cfopen, volume_output7, txt ! Open a file to output the results
*do, step, 1, 40, 1
 *vwrite, step, VOLUME_TABLE(step)
 (F8.0, ', ', E15.6) ! Output format: first number is the step number, second number is the volume
*enddo
*cfclose

*dim, VOLUME_TABLE, array, 40 ! Create an array to store the volume for each load step, totaling 40 steps

/post1 ! Enter the post-processing module
*do, step, 1, 40, 1 ! Loop through each load step
 SET, step ! Set the current load step
 ALLSEL ! Select all elements and nodes

 ! Select elements with IDs in the range 1 to 15712
 ESEL, S, ELEM, , 1, 15712
 eplo ! Plot elements to visualize the selection

 *get, edtol, elem, 0, count ! Get the total number of selected elements (edtol)
 *get, edmin, elem, 0, num, min ! Get the minimum element ID (edmin)
 ETABLE, ETAB_STRESS, s, eqv ! Define equivalent stress (von Mises stress) as a variable in the stress table

 ! Initialize equivalent stress array
 ESTRESS =
 *dim, ESTRESS,, edtol ! Define the dimensions of the ESTRESS array as the total number of selected elements
 *do, ie, edmin, edtol, 1 ! Loop through all elements (from edmin to edtol)
 *if, ESEL(ie), eq, 1, then ! If the element is selected
 *GET, von, ELEM, ie, ETAB, ETAB_STRESS ! Get the equivalent stress of the current element
 *if, von, gt, 0.7, then ! If the equivalent stress is greater than 0.7
 *if, von, lt, 0.8, then ! And less than 0.8
 ESTRESS(ie) = 1 ! Set the corresponding position in the ESTRESS array to 1 (meets condition)
 *endif
 *endif
 *endif
 *enddo

 ESEL, S, ELEM, , 1, 15712 ! Ensure only elements with IDs in the range 1 to 15712 are selected
 eplo ! Plot elements to visualize the selection
 ESEL, NONE ! Clear all selections
 *do, ie, edmin, edtol, 1 ! Loop through all elements again
 *if, ESTRESS(ie), eq, 1, then ! If the value in the ESTRESS array is 1 (element meets condition)
 ESEL, A,,, ie, ! Add the element to the selection
 *endif
 *enddo
 cm, ee3, elem ! Create a component named "ee3" to store the selected elements

 ! Calculate the total volume
 cmsel, s, ee3, elem ! Select the component "ee3" with the selected elements
 etable, evol, volu ! Define the volume variable
 ssum ! Sum the selected elements to calculate the total volume
 *get, VOLUME_TABLE(step), ssum,, item, evol ! Store the total volume in the VOLUME_TABLE array at the current step position
*enddo

! Output the volume table
*cfopen, volume_output8, txt ! Open a file to output the results
*do, step, 1, 40, 1
 *vwrite, step, VOLUME_TABLE(step)
 (F8.0, ', ', E15.6) ! Output format: first number is the step number, second number is the volume
*enddo
*cfclose

*dim, VOLUME_TABLE, array, 40 ! Create an array to store the volume for each load step, totaling 40 steps

/post1 ! Enter the post-processing module
*do, step, 1, 40, 1 ! Loop through each load step
 SET, step ! Set the current load step
 ALLSEL ! Select all elements and nodes

 ! Select elements with IDs in the range 1 to 15712
 ESEL, S, ELEM, , 1, 15712
 eplo ! Plot elements to visualize the selection

 *get, edtol, elem, 0, count ! Get the total number of selected elements (edtol)
 *get, edmin, elem, 0, num, min ! Get the minimum element ID (edmin)
 ETABLE, ETAB_STRESS, s, eqv ! Define equivalent stress (von Mises stress) as a variable in the stress table

 ! Initialize equivalent stress array
 ESTRESS =
 *dim, ESTRESS,, edtol ! Define the dimensions of the ESTRESS array as the total number of selected elements
 *do, ie, edmin, edtol, 1 ! Loop through all elements (from edmin to edtol)
 *if, ESEL(ie), eq, 1, then ! If the element is selected
 *GET, von, ELEM, ie, ETAB, ETAB_STRESS ! Get the equivalent stress of the current element
 *if, von, gt, 0.8, then ! If the equivalent stress is greater than 0.8
 *if, von, lt, 0.9, then ! And less than 0.9
 ESTRESS(ie) = 1 ! Set the corresponding position in the ESTRESS array to 1 (meets condition)
 *endif
 *endif
 *endif
 *enddo

 ESEL, S, ELEM, , 1, 15712 ! Ensure only elements with IDs in the range 1 to 15712 are selected
 eplo ! Plot elements to visualize the selection
 ESEL, NONE ! Clear all selections
 *do, ie, edmin, edtol, 1 ! Loop through all elements again
 *if, ESTRESS(ie), eq, 1, then ! If the value in the ESTRESS array is 1 (element meets condition)
 ESEL, A,,, ie, ! Add the element to the selection
 *endif
 *enddo
 cm, ee3, elem ! Create a component named "ee3" to store the selected elements

 ! Calculate the total volume
 cmsel, s, ee3, elem ! Select the component "ee3" with the selected elements
 etable, evol, volu ! Define the volume variable
 ssum ! Sum the selected elements to calculate the total volume
 *get, VOLUME_TABLE(step), ssum,, item, evol ! Store the total volume in the VOLUME_TABLE array at the current step position
*enddo

! Output the volume table
*cfopen, volume_output9, txt ! Open a file to output the results
*do, step, 1, 40, 1
 *vwrite, step, VOLUME_TABLE(step)
 (F8.0, ', ', E15.6) ! Output format: first number is the step number, second number is the volume
*enddo
*cfclose

*dim, VOLUME_TABLE, array, 40 ! Create an array to store the volume for each load step, totaling 40 steps

/post1 ! Enter the post-processing module
*do, step, 1, 40, 1 ! Loop through each load step
 SET, step ! Set the current load step
 ALLSEL ! Select all elements and nodes

 ! Select elements with IDs in the range 1 to 15712
 ESEL, S, ELEM, , 1, 15712
 eplo ! Plot elements to visualize the selection

 *get, edtol, elem, 0, count ! Get the total number of selected elements (edtol)
 *get, edmin, elem, 0, num, min ! Get the minimum element ID (edmin)
 ETABLE, ETAB_STRESS, s, eqv ! Define equivalent stress (von Mises stress) as a variable in the stress table

 ! Initialize equivalent stress array
 ESTRESS =
 *dim, ESTRESS,, edtol ! Define the dimensions of the ESTRESS array as the total number of selected elements
 *do, ie, edmin, edtol, 1 ! Loop through all elements (from edmin to edtol)
 *if, ESEL(ie), eq, 1, then ! If the element is selected
 *GET, von, ELEM, ie, ETAB, ETAB_STRESS ! Get the equivalent stress of the current element
 *if, von, gt, 0.9, then ! If the equivalent stress is greater than 0.9
 *if, von, lt, 1.0, then ! And less than 1.0
 ESTRESS(ie) = 1 ! Set the corresponding position in the ESTRESS array to 1 (meets condition)
 *endif
 *endif
 *endif
 *enddo

 ESEL, S, ELEM, , 1, 15712 ! Ensure only elements with IDs in the range 1 to 15712 are selected
 eplo ! Plot elements to visualize the selection
 ESEL, NONE ! Clear all selections
 *do, ie, edmin, edtol, 1 ! Loop through all elements again
 *if, ESTRESS(ie), eq, 1, then ! If the value in the ESTRESS array is 1 (element meets condition)
 ESEL, A,,, ie, ! Add the element to the selection
 *endif
 *enddo
 cm, ee3, elem ! Create a component named "ee3" to store the selected elements

 ! Calculate the total volume
 cmsel, s, ee3, elem ! Select the component "ee3" with the selected elements
 etable, evol, volu ! Define the volume variable
 ssum ! Sum the selected elements to calculate the total volume
 *get, VOLUME_TABLE(step), ssum,, item, evol ! Store the total volume in the VOLUME_TABLE array at the current step position
*enddo

! Output the volume table
*cfopen, volume_output10, txt ! Open a file to output the results
*do, step, 1, 40, 1
 *vwrite, step, VOLUME_TABLE(step)
 (F8.0, ', ', E15.6) ! Output format: first number is the step number, second number is the volume
*enddo
*cfclose

*dim, VOLUME_TABLE, array, 40 ! Create an array to store the volume for each load step, totaling 40 steps

/post1 ! Enter the post-processing module
*do, step, 1, 40, 1 ! Loop through each load step
 SET, step ! Set the current load step
 ALLSEL ! Select all elements and nodes

 ! Select elements with IDs in the range 1 to 15712
 ESEL, S, ELEM, , 1, 15712
 eplo ! Plot elements to visualize the selection

 *get, edtol, elem, 0, count ! Get the total number of selected elements (edtol)
 *get, edmin, elem, 0, num, min ! Get the minimum element ID (edmin)
 ETABLE, ETAB_STRESS, s, eqv ! Define equivalent stress (von Mises stress) as a variable in the stress table

 ! Initialize equivalent stress array
 ESTRESS =
 *dim, ESTRESS,, edtol ! Define the dimensions of the ESTRESS array as the total number of selected elements
 *do, ie, edmin, edtol, 1 ! Loop through all elements (from edmin to edtol)
 *if, ESEL(ie), eq, 1, then ! If the element is selected
 *GET, von, ELEM, ie, ETAB, ETAB_STRESS ! Get the equivalent stress of the current element
 *if, von, gt, 1.0, then ! If the equivalent stress is greater than 1.0
 *if, von, lt, 1.1, then ! And less than 1.1
 ESTRESS(ie) = 1 ! Set the corresponding position in the ESTRESS array to 1 (meets condition)
 *endif
 *endif
 *endif
 *enddo

 ESEL, S, ELEM, , 1, 15712 ! Ensure only elements with IDs in the range 1 to 15712 are selected
 eplo ! Plot elements to visualize the selection
 ESEL, NONE ! Clear all selections
 *do, ie, edmin, edtol, 1 ! Loop through all elements again
 *if, ESTRESS(ie), eq, 1, then ! If the value in the ESTRESS array is 1 (element meets condition)
 ESEL, A,,, ie, ! Add the element to the selection
 *endif
 *enddo
 cm, ee3, elem ! Create a component named "ee3" to store the selected elements

 ! Calculate the total volume
 cmsel, s, ee3, elem ! Select the component "ee3" with the selected elements
 etable, evol, volu ! Define the volume variable
 ssum ! Sum the selected elements to calculate the total volume
 *get, VOLUME_TABLE(step), ssum,, item, evol ! Store the total volume in the VOLUME_TABLE array at the current step position
*enddo

! Output the volume table
*cfopen, volume_output11, txt ! Open a file to output the results
*do, step, 1, 40, 1
 *vwrite, step, VOLUME_TABLE(step)
 (F8.0, ', ', E15.6) ! Output format: first number is the step number, second number is the volume
*enddo
*cfclose

*dim, VOLUME_TABLE, array, 40 ! Create an array to store the volume for each load step, totaling 40 steps

/post1 ! Enter the post-processing module
*do, step, 1, 40, 1 ! Loop through each load step
 SET, step ! Set the current load step
 ALLSEL ! Select all elements and nodes

 ! Select elements with IDs in the range 1 to 15712
 ESEL, S, ELEM, , 1, 15712
 eplo ! Plot elements to visualize the selection

 *get, edtol, elem, 0, count ! Get the total number of selected elements (edtol)
 *get, edmin, elem, 0, num, min ! Get the minimum element ID (edmin)
 ETABLE, ETAB_STRESS, s, eqv ! Define equivalent stress (von Mises stress) as a variable in the stress table

 ! Initialize equivalent stress array
 ESTRESS =
 *dim, ESTRESS,, edtol ! Define the dimensions of the ESTRESS array as the total number of selected elements
 *do, ie, edmin, edtol, 1 ! Loop through all elements (from edmin to edtol)
 *if, ESEL(ie), eq, 1, then ! If the element is selected
 *GET, von, ELEM, ie, ETAB, ETAB_STRESS ! Get the equivalent stress of the current element
 *if, von, gt, 1.1, then ! If the equivalent stress is greater than 1.1
 *if, von, lt, 1.2, then ! And less than 1.2
 ESTRESS(ie) = 1 ! Set the corresponding position in the ESTRESS array to 1 (meets condition)
 *endif
 *endif
 *endif
 *enddo

 ESEL, S, ELEM, , 1, 15712 ! Ensure only elements with IDs in the range 1 to 15712 are selected
 eplo ! Plot elements to visualize the selection
 ESEL, NONE ! Clear all selections
 *do, ie, edmin, edtol, 1 ! Loop through all elements again
 *if, ESTRESS(ie), eq, 1, then ! If the value in the ESTRESS array is 1 (element meets condition)
 ESEL, A,,, ie, ! Add the element to the selection
 *endif
 *enddo
 cm, ee3, elem ! Create a component named "ee3" to store the selected elements

 ! Calculate the total volume
 cmsel, s, ee3, elem ! Select the component "ee3" with the selected elements
 etable, evol, volu ! Define the volume variable
 ssum ! Sum the selected elements to calculate the total volume
 *get, VOLUME_TABLE(step), ssum,, item, evol ! Store the total volume in the VOLUME_TABLE array at the current step position
*enddo

! Output the volume table
*cfopen, volume_output12, txt ! Open a file to output the results
*do, step, 1, 40, 1
 *vwrite, step, VOLUME_TABLE(step)
 (F8.0, ', ', E15.6) ! Output format: first number is the step number, second number is the volume
*enddo
*cfclose

*dim, VOLUME_TABLE, array, 40 ! Create an array to store the volume for each load step, totaling 40 steps

/post1 ! Enter the post-processing module
*do, step, 1, 40, 1 ! Loop through each load step
 SET, step ! Set the current load step
 ALLSEL ! Select all elements and nodes

 ! Select elements with IDs in the range 1 to 15712
 ESEL, S, ELEM, , 1, 15712
 eplo ! Plot elements to visualize the selection

 *get, edtol, elem, 0, count ! Get the total number of selected elements (edtol)
 *get, edmin, elem, 0, num, min ! Get the minimum element ID (edmin)
 ETABLE, ETAB_STRESS, s, eqv ! Define equivalent stress (von Mises stress) as a variable in the stress table

 ! Initialize equivalent stress array
 ESTRESS =
 *dim, ESTRESS,, edtol ! Define the dimensions of the ESTRESS array as the total number of selected elements
 *do, ie, edmin, edtol, 1 ! Loop through all elements (from edmin to edtol)
 *if, ESEL(ie), eq, 1, then ! If the element is selected
 *GET, von, ELEM, ie, ETAB, ETAB_STRESS ! Get the equivalent stress of the current element
 *if, von, gt, 1.2, then ! If the equivalent stress is greater than 1.2
 *if, von, lt, 1.3, then ! And less than 1.3
 ESTRESS(ie) = 1 ! Set the corresponding position in the ESTRESS array to 1 (meets condition)
 *endif
 *endif
 *endif
 *enddo

 ESEL, S, ELEM, , 1, 15712 ! Ensure only elements with IDs in the range 1 to 15712 are selected
 eplo ! Plot elements to visualize the selection
 ESEL, NONE ! Clear all selections
 *do, ie, edmin, edtol, 1 ! Loop through all elements again
 *if, ESTRESS(ie), eq, 1, then ! If the value in the ESTRESS array is 1 (element meets condition)
 ESEL, A,,, ie, ! Add the element to the selection
 *endif
 *enddo
 cm, ee3, elem ! Create a component named "ee3" to store the selected elements

 ! Calculate the total volume
 cmsel, s, ee3, elem ! Select the component "ee3" with the selected elements
 etable, evol, volu ! Define the volume variable
 ssum ! Sum the selected elements to calculate the total volume
 *get, VOLUME_TABLE(step), ssum,, item, evol ! Store the total volume in the VOLUME_TABLE array at the current step position
*enddo

! Output the volume table
*cfopen, volume_output13, txt ! Open a file to output the results
*do, step, 1, 40, 1
 *vwrite, step, VOLUME_TABLE(step)
 (F8.0, ', ', E15.6) ! Output format: first number is the step number, second number is the volume
*enddo
*cfclose

*dim, VOLUME_TABLE, array, 40 ! Create an array to store the volume for each load step, totaling 40 steps

/post1 ! Enter the post-processing module
*do, step, 1, 40, 1 ! Loop through each load step
 SET, step ! Set the current load step
 ALLSEL ! Select all elements and nodes

 ! Select elements with IDs in the range 1 to 15712
 ESEL, S, ELEM, , 1, 15712
 eplo ! Plot elements to visualize the selection

 *get, edtol, elem, 0, count ! Get the total number of selected elements (edtol)
 *get, edmin, elem, 0, num, min ! Get the minimum element ID (edmin)
 ETABLE, ETAB_STRESS, s, eqv ! Define equivalent stress (von Mises stress) as a variable in the stress table

 ! Initialize equivalent stress array
 ESTRESS =
 *dim, ESTRESS,, edtol ! Define the dimensions of the ESTRESS array as the total number of selected elements
 *do, ie, edmin, edtol, 1 ! Loop through all elements (from edmin to edtol)
 *if, ESEL(ie), eq, 1, then ! If the element is selected
 *GET, von, ELEM, ie, ETAB, ETAB_STRESS ! Get the equivalent stress of the current element
 *if, von, gt, 1.3, then ! If the equivalent stress is greater than 1.3
 *if, von, lt, 1.4, then ! And less than 1.4
 ESTRESS(ie) = 1 ! Set the corresponding position in the ESTRESS array to 1 (meets condition)
 *endif
 *endif
 *endif
 *enddo

 ESEL, S, ELEM, , 1, 15712 ! Ensure only elements with IDs in the range 1 to 15712 are selected
 eplo ! Plot elements to visualize the selection
 ESEL, NONE ! Clear all selections
 *do, ie, edmin, edtol, 1 ! Loop through all elements again
 *if, ESTRESS(ie), eq, 1, then ! If the value in the ESTRESS array is 1 (element meets condition)
 ESEL, A,,, ie, ! Add the element to the selection
 *endif
 *enddo
 cm, ee3, elem ! Create a component named "ee3" to store the selected elements

 ! Calculate the total volume
 cmsel, s, ee3, elem ! Select the component "ee3" with the selected elements
 etable, evol, volu ! Define the volume variable
 ssum ! Sum the selected elements to calculate the total volume
 *get, VOLUME_TABLE(step), ssum,, item, evol ! Store the total volume in the VOLUME_TABLE array at the current step position
*enddo

! Output the volume table
*cfopen, volume_output14, txt ! Open a file to output the results
*do, step, 1, 40, 1
 *vwrite, step, VOLUME_TABLE(step)
 (F8.0, ', ', E15.6) ! Output format: first number is the step number, second number is the volume
*enddo
*cfclose

*dim, VOLUME_TABLE, array, 40 ! Create an array to store the volume for each load step, totaling 40 steps

/post1 ! Enter the post-processing module
*do, step, 1, 40, 1 ! Loop through each load step
 SET, step ! Set the current load step
 ALLSEL ! Select all elements and nodes

 ! Select elements with IDs in the range 1 to 15712
 ESEL, S, ELEM, , 1, 15712
 eplo ! Plot elements to visualize the selection

 *get, edtol, elem, 0, count ! Get the total number of selected elements (edtol)
 *get, edmin, elem, 0, num, min ! Get the minimum element ID (edmin)
 ETABLE, ETAB_STRESS, s, eqv ! Define equivalent stress (von Mises stress) as a variable in the stress table

 ! Initialize equivalent stress array
 ESTRESS =
 *dim, ESTRESS,, edtol ! Define the dimensions of the ESTRESS array as the total number of selected elements
 *do, ie, edmin, edtol, 1 ! Loop through all elements (from edmin to edtol)
 *if, ESEL(ie), eq, 1, then ! If the element is selected
 *GET, von, ELEM, ie, ETAB, ETAB_STRESS ! Get the equivalent stress of the current element
 *if, von, gt, 1.4, then ! If the equivalent stress is greater than 1.4
 *if, von, lt, 1.5, then ! And less than 1.5
 ESTRESS(ie) = 1 ! Set the corresponding position in the ESTRESS array to 1 (meets condition)
 *endif
 *endif
 *endif
 *enddo

 ESEL, S, ELEM, , 1, 15712 ! Ensure only elements with IDs in the range 1 to 15712 are selected
 eplo ! Plot elements to visualize the selection
 ESEL, NONE ! Clear all selections
 *do, ie, edmin, edtol, 1 ! Loop through all elements again
 *if, ESTRESS(ie), eq, 1, then ! If the value in the ESTRESS array is 1 (element meets condition)
 ESEL, A,,, ie, ! Add the element to the selection
 *endif
 *enddo
 cm, ee3, elem ! Create a component named "ee3" to store the selected elements

 ! Calculate the total volume
 cmsel, s, ee3, elem ! Select the component "ee3" with the selected elements
 etable, evol, volu ! Define the volume variable
 ssum ! Sum the selected elements to calculate the total volume
 *get, VOLUME_TABLE(step), ssum,, item, evol ! Store the total volume in the VOLUME_TABLE array at the current step position
*enddo

! Output the volume table
*cfopen, volume_output15, txt ! Open a file to output the results
*do, step, 1, 40, 1
 *vwrite, step, VOLUME_TABLE(step)
 (F8.0, ', ', E15.6) ! Output format: first number is the step number, second number is the volume
*enddo
*cfclose

*dim, VOLUME_TABLE, array, 40 ! Create an array to store the volume for each load step, totaling 40 steps

/post1 ! Enter the post-processing module
*do, step, 1, 40, 1 ! Loop through each load step
 SET, step ! Set the current load step
 ALLSEL ! Select all elements and nodes

 ! Select elements with IDs in the range 1 to 15712
 ESEL, S, ELEM, , 1, 15712
 eplo ! Plot elements to visualize the selection

 *get, edtol, elem, 0, count ! Get the total number of selected elements (edtol)
 *get, edmin, elem, 0, num, min ! Get the minimum element ID (edmin)
 ETABLE, ETAB_STRESS, s, eqv ! Define equivalent stress (von Mises stress) as a variable in the stress table

 ! Initialize equivalent stress array
 ESTRESS =
 *dim, ESTRESS,, edtol ! Define the dimensions of the ESTRESS array as the total number of selected elements
 *do, ie, edmin, edtol, 1 ! Loop through all elements (from edmin to edtol)
 *if, ESEL(ie), eq, 1, then ! If the element is selected
 *GET, von, ELEM, ie, ETAB, ETAB_STRESS ! Get the equivalent stress of the current element
 *if, von, gt, 1.5, then ! If the equivalent stress is greater than 1.5
 *if, von, lt, 1.6, then ! And less than 1.6
 ESTRESS(ie) = 1 ! Set the corresponding position in the ESTRESS array to 1 (meets condition)
 *endif
 *endif
 *endif
 *enddo

 ESEL, S, ELEM, , 1, 15712 ! Ensure only elements with IDs in the range 1 to 15712 are selected
 eplo ! Plot elements to visualize the selection
 ESEL, NONE ! Clear all selections
 *do, ie, edmin, edtol, 1 ! Loop through all elements again
 *if, ESTRESS(ie), eq, 1, then ! If the value in the ESTRESS array is 1 (element meets condition)
 ESEL, A,,, ie, ! Add the element to the selection
 *endif
 *enddo
 cm, ee3, elem ! Create a component named "ee3" to store the selected elements

 ! Calculate the total volume
 cmsel, s, ee3, elem ! Select the component "ee3" with the selected elements
 etable, evol, volu ! Define the volume variable
 ssum ! Sum the selected elements to calculate the total volume
 *get, VOLUME_TABLE(step), ssum,, item, evol ! Store the total volume in the VOLUME_TABLE array at the current step position
*enddo

! Output the volume table
*cfopen, volume_output16, txt ! Open a file to output the results
*do, step, 1, 40, 1
 *vwrite, step, VOLUME_TABLE(step)
 (F8.0, ', ', E15.6) ! Output format: first number is the step number, second number is the volume
*enddo
*cfclose

*dim, VOLUME_TABLE, array, 40 ! Create an array to store the volume for each load step, totaling 40 steps

/post1 ! Enter the post-processing module
*do, step, 1, 40, 1 ! Loop through each load step
 SET, step ! Set the current load step
 ALLSEL ! Select all elements and nodes

 ! Select elements with IDs in the range 1 to 15712
 ESEL, S, ELEM, , 1, 15712
 eplo ! Plot elements to visualize the selection

 *get, edtol, elem, 0, count ! Get the total number of selected elements (edtol)
 *get, edmin, elem, 0, num, min ! Get the minimum element ID (edmin)
 ETABLE, ETAB_STRESS, s, eqv ! Define equivalent stress (von Mises stress) as a variable in the stress table

 ! Initialize equivalent stress array
 ESTRESS =
 *dim, ESTRESS,, edtol ! Define the dimensions of the ESTRESS array as the total number of selected elements
 *do, ie, edmin, edtol, 1 ! Loop through all elements (from edmin to edtol)
 *if, ESEL(ie), eq, 1, then ! If the element is selected
 *GET, von, ELEM, ie, ETAB, ETAB_STRESS ! Get the equivalent stress of the current element
 *if, von, gt, 1.6, then ! If the equivalent stress is greater than 1.6
 *if, von, lt, 1.7, then ! And less than 1.7
 ESTRESS(ie) = 1 ! Set the corresponding position in the ESTRESS array to 1 (meets condition)
 *endif
 *endif
 *endif
 *enddo

 ESEL, S, ELEM, , 1, 15712 ! Ensure only elements with IDs in the range 1 to 15712 are selected
 eplo ! Plot elements to visualize the selection
 ESEL, NONE ! Clear all selections
 *do, ie, edmin, edtol, 1 ! Loop through all elements again
 *if, ESTRESS(ie), eq, 1, then ! If the value in the ESTRESS array is 1 (element meets condition)
 ESEL, A,,, ie, ! Add the element to the selection
 *endif
 *enddo
 cm, ee3, elem ! Create a component named "ee3" to store the selected elements

 ! Calculate the total volume
 cmsel, s, ee3, elem ! Select the component "ee3" with the selected elements
 etable, evol, volu ! Define the volume variable
 ssum ! Sum the selected elements to calculate the total volume
 *get, VOLUME_TABLE(step), ssum,, item, evol ! Store the total volume in the VOLUME_TABLE array at the current step position
*enddo

! Output the volume table
*cfopen, volume_output17, txt ! Open a file to output the results
*do, step, 1, 40, 1
 *vwrite, step, VOLUME_TABLE(step)
 (F8.0, ', ', E15.6) ! Output format: first number is the step number, second number is the volume
*enddo
*cfclose

*dim, VOLUME_TABLE, array, 40 ! Create an array to store the volume for each load step, totaling 40 steps

/post1 ! Enter the post-processing module
*do, step, 1, 40, 1 ! Loop through each load step
 SET, step ! Set the current load step
 ALLSEL ! Select all elements and nodes

 ! Select elements with IDs in the range 1 to 15712
 ESEL, S, ELEM, , 1, 15712
 eplo ! Plot elements to visualize the selection

 *get, edtol, elem, 0, count ! Get the total number of selected elements (edtol)
 *get, edmin, elem, 0, num, min ! Get the minimum element ID (edmin)
 ETABLE, ETAB_STRESS, s, eqv ! Define equivalent stress (von Mises stress) as a variable in the stress table

 ! Initialize equivalent stress array
 ESTRESS =
 *dim, ESTRESS,, edtol ! Define the dimensions of the ESTRESS array as the total number of selected elements
 *do, ie, edmin, edtol, 1 ! Loop through all elements (from edmin to edtol)
 *if, ESEL(ie), eq, 1, then ! If the element is selected
 *GET, von, ELEM, ie, ETAB, ETAB_STRESS ! Get the equivalent stress of the current element
 *if, von, gt, 1.7, then ! If the equivalent stress is greater than 1.7
 *if, von, lt, 1.8, then ! And less than 1.8
 ESTRESS(ie) = 1 ! Set the corresponding position in the ESTRESS array to 1 (meets condition)
 *endif
 *endif
 *endif
 *enddo

 ESEL, S, ELEM, , 1, 15712 ! Ensure only elements with IDs in the range 1 to 15712 are selected
 eplo ! Plot elements to visualize the selection
 ESEL, NONE ! Clear all selections
 *do, ie, edmin, edtol, 1 ! Loop through all elements again
 *if, ESTRESS(ie), eq, 1, then ! If the value in the ESTRESS array is 1 (element meets condition)
 ESEL, A,,, ie, ! Add the element to the selection
 *endif
 *enddo
 cm, ee3, elem ! Create a component named "ee3" to store the selected elements

 ! Calculate the total volume
 cmsel, s, ee3, elem ! Select the component "ee3" with the selected elements
 etable, evol, volu ! Define the volume variable
 ssum ! Sum the selected elements to calculate the total volume
 *get, VOLUME_TABLE(step), ssum,, item, evol ! Store the total volume in the VOLUME_TABLE array at the current step position
*enddo

! Output the volume table
*cfopen, volume_output18, txt ! Open a file to output the results
*do, step, 1, 40, 1
 *vwrite, step, VOLUME_TABLE(step)
 (F8.0, ', ', E15.6) ! Output format: first number is the step number, second number is the volume
*enddo
*cfclose

*dim, VOLUME_TABLE, array, 40 ! Create an array to store the volume for each load step, totaling 40 steps

/post1 ! Enter the post-processing module
*do, step, 1, 40, 1 ! Loop through each load step
 SET, step ! Set the current load step
 ALLSEL ! Select all elements and nodes

 ! Select elements with IDs in the range 1 to 15712
 ESEL, S, ELEM, , 1, 15712
 eplo ! Plot elements to visualize the selection

 *get, edtol, elem, 0, count ! Get the total number of selected elements (edtol)
 *get, edmin, elem, 0, num, min ! Get the minimum element ID (edmin)
 ETABLE, ETAB_STRESS, s, eqv ! Define equivalent stress (von Mises stress) as a variable in the stress table

 ! Initialize equivalent stress array
 ESTRESS =
 *dim, ESTRESS,, edtol ! Define the dimensions of the ESTRESS array as the total number of selected elements
 *do, ie, edmin, edtol, 1 ! Loop through all elements (from edmin to edtol)
 *if, ESEL(ie), eq, 1, then ! If the element is selected
 *GET, von, ELEM, ie, ETAB, ETAB_STRESS ! Get the equivalent stress of the current element
 *if, von, gt, 1.8, then ! If the equivalent stress is greater than 1.8
 *if, von, lt, 1.9, then ! And less than 1.9
 ESTRESS(ie) = 1 ! Set the corresponding position in the ESTRESS array to 1 (meets condition)
 *endif
 *endif
 *endif
 *enddo

 ESEL, S, ELEM, , 1, 15712 ! Ensure only elements with IDs in the range 1 to 15712 are selected
 eplo ! Plot elements to visualize the selection
 ESEL, NONE ! Clear all selections
 *do, ie, edmin, edtol, 1 ! Loop through all elements again
 *if, ESTRESS(ie), eq, 1, then ! If the value in the ESTRESS array is 1 (element meets condition)
 ESEL, A,,, ie, ! Add the element to the selection
 *endif
 *enddo
 cm, ee3, elem ! Create a component named "ee3" to store the selected elements

 ! Calculate the total volume
 cmsel, s, ee3, elem ! Select the component "ee3" with the selected elements
 etable, evol, volu ! Define the volume variable
 ssum ! Sum the selected elements to calculate the total volume
 *get, VOLUME_TABLE(step), ssum,, item, evol ! Store the total volume in the VOLUME_TABLE array at the current step position
*enddo

! Output the volume table
*cfopen, volume_output19, txt ! Open a file to output the results
*do, step, 1, 40, 1
 *vwrite, step, VOLUME_TABLE(step)
 (F8.0, ', ', E15.6) ! Output format: first number is the step number, second number is the volume
*enddo
*cfclose

*dim, VOLUME_TABLE, array, 40 ! Create an array to store the volume for each load step, totaling 40 steps

/post1 ! Enter the post-processing module
*do, step, 1, 40, 1 ! Loop through each load step
 SET, step ! Set the current load step
 ALLSEL ! Select all elements and nodes

 ! Select elements with IDs in the range 1 to 15712
 ESEL, S, ELEM, , 1, 15712
 eplo ! Plot elements to visualize the selection

 *get, edtol, elem, 0, count ! Get the total number of selected elements (edtol)
 *get, edmin, elem, 0, num, min ! Get the minimum element ID (edmin)
 ETABLE, ETAB_STRESS, s, eqv ! Define equivalent stress (von Mises stress) as a variable in the stress table

 ! Initialize equivalent stress array
 ESTRESS =
 *dim, ESTRESS,, edtol ! Define the dimensions of the ESTRESS array as the total number of selected elements
 *do, ie, edmin, edtol, 1 ! Loop through all elements (from edmin to edtol)
 *if, ESEL(ie), eq, 1, then ! If the element is selected
 *GET, von, ELEM, ie, ETAB, ETAB_STRESS ! Get the equivalent stress of the current element
 *if, von, gt, 1.9, then ! If the equivalent stress is greater than 1.9
 *if, von, lt, 2.0, then ! And less than 2.0
 ESTRESS(ie) = 1 ! Set the corresponding position in the ESTRESS array to 1 (meets condition)
 *endif
 *endif
 *endif
 *enddo

 ESEL, S, ELEM, , 1, 15712 ! Ensure only elements with IDs in the range 1 to 15712 are selected
 eplo ! Plot elements to visualize the selection
 ESEL, NONE ! Clear all selections
 *do, ie, edmin, edtol, 1 ! Loop through all elements again
 *if, ESTRESS(ie), eq, 1, then ! If the value in the ESTRESS array is 1 (element meets condition)
 ESEL, A,,, ie, ! Add the element to the selection
 *endif
 *enddo
 cm, ee3, elem ! Create a component named "ee3" to store the selected elements

 ! Calculate the total volume
 cmsel, s, ee3, elem ! Select the component "ee3" with the selected elements
 etable, evol, volu ! Define the volume variable
 ssum ! Sum the selected elements to calculate the total volume
 *get, VOLUME_TABLE(step), ssum,, item, evol ! Store the total volume in the VOLUME_TABLE array at the current step position
*enddo

! Output the volume table
*cfopen, volume_output20, txt ! Open a file to output the results
*do, step, 1, 40, 1
 *vwrite, step, VOLUME_TABLE(step)
 (F8.0, ', ', E15.6) ! Output format: first number is the step number, second number is the volume
*enddo
*cfclose

*dim, VOLUME_TABLE, array, 40 ! Create an array to store the volume for each load step, totaling 40 steps

/post1 ! Enter the post-processing module
*do, step, 1, 40, 1 ! Loop through each load step
 SET, step ! Set the current load step
 ALLSEL ! Select all elements and nodes

 ! Select elements with IDs in the range 1 to 15712
 ESEL, S, ELEM, , 1, 15712
 eplo ! Plot elements to visualize the selection

 *get, edtol, elem, 0, count ! Get the total number of selected elements (edtol)
 *get, edmin, elem, 0, num, min ! Get the minimum element ID (edmin)
 ETABLE, ETAB_STRESS, s, eqv ! Define equivalent stress (von Mises stress) as a variable in the stress table

 ! Initialize equivalent stress array
 ESTRESS =
 *dim, ESTRESS,, edtol ! Define the dimensions of the ESTRESS array as the total number of selected elements
 *do, ie, edmin, edtol, 1 ! Loop through all elements (from edmin to edtol)
 *if, ESEL(ie), eq, 1, then ! If the element is selected
 *GET, von, ELEM, ie, ETAB, ETAB_STRESS ! Get the equivalent stress of the current element
 *if, von, gt, 2.0, then ! If the equivalent stress is greater than 2.0
 *if, von, lt, 2.1, then ! And less than 2.1
 ESTRESS(ie) = 1 ! Set the corresponding position in the ESTRESS array to 1 (meets condition)
 *endif
 *endif
 *endif
 *enddo

 ESEL, S, ELEM, , 1, 15712 ! Ensure only elements with IDs in the range 1 to 15712 are selected
 eplo ! Plot elements to visualize the selection
 ESEL, NONE ! Clear all selections
 *do, ie, edmin, edtol, 1 ! Loop through all elements again
 *if, ESTRESS(ie), eq, 1, then ! If the value in the ESTRESS array is 1 (element meets condition)
 ESEL, A,,, ie, ! Add the element to the selection
 *endif
 *enddo
 cm, ee3, elem ! Create a component named "ee3" to store the selected elements

 ! Calculate the total volume
 cmsel, s, ee3, elem ! Select the component "ee3" with the selected elements
 etable, evol, volu ! Define the volume variable
 ssum ! Sum the selected elements to calculate the total volume
 *get, VOLUME_TABLE(step), ssum,, item, evol ! Store the total volume in the VOLUME_TABLE array at the current step position
*enddo

! Output the volume table
*cfopen, volume_output21, txt ! Open a file to output the results
*do, step, 1, 40, 1
 *vwrite, step, VOLUME_TABLE(step)
 (F8.0, ', ', E15.6) ! Output format: first number is the step number, second number is the volume
*enddo
*cfclose

*dim, VOLUME_TABLE, array, 40 ! Create an array to store the volume for each load step, totaling 40 steps

/post1 ! Enter the post-processing module
*do, step, 1, 40, 1 ! Loop through each load step
 SET, step ! Set the current load step
 ALLSEL ! Select all elements and nodes

 ! Select elements with IDs in the range 1 to 15712
 ESEL, S, ELEM, , 1, 15712
 eplo ! Plot elements to visualize the selection

 *get, edtol, elem, 0, count ! Get the total number of selected elements (edtol)
 *get, edmin, elem, 0, num, min ! Get the minimum element ID (edmin)
 ETABLE, ETAB_STRESS, s, eqv ! Define equivalent stress (von Mises stress) as a variable in the stress table

 ! Initialize equivalent stress array
 ESTRESS =
 *dim, ESTRESS,, edtol ! Define the dimensions of the ESTRESS array as the total number of selected elements
 *do, ie, edmin, edtol, 1 ! Loop through all elements (from edmin to edtol)
 *if, ESEL(ie), eq, 1, then ! If the element is selected
 *GET, von, ELEM, ie, ETAB, ETAB_STRESS ! Get the equivalent stress of the current element
 *if, von, gt, 2.1, then ! If the equivalent stress is greater than 2.1
 *if, von, lt, 2.2, then ! And less than 2.2
 ESTRESS(ie) = 1 ! Set the corresponding position in the ESTRESS array to 1 (meets condition)
 *endif
 *endif
 *endif
 *enddo

 ESEL, S, ELEM, , 1, 15712 ! Ensure only elements with IDs in the range 1 to 15712 are selected
 eplo ! Plot elements to visualize the selection
 ESEL, NONE ! Clear all selections
 *do, ie, edmin, edtol, 1 ! Loop through all elements again
 *if, ESTRESS(ie), eq, 1, then ! If the value in the ESTRESS array is 1 (element meets condition)
 ESEL, A,,, ie, ! Add the element to the selection
 *endif
 *enddo
 cm, ee3, elem ! Create a component named "ee3" to store the selected elements

 ! Calculate the total volume
 cmsel, s, ee3, elem ! Select the component "ee3" with the selected elements
 etable, evol, volu ! Define the volume variable
 ssum ! Sum the selected elements to calculate the total volume
 *get, VOLUME_TABLE(step), ssum,, item, evol ! Store the total volume in the VOLUME_TABLE array at the current step position
*enddo

! Output the volume table
*cfopen, volume_output22, txt ! Open a file to output the results
*do, step, 1, 40, 1
 *vwrite, step, VOLUME_TABLE(step)
 (F8.0, ', ', E15.6) ! Output format: first number is the step number, second number is the volume
*enddo
*cfclose

*dim, VOLUME_TABLE, array, 40 ! Create an array to store the volume for each load step, totaling 40 steps

/post1 ! Enter the post-processing module
*do, step, 1, 40, 1 ! Loop through each load step
 SET, step ! Set the current load step
 ALLSEL ! Select all elements and nodes

 ! Select elements with IDs in the range 1 to 15712
 ESEL, S, ELEM, , 1, 15712
 eplo ! Plot elements to visualize the selection

 *get, edtol, elem, 0, count ! Get the total number of selected elements (edtol)
 *get, edmin, elem, 0, num, min ! Get the minimum element ID (edmin)
 ETABLE, ETAB_STRESS, s, eqv ! Define equivalent stress (von Mises stress) as a variable in the stress table

 ! Initialize equivalent stress array
 ESTRESS =
 *dim, ESTRESS,, edtol ! Define the dimensions of the ESTRESS array as the total number of selected elements
 *do, ie, edmin, edtol, 1 ! Loop through all elements (from edmin to edtol)
 *if, ESEL(ie), eq, 1, then ! If the element is selected
 *GET, von, ELEM, ie, ETAB, ETAB_STRESS ! Get the equivalent stress of the current element
 *if, von, gt, 2.2, then ! If the equivalent stress is greater than 2.2
 *if, von, lt, 2.3, then ! And less than 2.3
 ESTRESS(ie) = 1 ! Set the corresponding position in the ESTRESS array to 1 (meets condition)
 *endif
 *endif
 *endif
 *enddo

 ESEL, S, ELEM, , 1, 15712 ! Ensure only elements with IDs in the range 1 to 15712 are selected
 eplo ! Plot elements to visualize the selection
 ESEL, NONE ! Clear all selections
 *do, ie, edmin, edtol, 1 ! Loop through all elements again
 *if, ESTRESS(ie), eq, 1, then ! If the value in the ESTRESS array is 1 (element meets condition)
 ESEL, A,,, ie, ! Add the element to the selection
 *endif
 *enddo
 cm, ee3, elem ! Create a component named "ee3" to store the selected elements

 ! Calculate the total volume
 cmsel, s, ee3, elem ! Select the component "ee3" with the selected elements
 etable, evol, volu ! Define the volume variable
 ssum ! Sum the selected elements to calculate the total volume
 *get, VOLUME_TABLE(step), ssum,, item, evol ! Store the total volume in the VOLUME_TABLE array at the current step position
*enddo

! Output the volume table
*cfopen, volume_output23, txt ! Open a file to output the results
*do, step, 1, 40, 1
 *vwrite, step, VOLUME_TABLE(step)
 (F8.0, ', ', E15.6) ! Output format: first number is the step number, second number is the volume
*enddo
*cfclose

*dim, VOLUME_TABLE, array, 40 ! Create an array to store the volume for each load step, totaling 40 steps

/post1 ! Enter the post-processing module
*do, step, 1, 40, 1 ! Loop through each load step
 SET, step ! Set the current load step
 ALLSEL ! Select all elements and nodes

 ! Select elements with IDs in the range 1 to 15712
 ESEL, S, ELEM, , 1, 15712
 eplo ! Plot elements to visualize the selection

 *get, edtol, elem, 0, count ! Get the total number of selected elements (edtol)
 *get, edmin, elem, 0, num, min ! Get the minimum element ID (edmin)
 ETABLE, ETAB_STRESS, s, eqv ! Define equivalent stress (von Mises stress) as a variable in the stress table

 ! Initialize equivalent stress array
 ESTRESS =
 *dim, ESTRESS,, edtol ! Define the dimensions of the ESTRESS array as the total number of selected elements
 *do, ie, edmin, edtol, 1 ! Loop through all elements (from edmin to edtol)
 *if, ESEL(ie), eq, 1, then ! If the element is selected
 *GET, von, ELEM, ie, ETAB, ETAB_STRESS ! Get the equivalent stress of the current element
 *if, von, gt, 2.3, then ! If the equivalent stress is greater than 2.3
 *if, von, lt, 2.4, then ! And less than 2.4
 ESTRESS(ie) = 1 ! Set the corresponding position in the ESTRESS array to 1 (meets condition)
 *endif
 *endif
 *endif
 *enddo

 ESEL, S, ELEM, , 1, 15712 ! Ensure only elements with IDs in the range 1 to 15712 are selected
 eplo ! Plot elements to visualize the selection
 ESEL, NONE ! Clear all selections
 *do, ie, edmin, edtol, 1 ! Loop through all elements again
 *if, ESTRESS(ie), eq, 1, then ! If the value in the ESTRESS array is 1 (element meets condition)
 ESEL, A,,, ie, ! Add the element to the selection
 *endif
 *enddo
 cm, ee3, elem ! Create a component named "ee3" to store the selected elements

 ! Calculate the total volume
 cmsel, s, ee3, elem ! Select the component "ee3" with the selected elements
 etable, evol, volu ! Define the volume variable
 ssum ! Sum the selected elements to calculate the total volume
 *get, VOLUME_TABLE(step), ssum,, item, evol ! Store the total volume in the VOLUME_TABLE array at the current step position
*enddo

! Output the volume table
*cfopen, volume_output24, txt ! Open a file to output the results
*do, step, 1, 40, 1
 *vwrite, step, VOLUME_TABLE(step)
 (F8.0, ', ', E15.6) ! Output format: first number is the step number, second number is the volume
*enddo
*cfclose

*dim, VOLUME_TABLE, array, 40 ! Create an array to store the volume for each load step, totaling 40 steps

/post1 ! Enter the post-processing module
*do, step, 1, 40, 1 ! Loop through each load step
 SET, step ! Set the current load step
 ALLSEL ! Select all elements and nodes

 ! Select elements with IDs in the range 1 to 15712
 ESEL, S, ELEM, , 1, 15712
 eplo ! Plot elements to visualize the selection

 *get, edtol, elem, 0, count ! Get the total number of selected elements (edtol)
 *get, edmin, elem, 0, num, min ! Get the minimum element ID (edmin)
 ETABLE, ETAB_STRESS, s, eqv ! Define equivalent stress (von Mises stress) as a variable in the stress table

 ! Initialize equivalent stress array
 ESTRESS =
 *dim, ESTRESS,, edtol ! Define the dimensions of the ESTRESS array as the total number of selected elements
 *do, ie, edmin, edtol, 1 ! Loop through all elements (from edmin to edtol)
 *if, ESEL(ie), eq, 1, then ! If the element is selected
 *GET, von, ELEM, ie, ETAB, ETAB_STRESS ! Get the equivalent stress of the current element
 *if, von, gt, 2.4, then ! If the equivalent stress is greater than 2.4
 *if, von, lt, 2.5, then ! And less than 2.5
 ESTRESS(ie) = 1 ! Set the corresponding position in the ESTRESS array to 1 (meets condition)
 *endif
 *endif
 *endif
 *enddo

 ESEL, S, ELEM, , 1, 15712 ! Ensure only elements with IDs in the range 1 to 15712 are selected
 eplo ! Plot elements to visualize the selection
 ESEL, NONE ! Clear all selections
 *do, ie, edmin, edtol, 1 ! Loop through all elements again
 *if, ESTRESS(ie), eq, 1, then ! If the value in the ESTRESS array is 1 (element meets condition)
 ESEL, A,,, ie, ! Add the element to the selection
 *endif
 *enddo
 cm, ee3, elem ! Create a component named "ee3" to store the selected elements

 ! Calculate the total volume
 cmsel, s, ee3, elem ! Select the component "ee3" with the selected elements
 etable, evol, volu ! Define the volume variable
 ssum ! Sum the selected elements to calculate the total volume
 *get, VOLUME_TABLE(step), ssum,, item, evol ! Store the total volume in the VOLUME_TABLE array at the current step position
*enddo

! Output the volume table
*cfopen, volume_output25, txt ! Open a file to output the results
*do, step, 1, 40, 1
 *vwrite, step, VOLUME_TABLE(step)
 (F8.0, ', ', E15.6) ! Output format: first number is the step number, second number is the volume
*enddo
*cfclose

*dim, VOLUME_TABLE, array, 40 ! Create an array to store the volume for each load step, totaling 40 steps

/post1 ! Enter the post-processing module
*do, step, 1, 40, 1 ! Loop through each load step
 SET, step ! Set the current load step
 ALLSEL ! Select all elements and nodes

 ! Select elements with IDs in the range 1 to 15712
 ESEL, S, ELEM, , 1, 15712
 eplo ! Plot elements to visualize the selection

 *get, edtol, elem, 0, count ! Get the total number of selected elements (edtol)
 *get, edmin, elem, 0, num, min ! Get the minimum element ID (edmin)
 ETABLE, ETAB_STRESS, s, eqv ! Define equivalent stress (von Mises stress) as a variable in the stress table

 ! Initialize equivalent stress array
 ESTRESS =
 *dim, ESTRESS,, edtol ! Define the dimensions of the ESTRESS array as the total number of selected elements
 *do, ie, edmin, edtol, 1 ! Loop through all elements (from edmin to edtol)
 *if, ESEL(ie), eq, 1, then ! If the element is selected
 *GET, von, ELEM, ie, ETAB, ETAB_STRESS ! Get the equivalent stress of the current element
 *if, von, gt, 2.5, then ! If the equivalent stress is greater than 2.5
 *if, von, lt, 2.6, then ! And less than 2.6
 ESTRESS(ie) = 1 ! Set the corresponding position in the ESTRESS array to 1 (meets condition)
 *endif
 *endif
 *endif
 *enddo

 ESEL, S, ELEM, , 1, 15712 ! Ensure only elements with IDs in the range 1 to 15712 are selected
 eplo ! Plot elements to visualize the selection
 ESEL, NONE ! Clear all selections
 *do, ie, edmin, edtol, 1 ! Loop through all elements again
 *if, ESTRESS(ie), eq, 1, then ! If the value in the ESTRESS array is 1 (element meets condition)
 ESEL, A,,, ie, ! Add the element to the selection
 *endif
 *enddo
 cm, ee3, elem ! Create a component named "ee3" to store the selected elements

 ! Calculate the total volume
 cmsel, s, ee3, elem ! Select the component "ee3" with the selected elements
 etable, evol, volu ! Define the volume variable
 ssum ! Sum the selected elements to calculate the total volume
 *get, VOLUME_TABLE(step), ssum,, item, evol ! Store the total volume in the VOLUME_TABLE array at the current step position
*enddo

! Output the volume table
*cfopen, volume_output26, txt ! Open a file to output the results
*do, step, 1, 40, 1
 *vwrite, step, VOLUME_TABLE(step)
 (F8.0, ', ', E15.6) ! Output format: first number is the step number, second number is the volume
*enddo
*cfclose

*dim, VOLUME_TABLE, array, 40 ! Create an array to store the volume for each load step, totaling 40 steps

/post1 ! Enter the post-processing module
*do, step, 1, 40, 1 ! Loop through each load step
 SET, step ! Set the current load step
 ALLSEL ! Select all elements and nodes

 ! Select elements with IDs in the range 1 to 15712
 ESEL, S, ELEM, , 1, 15712
 eplo ! Plot elements to visualize the selection

 *get, edtol, elem, 0, count ! Get the total number of selected elements (edtol)
 *get, edmin, elem, 0, num, min ! Get the minimum element ID (edmin)
 ETABLE, ETAB_STRESS, s, eqv ! Define equivalent stress (von Mises stress) as a variable in the stress table

 ! Initialize equivalent stress array
 ESTRESS =
 *dim, ESTRESS,, edtol ! Define the dimensions of the ESTRESS array as the total number of selected elements
 *do, ie, edmin, edtol, 1 ! Loop through all elements (from edmin to edtol)
 *if, ESEL(ie), eq, 1, then ! If the element is selected
 *GET, von, ELEM, ie, ETAB, ETAB_STRESS ! Get the equivalent stress of the current element
 *if, von, gt, 2.6, then ! If the equivalent stress is greater than 2.6
 *if, von, lt, 2.7, then ! And less than 2.7
 ESTRESS(ie) = 1 ! Set the corresponding position in the ESTRESS array to 1 (meets condition)
 *endif
 *endif
 *endif
 *enddo

 ESEL, S, ELEM, , 1, 15712 ! Ensure only elements with IDs in the range 1 to 15712 are selected
 eplo ! Plot elements to visualize the selection
 ESEL, NONE ! Clear all selections
 *do, ie, edmin, edtol, 1 ! Loop through all elements again
 *if, ESTRESS(ie), eq, 1, then ! If the value in the ESTRESS array is 1 (element meets condition)
 ESEL, A,,, ie, ! Add the element to the selection
 *endif
 *enddo
 cm, ee3, elem ! Create a component named "ee3" to store the selected elements

 ! Calculate the total volume
 cmsel, s, ee3, elem ! Select the component "ee3" with the selected elements
 etable, evol, volu ! Define the volume variable
 ssum ! Sum the selected elements to calculate the total volume
 *get, VOLUME_TABLE(step), ssum,, item, evol ! Store the total volume in the VOLUME_TABLE array at the current step position
*enddo

! Output the volume table
*cfopen, volume_output27, txt ! Open a file to output the results
*do, step, 1, 40, 1
 *vwrite, step, VOLUME_TABLE(step)
 (F8.0, ', ', E15.6) ! Output format: first number is the step number, second number is the volume
*enddo
*cfclose

*dim, VOLUME_TABLE, array, 40 ! Create an array to store the volume for each load step, totaling 40 steps

/post1 ! Enter the post-processing module
*do, step, 1, 40, 1 ! Loop through each load step
 SET, step ! Set the current load step
 ALLSEL ! Select all elements and nodes

 ! Select elements with IDs in the range 1 to 15712
 ESEL, S, ELEM, , 1, 15712
 eplo ! Plot elements to visualize the selection

 *get, edtol, elem, 0, count ! Get the total number of selected elements (edtol)
 *get, edmin, elem, 0, num, min ! Get the minimum element ID (edmin)
 ETABLE, ETAB_STRESS, s, eqv ! Define equivalent stress (von Mises stress) as a variable in the stress table

 ! Initialize equivalent stress array
 ESTRESS =
 *dim, ESTRESS,, edtol ! Define the dimensions of the ESTRESS array as the total number of selected elements
 *do, ie, edmin, edtol, 1 ! Loop through all elements (from edmin to edtol)
 *if, ESEL(ie), eq, 1, then ! If the element is selected
 *GET, von, ELEM, ie, ETAB, ETAB_STRESS ! Get the equivalent stress of the current element
 *if, von, gt, 2.7, then ! If the equivalent stress is greater than 2.7
 *if, von, lt, 2.8, then ! And less than 2.8
 ESTRESS(ie) = 1 ! Set the corresponding position in the ESTRESS array to 1 (meets condition)
 *endif
 *endif
 *endif
 *enddo

 ESEL, S, ELEM, , 1, 15712 ! Ensure only elements with IDs in the range 1 to 15712 are selected
 eplo ! Plot elements to visualize the selection
 ESEL, NONE ! Clear all selections
 *do, ie, edmin, edtol, 1 ! Loop through all elements again
 *if, ESTRESS(ie), eq, 1, then ! If the value in the ESTRESS array is 1 (element meets condition)
 ESEL, A,,, ie, ! Add the element to the selection
 *endif
 *enddo
 cm, ee3, elem ! Create a component named "ee3" to store the selected elements

 ! Calculate the total volume
 cmsel, s, ee3, elem ! Select the component "ee3" with the selected elements
 etable, evol, volu ! Define the volume variable
 ssum ! Sum the selected elements to calculate the total volume
 *get, VOLUME_TABLE(step), ssum,, item, evol ! Store the total volume in the VOLUME_TABLE array at the current step position
*enddo

! Output the volume table
*cfopen, volume_output28, txt ! Open a file to output the results
*do, step, 1, 40, 1
 *vwrite, step, VOLUME_TABLE(step)
 (F8.0, ', ', E15.6) ! Output format: first number is the step number, second number is the volume
*enddo
*cfclose

*dim, VOLUME_TABLE, array, 40 ! Create an array to store the volume for each load step, totaling 40 steps

/post1 ! Enter the post-processing module
*do, step, 1, 40, 1 ! Loop through each load step
 SET, step ! Set the current load step
 ALLSEL ! Select all elements and nodes

 ! Select elements with IDs in the range 1 to 15712
 ESEL, S, ELEM, , 1, 15712
 eplo ! Plot elements to visualize the selection

 *get, edtol, elem, 0, count ! Get the total number of selected elements (edtol)
 *get, edmin, elem, 0, num, min ! Get the minimum element ID (edmin)
 ETABLE, ETAB_STRESS, s, eqv ! Define equivalent stress (von Mises stress) as a variable in the stress table

 ! Initialize equivalent stress array
 ESTRESS =
 *dim, ESTRESS,, edtol ! Define the dimensions of the ESTRESS array as the total number of selected elements
 *do, ie, edmin, edtol, 1 ! Loop through all elements (from edmin to edtol)
 *if, ESEL(ie), eq, 1, then ! If the element is selected
 *GET, von, ELEM, ie, ETAB, ETAB_STRESS ! Get the equivalent stress of the current element
 *if, von, gt, 2.8, then ! If the equivalent stress is greater than 2.8
 *if, von, lt, 2.9, then ! And less than 2.9
 ESTRESS(ie) = 1 ! Set the corresponding position in the ESTRESS array to 1 (meets condition)
 *endif
 *endif
 *endif
 *enddo

 ESEL, S, ELEM, , 1, 15712 ! Ensure only elements with IDs in the range 1 to 15712 are selected
 eplo ! Plot elements to visualize the selection
 ESEL, NONE ! Clear all selections
 *do, ie, edmin, edtol, 1 ! Loop through all elements again
 *if, ESTRESS(ie), eq, 1, then ! If the value in the ESTRESS array is 1 (element meets condition)
 ESEL, A,,, ie, ! Add the element to the selection
 *endif
 *enddo
 cm, ee3, elem ! Create a component named "ee3" to store the selected elements

 ! Calculate the total volume
 cmsel, s, ee3, elem ! Select the component "ee3" with the selected elements
 etable, evol, volu ! Define the volume variable
 ssum ! Sum the selected elements to calculate the total volume
 *get, VOLUME_TABLE(step), ssum,, item, evol ! Store the total volume in the VOLUME_TABLE array at the current step position
*enddo

! Output the volume table
*cfopen, volume_output29, txt ! Open a file to output the results
*do, step, 1, 40, 1
 *vwrite, step, VOLUME_TABLE(step)
 (F8.0, ', ', E15.6) ! Output format: first number is the step number, second number is the volume
*enddo
*cfclose

*dim, VOLUME_TABLE, array, 40 ! Create an array to store the volume for each load step, totaling 40 steps

/post1 ! Enter the post-processing module
*do, step, 1, 40, 1 ! Loop through each load step
 SET, step ! Set the current load step
 ALLSEL ! Select all elements and nodes

 ! Select elements with IDs in the range 1 to 15712
 ESEL, S, ELEM, , 1, 15712
 eplo ! Plot elements to visualize the selection

 *get, edtol, elem, 0, count ! Get the total number of selected elements (edtol)
 *get, edmin, elem, 0, num, min ! Get the minimum element ID (edmin)
 ETABLE, ETAB_STRESS, s, eqv ! Define equivalent stress (von Mises stress) as a variable in the stress table

 ! Initialize equivalent stress array
 ESTRESS =
 *dim, ESTRESS,, edtol ! Define the dimensions of the ESTRESS array as the total number of selected elements
 *do, ie, edmin, edtol, 1 ! Loop through all elements (from edmin to edtol)
 *if, ESEL(ie), eq, 1, then ! If the element is selected
 *GET, von, ELEM, ie, ETAB, ETAB_STRESS ! Get the equivalent stress of the current element
 *if, von, gt, 2.9, then ! If the equivalent stress is greater than 2.9
 *if, von, lt, 3.0, then ! And less than 3.0
 ESTRESS(ie) = 1 ! Set the corresponding position in the ESTRESS array to 1 (meets condition)
 *endif
 *endif
 *endif
 *enddo

 ESEL, S, ELEM, , 1, 15712 ! Ensure only elements with IDs in the range 1 to 15712 are selected
 eplo ! Plot elements to visualize the selection
 ESEL, NONE ! Clear all selections
 *do, ie, edmin, edtol, 1 ! Loop through all elements again
 *if, ESTRESS(ie), eq, 1, then ! If the value in the ESTRESS array is 1 (element meets condition)
 ESEL, A,,, ie, ! Add the element to the selection
 *endif
 *enddo
 cm, ee3, elem ! Create a component named "ee3" to store the selected elements

 ! Calculate the total volume
 cmsel, s, ee3, elem ! Select the component "ee3" with the selected elements
 etable, evol, volu ! Define the volume variable
 ssum ! Sum the selected elements to calculate the total volume
 *get, VOLUME_TABLE(step), ssum,, item, evol ! Store the total volume in the VOLUME_TABLE array at the current step position
*enddo

! Output the volume table
*cfopen, volume_output30, txt ! Open a file to output the results
*do, step, 1, 40, 1
 *vwrite, step, VOLUME_TABLE(step)
 (F8.0, ', ', E15.6) ! Output format: first number is the step number, second number is the volume
*enddo
*cfclose

*dim, VOLUME_TABLE, array, 40 ! Create an array to store the volume for each load step, totaling 40 steps

/post1 ! Enter the post-processing module
*do, step, 1, 40, 1 ! Loop through each load step
 SET, step ! Set the current load step
 ALLSEL ! Select all elements and nodes

 ! Select elements with IDs in the range 1 to 15712
 ESEL, S, ELEM, , 1, 15712
 eplo ! Plot elements to visualize the selection

 *get, edtol, elem, 0, count ! Get the total number of selected elements (edtol)
 *get, edmin, elem, 0, num, min ! Get the minimum element ID (edmin)
 ETABLE, ETAB_STRESS, s, eqv ! Define equivalent stress (von Mises stress) as a variable in the stress table

 ! Initialize equivalent stress array
 ESTRESS =
 *dim, ESTRESS,, edtol ! Define the dimensions of the ESTRESS array as the total number of selected elements
 *do, ie, edmin, edtol, 1 ! Loop through all elements (from edmin to edtol)
 *if, ESEL(ie), eq, 1, then ! If the element is selected
 *GET, von, ELEM, ie, ETAB, ETAB_STRESS ! Get the equivalent stress of the current element
 *if, von, gt, 3.0, then ! If the equivalent stress is greater than 3.0
 *if, von, lt, 3.1, then ! And less than 3.1
 ESTRESS(ie) = 1 ! Set the corresponding position in the ESTRESS array to 1 (meets condition)
 *endif
 *endif
 *endif
 *enddo

 ESEL, S, ELEM, , 1, 15712 ! Ensure only elements with IDs in the range 1 to 15712 are selected
 eplo ! Plot elements to visualize the selection
 ESEL, NONE ! Clear all selections
 *do, ie, edmin, edtol, 1 ! Loop through all elements again
 *if, ESTRESS(ie), eq, 1, then ! If the value in the ESTRESS array is 1 (element meets condition)
 ESEL, A,,, ie, ! Add the element to the selection
 *endif
 *enddo
 cm, ee3, elem ! Create a component named "ee3" to store the selected elements

 ! Calculate the total volume
 cmsel, s, ee3, elem ! Select the component "ee3" with the selected elements
 etable, evol, volu ! Define the volume variable
 ssum ! Sum the selected elements to calculate the total volume
 *get, VOLUME_TABLE(step), ssum,, item, evol ! Store the total volume in the VOLUME_TABLE array at the current step position
*enddo

! Output the volume table
*cfopen, volume_output31, txt ! Open a file to output the results
*do, step, 1, 40, 1
 *vwrite, step, VOLUME_TABLE(step)
 (F8.0, ', ', E15.6) ! Output format: first number is the step number, second number is the volume
*enddo
*cfclose

*dim, VOLUME_TABLE, array, 40 ! Create an array to store the volume for each load step, totaling 40 steps

/post1 ! Enter the post-processing module
*do, step, 1, 40, 1 ! Loop through each load step
 SET, step ! Set the current load step
 ALLSEL ! Select all elements and nodes

 ! Select elements with IDs in the range 1 to 15712
 ESEL, S, ELEM, , 1, 15712
 eplo ! Plot elements to visualize the selection

 *get, edtol, elem, 0, count ! Get the total number of selected elements (edtol)
 *get, edmin, elem, 0, num, min ! Get the minimum element ID (edmin)
 ETABLE, ETAB_STRESS, s, eqv ! Define equivalent stress (von Mises stress) as a variable in the stress table

 ! Initialize equivalent stress array
 ESTRESS =
 *dim, ESTRESS,, edtol ! Define the dimensions of the ESTRESS array as the total number of selected elements
 *do, ie, edmin, edtol, 1 ! Loop through all elements (from edmin to edtol)
 *if, ESEL(ie), eq, 1, then ! If the element is selected
 *GET, von, ELEM, ie, ETAB, ETAB_STRESS ! Get the equivalent stress of the current element
 *if, von, gt, 3.1, then ! If the equivalent stress is greater than 3.1
 *if, von, lt, 3.2, then ! And less than 3.2
 ESTRESS(ie) = 1 ! Set the corresponding position in the ESTRESS array to 1 (meets condition)
 *endif
 *endif
 *endif
 *enddo

 ESEL, S, ELEM, , 1, 15712 ! Ensure only elements with IDs in the range 1 to 15712 are selected
 eplo ! Plot elements to visualize the selection
 ESEL, NONE ! Clear all selections
 *do, ie, edmin, edtol, 1 ! Loop through all elements again
 *if, ESTRESS(ie), eq, 1, then ! If the value in the ESTRESS array is 1 (element meets condition)
 ESEL, A,,, ie, ! Add the element to the selection
 *endif
 *enddo
 cm, ee3, elem ! Create a component named "ee3" to store the selected elements

 ! Calculate the total volume
 cmsel, s, ee3, elem ! Select the component "ee3" with the selected elements
 etable, evol, volu ! Define the volume variable
 ssum ! Sum the selected elements to calculate the total volume
 *get, VOLUME_TABLE(step), ssum,, item, evol ! Store the total volume in the VOLUME_TABLE array at the current step position
*enddo

! Output the volume table
*cfopen, volume_output32, txt ! Open a file to output the results
*do, step, 1, 40, 1
 *vwrite, step, VOLUME_TABLE(step)
 (F8.0, ', ', E15.6) ! Output format: first number is the step number, second number is the volume
*enddo
*cfclose

*dim, VOLUME_TABLE, array, 40 ! Create an array to store the volume for each load step, totaling 40 steps

/post1 ! Enter the post-processing module
*do, step, 1, 40, 1 ! Loop through each load step
 SET, step ! Set the current load step
 ALLSEL ! Select all elements and nodes

 ! Select elements with IDs in the range 1 to 15712
 ESEL, S, ELEM, , 1, 15712
 eplo ! Plot elements to visualize the selection

 *get, edtol, elem, 0, count ! Get the total number of selected elements (edtol)
 *get, edmin, elem, 0, num, min ! Get the minimum element ID (edmin)
 ETABLE, ETAB_STRESS, s, eqv ! Define equivalent stress (von Mises stress) as a variable in the stress table

 ! Initialize equivalent stress array
 ESTRESS =
 *dim, ESTRESS,, edtol ! Define the dimensions of the ESTRESS array as the total number of selected elements
 *do, ie, edmin, edtol, 1 ! Loop through all elements (from edmin to edtol)
 *if, ESEL(ie), eq, 1, then ! If the element is selected
 *GET, von, ELEM, ie, ETAB, ETAB_STRESS ! Get the equivalent stress of the current element
 *if, von, gt, 3.2, then ! If the equivalent stress is greater than 3.2
 *if, von, lt, 3.3, then ! And less than 3.3
 ESTRESS(ie) = 1 ! Set the corresponding position in the ESTRESS array to 1 (meets condition)
 *endif
 *endif
 *endif
 *enddo

 ESEL, S, ELEM, , 1, 15712 ! Ensure only elements with IDs in the range 1 to 15712 are selected
 eplo ! Plot elements to visualize the selection
 ESEL, NONE ! Clear all selections
 *do, ie, edmin, edtol, 1 ! Loop through all elements again
 *if, ESTRESS(ie), eq, 1, then ! If the value in the ESTRESS array is 1 (element meets condition)
 ESEL, A,,, ie, ! Add the element to the selection
 *endif
 *enddo
 cm, ee3, elem ! Create a component named "ee3" to store the selected elements

 ! Calculate the total volume
 cmsel, s, ee3, elem ! Select the component "ee3" with the selected elements
 etable, evol, volu ! Define the volume variable
 ssum ! Sum the selected elements to calculate the total volume
 *get, VOLUME_TABLE(step), ssum,, item, evol ! Store the total volume in the VOLUME_TABLE array at the current step position
*enddo

! Output the volume table
*cfopen, volume_output33, txt ! Open a file to output the results
*do, step, 1, 40, 1
 *vwrite, step, VOLUME_TABLE(step)
 (F8.0, ', ', E15.6) ! Output format: first number is the step number, second number is the volume
*enddo
*cfclose

*dim, VOLUME_TABLE, array, 40 ! Create an array to store the volume for each load step, totaling 40 steps

/post1 ! Enter the post-processing module
*do, step, 1, 40, 1 ! Loop through each load step
 SET, step ! Set the current load step
 ALLSEL ! Select all elements and nodes

 ! Select elements with IDs in the range 1 to 15712
 ESEL, S, ELEM, , 1, 15712
 eplo ! Plot elements to visualize the selection

 *get, edtol, elem, 0, count ! Get the total number of selected elements (edtol)
 *get, edmin, elem, 0, num, min ! Get the minimum element ID (edmin)
 ETABLE, ETAB_STRESS, s, eqv ! Define equivalent stress (von Mises stress) as a variable in the stress table

 ! Initialize equivalent stress array
 ESTRESS =
 *dim, ESTRESS,, edtol ! Define the dimensions of the ESTRESS array as the total number of selected elements
 *do, ie, edmin, edtol, 1 ! Loop through all elements (from edmin to edtol)
 *if, ESEL(ie), eq, 1, then ! If the element is selected
 *GET, von, ELEM, ie, ETAB, ETAB_STRESS ! Get the equivalent stress of the current element
 *if, von, gt, 3.3, then ! If the equivalent stress is greater than 3.3
 *if, von, lt, 3.4, then ! And less than 3.4
 ESTRESS(ie) = 1 ! Set the corresponding position in the ESTRESS array to 1 (meets condition)
 *endif
 *endif
 *endif
 *enddo

 ESEL, S, ELEM, , 1, 15712 ! Ensure only elements with IDs in the range 1 to 15712 are selected
 eplo ! Plot elements to visualize the selection
 ESEL, NONE ! Clear all selections
 *do, ie, edmin, edtol, 1 ! Loop through all elements again
 *if, ESTRESS(ie), eq, 1, then ! If the value in the ESTRESS array is 1 (element meets condition)
 ESEL, A,,, ie, ! Add the element to the selection
 *endif
 *enddo
 cm, ee3, elem ! Create a component named "ee3" to store the selected elements

 ! Calculate the total volume
 cmsel, s, ee3, elem ! Select the component "ee3" with the selected elements
 etable, evol, volu ! Define the volume variable
 ssum ! Sum the selected elements to calculate the total volume
 *get, VOLUME_TABLE(step), ssum,, item, evol ! Store the total volume in the VOLUME_TABLE array at the current step position
*enddo

! Output the volume table
*cfopen, volume_output34, txt ! Open a file to output the results
*do, step, 1, 40, 1
 *vwrite, step, VOLUME_TABLE(step)
 (F8.0, ', ', E15.6) ! Output format: first number is the step number, second number is the volume
*enddo
*cfclose

*dim, VOLUME_TABLE, array, 40 ! Create an array to store the volume for each load step, totaling 40 steps

/post1 ! Enter the post-processing module
*do, step, 1, 40, 1 ! Loop through each load step
 SET, step ! Set the current load step
 ALLSEL ! Select all elements and nodes

 ! Select elements with IDs in the range 1 to 15712
 ESEL, S, ELEM, , 1, 15712
 eplo ! Plot elements to visualize the selection

 *get, edtol, elem, 0, count ! Get the total number of selected elements (edtol)
 *get, edmin, elem, 0, num, min ! Get the minimum element ID (edmin)
 ETABLE, ETAB_STRESS, s, eqv ! Define equivalent stress (von Mises stress) as a variable in the stress table

 ! Initialize equivalent stress array
 ESTRESS =
 *dim, ESTRESS,, edtol ! Define the dimensions of the ESTRESS array as the total number of selected elements
 *do, ie, edmin, edtol, 1 ! Loop through all elements (from edmin to edtol)
 *if, ESEL(ie), eq, 1, then ! If the element is selected
 *GET, von, ELEM, ie, ETAB, ETAB_STRESS ! Get the equivalent stress of the current element
 *if, von, gt, 3.4, then ! If the equivalent stress is greater than 3.4
 *if, von, lt, 3.5, then ! And less than 3.5
 ESTRESS(ie) = 1 ! Set the corresponding position in the ESTRESS array to 1 (meets condition)
 *endif
 *endif
 *endif
 *enddo

 ESEL, S, ELEM, , 1, 15712 ! Ensure only elements with IDs in the range 1 to 15712 are selected
 eplo ! Plot elements to visualize the selection
 ESEL, NONE ! Clear all selections
 *do, ie, edmin, edtol, 1 ! Loop through all elements again
 *if, ESTRESS(ie), eq, 1, then ! If the value in the ESTRESS array is 1 (element meets condition)
 ESEL, A,,, ie, ! Add the element to the selection
 *endif
 *enddo
 cm, ee3, elem ! Create a component named "ee3" to store the selected elements

 ! Calculate the total volume
 cmsel, s, ee3, elem ! Select the component "ee3" with the selected elements
 etable, evol, volu ! Define the volume variable
 ssum ! Sum the selected elements to calculate the total volume
 *get, VOLUME_TABLE(step), ssum,, item, evol ! Store the total volume in the VOLUME_TABLE array at the current step position
*enddo

! Output the volume table
*cfopen, volume_output35, txt ! Open a file to output the results
*do, step, 1, 40, 1
 *vwrite, step, VOLUME_TABLE(step)
 (F8.0, ', ', E15.6) ! Output format: first number is the step number, second number is the volume
*enddo
*cfclose

*dim, VOLUME_TABLE, array, 40 ! Create an array to store the volume for each load step, totaling 40 steps

/post1 ! Enter the post-processing module
*do, step, 1, 40, 1 ! Loop through each load step
 SET, step ! Set the current load step
 ALLSEL ! Select all elements and nodes

 ! Select elements with IDs in the range 1 to 15712
 ESEL, S, ELEM, , 1, 15712
 eplo ! Plot elements to visualize the selection

 *get, edtol, elem, 0, count ! Get the total number of selected elements (edtol)
 *get, edmin, elem, 0, num, min ! Get the minimum element ID (edmin)
 ETABLE, ETAB_STRESS, s, eqv ! Define equivalent stress (von Mises stress) as a variable in the stress table

 ! Initialize equivalent stress array
 ESTRESS =
 *dim, ESTRESS,, edtol ! Define the dimensions of the ESTRESS array as the total number of selected elements
 *do, ie, edmin, edtol, 1 ! Loop through all elements (from edmin to edtol)
 *if, ESEL(ie), eq, 1, then ! If the element is selected
 *GET, von, ELEM, ie, ETAB, ETAB_STRESS ! Get the equivalent stress of the current element
 *if, von, gt, 3.5, then ! If the equivalent stress is greater than 3.5
 *if, von, lt, 3.6, then ! And less than 3.6
 ESTRESS(ie) = 1 ! Set the corresponding position in the ESTRESS array to 1 (meets condition)
 *endif
 *endif
 *endif
 *enddo

 ESEL, S, ELEM, , 1, 15712 ! Ensure only elements with IDs in the range 1 to 15712 are selected
 eplo ! Plot elements to visualize the selection
 ESEL, NONE ! Clear all selections
 *do, ie, edmin, edtol, 1 ! Loop through all elements again
 *if, ESTRESS(ie), eq, 1, then ! If the value in the ESTRESS array is 1 (element meets condition)
 ESEL, A,,, ie, ! Add the element to the selection
 *endif
 *enddo
 cm, ee3, elem ! Create a component named "ee3" to store the selected elements

 ! Calculate the total volume
 cmsel, s, ee3, elem ! Select the component "ee3" with the selected elements
 etable, evol, volu ! Define the volume variable
 ssum ! Sum the selected elements to calculate the total volume
 *get, VOLUME_TABLE(step), ssum,, item, evol ! Store the total volume in the VOLUME_TABLE array at the current step position
*enddo

! Output the volume table
*cfopen, volume_output36, txt ! Open a file to output the results
*do, step, 1, 40, 1
 *vwrite, step, VOLUME_TABLE(step)
 (F8.0, ', ', E15.6) ! Output format: first number is the step number, second number is the volume
*enddo
*cfclose

*dim, VOLUME_TABLE, array, 40 ! Create an array to store the volume for each load step, totaling 40 steps

/post1 ! Enter the post-processing module
*do, step, 1, 40, 1 ! Loop through each load step
 SET, step ! Set the current load step
 ALLSEL ! Select all elements and nodes

 ! Select elements with IDs in the range 1 to 15712
 ESEL, S, ELEM, , 1, 15712
 eplo ! Plot elements to visualize the selection

 *get, edtol, elem, 0, count ! Get the total number of selected elements (edtol)
 *get, edmin, elem, 0, num, min ! Get the minimum element ID (edmin)
 ETABLE, ETAB_STRESS, s, eqv ! Define equivalent stress (von Mises stress) as a variable in the stress table

 ! Initialize equivalent stress array
 ESTRESS =
 *dim, ESTRESS,, edtol ! Define the dimensions of the ESTRESS array as the total number of selected elements
 *do, ie, edmin, edtol, 1 ! Loop through all elements (from edmin to edtol)
 *if, ESEL(ie), eq, 1, then ! If the element is selected
 *GET, von, ELEM, ie, ETAB, ETAB_STRESS ! Get the equivalent stress of the current element
 *if, von, gt, 3.6, then ! If the equivalent stress is greater than 3.6
 *if, von, lt, 3.7, then ! And less than 3.7
 ESTRESS(ie) = 1 ! Set the corresponding position in the ESTRESS array to 1 (meets condition)
 *endif
 *endif
 *endif
 *enddo

 ESEL, S, ELEM, , 1, 15712 ! Ensure only elements with IDs in the range 1 to 15712 are selected
 eplo ! Plot elements to visualize the selection
 ESEL, NONE ! Clear all selections
 *do, ie, edmin, edtol, 1 ! Loop through all elements again
 *if, ESTRESS(ie), eq, 1, then ! If the value in the ESTRESS array is 1 (element meets condition)
 ESEL, A,,, ie, ! Add the element to the selection
 *endif
 *enddo
 cm, ee3, elem ! Create a component named "ee3" to store the selected elements

 ! Calculate the total volume
 cmsel, s, ee3, elem ! Select the component "ee3" with the selected elements
 etable, evol, volu ! Define the volume variable
 ssum ! Sum the selected elements to calculate the total volume
 *get, VOLUME_TABLE(step), ssum,, item, evol ! Store the total volume in the VOLUME_TABLE array at the current step position
*enddo

! Output the volume table
*cfopen, volume_output37, txt ! Open a file to output the results
*do, step, 1, 40, 1
 *vwrite, step, VOLUME_TABLE(step)
 (F8.0, ', ', E15.6) ! Output format: first number is the step number, second number is the volume
*enddo
*cfclose

*dim, VOLUME_TABLE, array, 40 ! Create an array to store the volume for each load step, totaling 40 steps

/post1 ! Enter the post-processing module
*do, step, 1, 40, 1 ! Loop through each load step
 SET, step ! Set the current load step
 ALLSEL ! Select all elements and nodes

 ! Select elements with IDs in the range 1 to 15712
 ESEL, S, ELEM, , 1, 15712
 eplo ! Plot elements to visualize the selection

 *get, edtol, elem, 0, count ! Get the total number of selected elements (edtol)
 *get, edmin, elem, 0, num, min ! Get the minimum element ID (edmin)
 ETABLE, ETAB_STRESS, s, eqv ! Define equivalent stress (von Mises stress) as a variable in the stress table

 ! Initialize equivalent stress array
 ESTRESS =
 *dim, ESTRESS,, edtol ! Define the dimensions of the ESTRESS array as the total number of selected elements
 *do, ie, edmin, edtol, 1 ! Loop through all elements (from edmin to edtol)
 *if, ESEL(ie), eq, 1, then ! If the element is selected
 *GET, von, ELEM, ie, ETAB, ETAB_STRESS ! Get the equivalent stress of the current element
 *if, von, gt, 3.7, then ! If the equivalent stress is greater than 3.7
 *if, von, lt, 3.8, then ! And less than 3.8
 ESTRESS(ie) = 1 ! Set the corresponding position in the ESTRESS array to 1 (meets condition)
 *endif
 *endif
 *endif
 *enddo

 ESEL, S, ELEM, , 1, 15712 ! Ensure only elements with IDs in the range 1 to 15712 are selected
 eplo ! Plot elements to visualize the selection
 ESEL, NONE ! Clear all selections
 *do, ie, edmin, edtol, 1 ! Loop through all elements again
 *if, ESTRESS(ie), eq, 1, then ! If the value in the ESTRESS array is 1 (element meets condition)
 ESEL, A,,, ie, ! Add the element to the selection
 *endif
 *enddo
 cm, ee3, elem ! Create a component named "ee3" to store the selected elements

 ! Calculate the total volume
 cmsel, s, ee3, elem ! Select the component "ee3" with the selected elements
 etable, evol, volu ! Define the volume variable
 ssum ! Sum the selected elements to calculate the total volume
 *get, VOLUME_TABLE(step), ssum,, item, evol ! Store the total volume in the VOLUME_TABLE array at the current step position
*enddo

! Output the volume table
*cfopen, volume_output38, txt ! Open a file to output the results
*do, step, 1, 40, 1
 *vwrite, step, VOLUME_TABLE(step)
 (F8.0, ', ', E15.6) ! Output format: first number is the step number, second number is the volume
*enddo
*cfclose

*dim, VOLUME_TABLE, array, 40 ! Create an array to store the volume for each load step, totaling 40 steps

/post1 ! Enter the post-processing module
*do, step, 1, 40, 1 ! Loop through each load step
 SET, step ! Set the current load step
 ALLSEL ! Select all elements and nodes

 ! Select elements with IDs in the range 1 to 15712
 ESEL, S, ELEM, , 1, 15712
 eplo ! Plot elements to visualize the selection

 *get, edtol, elem, 0, count ! Get the total number of selected elements (edtol)
 *get, edmin, elem, 0, num, min ! Get the minimum element ID (edmin)
 ETABLE, ETAB_STRESS, s, eqv ! Define equivalent stress (von Mises stress) as a variable in the stress table

 ! Initialize equivalent stress array
 ESTRESS =
 *dim, ESTRESS,, edtol ! Define the dimensions of the ESTRESS array as the total number of selected elements
 *do, ie, edmin, edtol, 1 ! Loop through all elements (from edmin to edtol)
 *if, ESEL(ie), eq, 1, then ! If the element is selected
 *GET, von, ELEM, ie, ETAB, ETAB_STRESS ! Get the equivalent stress of the current element
 *if, von, gt, 3.8, then ! If the equivalent stress is greater than 3.8
 *if, von, lt, 3.9, then ! And less than 3.9
 ESTRESS(ie) = 1 ! Set the corresponding position in the ESTRESS array to 1 (meets condition)
 *endif
 *endif
 *endif
 *enddo

 ESEL, S, ELEM, , 1, 15712 ! Ensure only elements with IDs in the range 1 to 15712 are selected
 eplo ! Plot elements to visualize the selection
 ESEL, NONE ! Clear all selections
 *do, ie, edmin, edtol, 1 ! Loop through all elements again
 *if, ESTRESS(ie), eq, 1, then ! If the value in the ESTRESS array is 1 (element meets condition)
 ESEL, A,,, ie, ! Add the element to the selection
 *endif
 *enddo
 cm, ee3, elem ! Create a component named "ee3" to store the selected elements

 ! Calculate the total volume
 cmsel, s, ee3, elem ! Select the component "ee3" with the selected elements
 etable, evol, volu ! Define the volume variable
 ssum ! Sum the selected elements to calculate the total volume
 *get, VOLUME_TABLE(step), ssum,, item, evol ! Store the total volume in the VOLUME_TABLE array at the current step position
*enddo

! Output the volume table
*cfopen, volume_output39, txt ! Open a file to output the results
*do, step, 1, 40, 1
 *vwrite, step, VOLUME_TABLE(step)
 (F8.0, ', ', E15.6) ! Output format: first number is the step number, second number is the volume
*enddo
*cfclose

*dim, VOLUME_TABLE, array, 40 ! Create an array to store the volume for each load step, totaling 40 steps

/post1 ! Enter the post-processing module
*do, step, 1, 40, 1 ! Loop through each load step
 SET, step ! Set the current load step
 ALLSEL ! Select all elements and nodes

 ! Select elements with IDs in the range 1 to 15712
 ESEL, S, ELEM, , 1, 15712
 eplo ! Plot elements to visualize the selection

 *get, edtol, elem, 0, count ! Get the total number of selected elements (edtol)
 *get, edmin, elem, 0, num, min ! Get the minimum element ID (edmin)
 ETABLE, ETAB_STRESS, s, eqv ! Define equivalent stress (von Mises stress) as a variable in the stress table

 ! Initialize equivalent stress array
 ESTRESS =
 *dim, ESTRESS,, edtol ! Define the dimensions of the ESTRESS array as the total number of selected elements
 *do, ie, edmin, edtol, 1 ! Loop through all elements (from edmin to edtol)
 *if, ESEL(ie), eq, 1, then ! If the element is selected
 *GET, von, ELEM, ie, ETAB, ETAB_STRESS ! Get the equivalent stress of the current element
 *if, von, gt, 3.9, then ! If the equivalent stress is greater than 3.9
 *if, von, lt, 4.0, then ! And less than 4.0
 ESTRESS(ie) = 1 ! Set the corresponding position in the ESTRESS array to 1 (meets condition)
 *endif
 *endif
 *endif
 *enddo

 ESEL, S, ELEM, , 1, 15712 ! Ensure only elements with IDs in the range 1 to 15712 are selected
 eplo ! Plot elements to visualize the selection
 ESEL, NONE ! Clear all selections
 *do, ie, edmin, edtol, 1 ! Loop through all elements again
 *if, ESTRESS(ie), eq, 1, then ! If the value in the ESTRESS array is 1 (element meets condition)
 ESEL, A,,, ie, ! Add the element to the selection
 *endif
 *enddo
 cm, ee3, elem ! Create a component named "ee3" to store the selected elements

 ! Calculate the total volume
 cmsel, s, ee3, elem ! Select the component "ee3" with the selected elements
 etable, evol, volu ! Define the volume variable
 ssum ! Sum the selected elements to calculate the total volume
 *get, VOLUME_TABLE(step), ssum,, item, evol ! Store the total volume in the VOLUME_TABLE array at the current step position
*enddo

! Output the volume table
*cfopen, volume_output40, txt ! Open a file to output the results
*do, step, 1, 40, 1
 *vwrite, step, VOLUME_TABLE(step)
 (F8.0, ', ', E15.6) ! Output format: first number is the step number, second number is the volume
*enddo
*cfclose

*dim, VOLUME_TABLE, array, 40 ! Create an array to store the volume for each load step, totaling 40 steps

/post1 ! Enter the post-processing module
*do, step, 1, 40, 1 ! Loop through each load step
 SET, step ! Set the current load step
 ALLSEL ! Select all elements and nodes

 ! Select elements with IDs in the range 1 to 15712
 ESEL, S, ELEM, , 1, 15712
 eplo ! Plot elements to visualize the selection

 *get, edtol, elem, 0, count ! Get the total number of selected elements (edtol)
 *get, edmin, elem, 0, num, min ! Get the minimum element ID (edmin)
 ETABLE, ETAB_STRESS, s, eqv ! Define equivalent stress (von Mises stress) as a variable in the stress table

 ! Initialize equivalent stress array
 ESTRESS =
 *dim, ESTRESS,, edtol ! Define the dimensions of the ESTRESS array as the total number of selected elements
 *do, ie, edmin, edtol, 1 ! Loop through all elements (from edmin to edtol)
 *if, ESEL(ie), eq, 1, then ! If the element is selected
 *GET, von, ELEM, ie, ETAB, ETAB_STRESS ! Get the equivalent stress of the current element
 *if, von, gt, 4.0, then ! If the equivalent stress is greater than 4.0
 *if, von, lt, 4.1, then ! And less than 4.1
 ESTRESS(ie) = 1 ! Set the corresponding position in the ESTRESS array to 1 (meets condition)
 *endif
 *endif
 *endif
 *enddo

 ESEL, S, ELEM, , 1, 15712 ! Ensure only elements with IDs in the range 1 to 15712 are selected
 eplo ! Plot elements to visualize the selection
 ESEL, NONE ! Clear all selections
 *do, ie, edmin, edtol, 1 ! Loop through all elements again
 *if, ESTRESS(ie), eq, 1, then ! If the value in the ESTRESS array is 1 (element meets condition)
 ESEL, A,,, ie, ! Add the element to the selection
 *endif
 *enddo
 cm, ee3, elem ! Create a component named "ee3" to store the selected elements

 ! Calculate the total volume
 cmsel, s, ee3, elem ! Select the component "ee3" with the selected elements
 etable, evol, volu ! Define the volume variable
 ssum ! Sum the selected elements to calculate the total volume
 *get, VOLUME_TABLE(step), ssum,, item, evol ! Store the total volume in the VOLUME_TABLE array at the current step position
*enddo

! Output the volume table
*cfopen, volume_output41, txt ! Open a file to output the results
*do, step, 1, 40, 1
 *vwrite, step, VOLUME_TABLE(step)
 (F8.0, ', ', E15.6) ! Output format: first number is the step number, second number is the volume
*enddo
*cfclose

*dim, VOLUME_TABLE, array, 40 ! Create an array to store the volume for each load step, totaling 40 steps

/post1 ! Enter the post-processing module
*do, step, 1, 40, 1 ! Loop through each load step
 SET, step ! Set the current load step
 ALLSEL ! Select all elements and nodes

 ! Select elements with IDs in the range 1 to 15712
 ESEL, S, ELEM, , 1, 15712
 eplo ! Plot elements to visualize the selection

 *get, edtol, elem, 0, count ! Get the total number of selected elements (edtol)
 *get, edmin, elem, 0, num, min ! Get the minimum element ID (edmin)
 ETABLE, ETAB_STRESS, s, eqv ! Define equivalent stress (von Mises stress) as a variable in the stress table

 ! Initialize equivalent stress array
 ESTRESS =
 *dim, ESTRESS,, edtol ! Define the dimensions of the ESTRESS array as the total number of selected elements
 *do, ie, edmin, edtol, 1 ! Loop through all elements (from edmin to edtol)
 *if, ESEL(ie), eq, 1, then ! If the element is selected
 *GET, von, ELEM, ie, ETAB, ETAB_STRESS ! Get the equivalent stress of the current element
 *if, von, gt, 4.1, then ! If the equivalent stress is greater than 4.1
 *if, von, lt, 4.2, then ! And less than 4.2
 ESTRESS(ie) = 1 ! Set the corresponding position in the ESTRESS array to 1 (meets condition)
 *endif
 *endif
 *endif
 *enddo

 ESEL, S, ELEM, , 1, 15712 ! Ensure only elements with IDs in the range 1 to 15712 are selected
 eplo ! Plot elements to visualize the selection
 ESEL, NONE ! Clear all selections
 *do, ie, edmin, edtol, 1 ! Loop through all elements again
 *if, ESTRESS(ie), eq, 1, then ! If the value in the ESTRESS array is 1 (element meets condition)
 ESEL, A,,, ie, ! Add the element to the selection
 *endif
 *enddo
 cm, ee3, elem ! Create a component named "ee3" to store the selected elements

 ! Calculate the total volume
 cmsel, s, ee3, elem ! Select the component "ee3" with the selected elements
 etable, evol, volu ! Define the volume variable
 ssum ! Sum the selected elements to calculate the total volume
 *get, VOLUME_TABLE(step), ssum,, item, evol ! Store the total volume in the VOLUME_TABLE array at the current step position
*enddo

! Output the volume table
*cfopen, volume_output42, txt ! Open a file to output the results
*do, step, 1, 40, 1
 *vwrite, step, VOLUME_TABLE(step)
 (F8.0, ', ', E15.6) ! Output format: first number is the step number, second number is the volume
*enddo
*cfclose

*dim, VOLUME_TABLE, array, 40 ! Create an array to store the volume for each load step, totaling 40 steps

/post1 ! Enter the post-processing module
*do, step, 1, 40, 1 ! Loop through each load step
 SET, step ! Set the current load step
 ALLSEL ! Select all elements and nodes

 ! Select elements with IDs in the range 1 to 15712
 ESEL, S, ELEM, , 1, 15712
 eplo ! Plot elements to visualize the selection

 *get, edtol, elem, 0, count ! Get the total number of selected elements (edtol)
 *get, edmin, elem, 0, num, min ! Get the minimum element ID (edmin)
 ETABLE, ETAB_STRESS, s, eqv ! Define equivalent stress (von Mises stress) as a variable in the stress table

 ! Initialize equivalent stress array
 ESTRESS =
 *dim, ESTRESS,, edtol ! Define the dimensions of the ESTRESS array as the total number of selected elements
 *do, ie, edmin, edtol, 1 ! Loop through all elements (from edmin to edtol)
 *if, ESEL(ie), eq, 1, then ! If the element is selected
 *GET, von, ELEM, ie, ETAB, ETAB_STRESS ! Get the equivalent stress of the current element
 *if, von, gt, 4.2, then ! If the equivalent stress is greater than 4.2
 *if, von, lt, 4.3, then ! And less than 4.3
 ESTRESS(ie) = 1 ! Set the corresponding position in the ESTRESS array to 1 (meets condition)
 *endif
 *endif
 *endif
 *enddo

 ESEL, S, ELEM, , 1, 15712 ! Ensure only elements with IDs in the range 1 to 15712 are selected
 eplo ! Plot elements to visualize the selection
 ESEL, NONE ! Clear all selections
 *do, ie, edmin, edtol, 1 ! Loop through all elements again
 *if, ESTRESS(ie), eq, 1, then ! If the value in the ESTRESS array is 1 (element meets condition)
 ESEL, A,,, ie, ! Add the element to the selection
 *endif
 *enddo
 cm, ee3, elem ! Create a component named "ee3" to store the selected elements

 ! Calculate the total volume
 cmsel, s, ee3, elem ! Select the component "ee3" with the selected elements
 etable, evol, volu ! Define the volume variable
 ssum ! Sum the selected elements to calculate the total volume
 *get, VOLUME_TABLE(step), ssum,, item, evol ! Store the total volume in the VOLUME_TABLE array at the current step position
*enddo

! Output the volume table
*cfopen, volume_output43, txt ! Open a file to output the results
*do, step, 1, 40, 1
 *vwrite, step, VOLUME_TABLE(step)
 (F8.0, ', ', E15.6) ! Output format: first number is the step number, second number is the volume
*enddo
*cfclose

*dim, VOLUME_TABLE, array, 40 ! Create an array to store the volume for each load step, totaling 40 steps

/post1 ! Enter the post-processing module
*do, step, 1, 40, 1 ! Loop through each load step
 SET, step ! Set the current load step
 ALLSEL ! Select all elements and nodes

 ! Select elements with IDs in the range 1 to 15712
 ESEL, S, ELEM, , 1, 15712
 eplo ! Plot elements to visualize the selection

 *get, edtol, elem, 0, count ! Get the total number of selected elements (edtol)
 *get, edmin, elem, 0, num, min ! Get the minimum element ID (edmin)
 ETABLE, ETAB_STRESS, s, eqv ! Define equivalent stress (von Mises stress) as a variable in the stress table

 ! Initialize equivalent stress array
 ESTRESS =
 *dim, ESTRESS,, edtol ! Define the dimensions of the ESTRESS array as the total number of selected elements
 *do, ie, edmin, edtol, 1 ! Loop through all elements (from edmin to edtol)
 *if, ESEL(ie), eq, 1, then ! If the element is selected
 *GET, von, ELEM, ie, ETAB, ETAB_STRESS ! Get the equivalent stress of the current element
 *if, von, gt, 4.3, then ! If the equivalent stress is greater than 4.3
 *if, von, lt, 4.4, then ! And less than 4.4
 ESTRESS(ie) = 1 ! Set the corresponding position in the ESTRESS array to 1 (meets condition)
 *endif
 *endif
 *endif
 *enddo

 ESEL, S, ELEM, , 1, 15712 ! Ensure only elements with IDs in the range 1 to 15712 are selected
 eplo ! Plot elements to visualize the selection
 ESEL, NONE ! Clear all selections
 *do, ie, edmin, edtol, 1 ! Loop through all elements again
 *if, ESTRESS(ie), eq, 1, then ! If the value in the ESTRESS array is 1 (element meets condition)
 ESEL, A,,, ie, ! Add the element to the selection
 *endif
 *enddo
 cm, ee3, elem ! Create a component named "ee3" to store the selected elements

 ! Calculate the total volume
 cmsel, s, ee3, elem ! Select the component "ee3" with the selected elements
 etable, evol, volu ! Define the volume variable
 ssum ! Sum the selected elements to calculate the total volume
 *get, VOLUME_TABLE(step), ssum,, item, evol ! Store the total volume in the VOLUME_TABLE array at the current step position
*enddo

! Output the volume table
*cfopen, volume_output44, txt ! Open a file to output the results
*do, step, 1, 40, 1
 *vwrite, step, VOLUME_TABLE(step)
 (F8.0, ', ', E15.6) ! Output format: first number is the step number, second number is the volume
*enddo
*cfclose

*dim, VOLUME_TABLE, array, 40 ! Create an array to store the volume for each load step, totaling 40 steps

/post1 ! Enter the post-processing module
*do, step, 1, 40, 1 ! Loop through each load step
 SET, step ! Set the current load step
 ALLSEL ! Select all elements and nodes

 ! Select elements with IDs in the range 1 to 15712
 ESEL, S, ELEM, , 1, 15712
 eplo ! Plot elements to visualize the selection

 *get, edtol, elem, 0, count ! Get the total number of selected elements (edtol)
 *get, edmin, elem, 0, num, min ! Get the minimum element ID (edmin)
 ETABLE, ETAB_STRESS, s, eqv ! Define equivalent stress (von Mises stress) as a variable in the stress table

 ! Initialize equivalent stress array
 ESTRESS =
 *dim, ESTRESS,, edtol ! Define the dimensions of the ESTRESS array as the total number of selected elements
 *do, ie, edmin, edtol, 1 ! Loop through all elements (from edmin to edtol)
 *if, ESEL(ie), eq, 1, then ! If the element is selected
 *GET, von, ELEM, ie, ETAB, ETAB_STRESS ! Get the equivalent stress of the current element
 *if, von, gt, 4.4, then ! If the equivalent stress is greater than 4.4
 *if, von, lt, 4.5, then ! And less than 4.5
 ESTRESS(ie) = 1 ! Set the corresponding position in the ESTRESS array to 1 (meets condition)
 *endif
 *endif
 *endif
 *enddo

 ESEL, S, ELEM, , 1, 15712 ! Ensure only elements with IDs in the range 1 to 15712 are selected
 eplo ! Plot elements to visualize the selection
 ESEL, NONE ! Clear all selections
 *do, ie, edmin, edtol, 1 ! Loop through all elements again
 *if, ESTRESS(ie), eq, 1, then ! If the value in the ESTRESS array is 1 (element meets condition)
 ESEL, A,,, ie, ! Add the element to the selection
 *endif
 *enddo
 cm, ee3, elem ! Create a component named "ee3" to store the selected elements

 ! Calculate the total volume
 cmsel, s, ee3, elem ! Select the component "ee3" with the selected elements
 etable, evol, volu ! Define the volume variable
 ssum ! Sum the selected elements to calculate the total volume
 *get, VOLUME_TABLE(step), ssum,, item, evol ! Store the total volume in the VOLUME_TABLE array at the current step position
*enddo

! Output the volume table
*cfopen, volume_output45, txt ! Open a file to output the results
*do, step, 1, 40, 1
 *vwrite, step, VOLUME_TABLE(step)
 (F8.0, ', ', E15.6) ! Output format: first number is the step number, second number is the volume
*enddo
*cfclose

*dim, VOLUME_TABLE, array, 40 ! Create an array to store the volume for each load step, totaling 40 steps

/post1 ! Enter the post-processing module
*do, step, 1, 40, 1 ! Loop through each load step
 SET, step ! Set the current load step
 ALLSEL ! Select all elements and nodes

 ! Select elements with IDs in the range 1 to 15712
 ESEL, S, ELEM, , 1, 15712
 eplo ! Plot elements to visualize the selection

 *get, edtol, elem, 0, count ! Get the total number of selected elements (edtol)
 *get, edmin, elem, 0, num, min ! Get the minimum element ID (edmin)
 ETABLE, ETAB_STRESS, s, eqv ! Define equivalent stress (von Mises stress) as a variable in the stress table

 ! Initialize equivalent stress array
 ESTRESS =
 *dim, ESTRESS,, edtol ! Define the dimensions of the ESTRESS array as the total number of selected elements
 *do, ie, edmin, edtol, 1 ! Loop through all elements (from edmin to edtol)
 *if, ESEL(ie), eq, 1, then ! If the element is selected
 *GET, von, ELEM, ie, ETAB, ETAB_STRESS ! Get the equivalent stress of the current element
 *if, von, gt, 4.5, then ! If the equivalent stress is greater than 4.5
 *if, von, lt, 4.6, then ! And less than 4.6
 ESTRESS(ie) = 1 ! Set the corresponding position in the ESTRESS array to 1 (meets condition)
 *endif
 *endif
 *endif
 *enddo

 ESEL, S, ELEM, , 1, 15712 ! Ensure only elements with IDs in the range 1 to 15712 are selected
 eplo ! Plot elements to visualize the selection
 ESEL, NONE ! Clear all selections
 *do, ie, edmin, edtol, 1 ! Loop through all elements again
 *if, ESTRESS(ie), eq, 1, then ! If the value in the ESTRESS array is 1 (element meets condition)
 ESEL, A,,, ie, ! Add the element to the selection
 *endif
 *enddo
 cm, ee3, elem ! Create a component named "ee3" to store the selected elements

 ! Calculate the total volume
 cmsel, s, ee3, elem ! Select the component "ee3" with the selected elements
 etable, evol, volu ! Define the volume variable
 ssum ! Sum the selected elements to calculate the total volume
 *get, VOLUME_TABLE(step), ssum,, item, evol ! Store the total volume in the VOLUME_TABLE array at the current step position
*enddo

! Output the volume table
*cfopen, volume_output46, txt ! Open a file to output the results
*do, step, 1, 40, 1
 *vwrite, step, VOLUME_TABLE(step)
 (F8.0, ', ', E15.6) ! Output format: first number is the step number, second number is the volume
*enddo
*cfclose

*dim, VOLUME_TABLE, array, 40 ! Create an array to store the volume for each load step, totaling 40 steps

/post1 ! Enter the post-processing module
*do, step, 1, 40, 1 ! Loop through each load step
 SET, step ! Set the current load step
 ALLSEL ! Select all elements and nodes

 ! Select elements with IDs in the range 1 to 15712
 ESEL, S, ELEM, , 1, 15712
 eplo ! Plot elements to visualize the selection

 *get, edtol, elem, 0, count ! Get the total number of selected elements (edtol)
 *get, edmin, elem, 0, num, min ! Get the minimum element ID (edmin)
 ETABLE, ETAB_STRESS, s, eqv ! Define equivalent stress (von Mises stress) as a variable in the stress table

 ! Initialize equivalent stress array
 ESTRESS =
 *dim, ESTRESS,, edtol ! Define the dimensions of the ESTRESS array as the total number of selected elements
 *do, ie, edmin, edtol, 1 ! Loop through all elements (from edmin to edtol)
 *if, ESEL(ie), eq, 1, then ! If the element is selected
 *GET, von, ELEM, ie, ETAB, ETAB_STRESS ! Get the equivalent stress of the current element
 *if, von, gt, 4.6, then ! If the equivalent stress is greater than 4.6
 *if, von, lt, 4.7, then ! And less than 4.7
 ESTRESS(ie) = 1 ! Set the corresponding position in the ESTRESS array to 1 (meets condition)
 *endif
 *endif
 *endif
 *enddo

 ESEL, S, ELEM, , 1, 15712 ! Ensure only elements with IDs in the range 1 to 15712 are selected
 eplo ! Plot elements to visualize the selection
 ESEL, NONE ! Clear all selections
 *do, ie, edmin, edtol, 1 ! Loop through all elements again
 *if, ESTRESS(ie), eq, 1, then ! If the value in the ESTRESS array is 1 (element meets condition)
 ESEL, A,,, ie, ! Add the element to the selection
 *endif
 *enddo
 cm, ee3, elem ! Create a component named "ee3" to store the selected elements

 ! Calculate the total volume
 cmsel, s, ee3, elem ! Select the component "ee3" with the selected elements
 etable, evol, volu ! Define the volume variable
 ssum ! Sum the selected elements to calculate the total volume
 *get, VOLUME_TABLE(step), ssum,, item, evol ! Store the total volume in the VOLUME_TABLE array at the current step position
*enddo

! Output the volume table
*cfopen, volume_output47, txt ! Open a file to output the results
*do, step, 1, 40, 1
 *vwrite, step, VOLUME_TABLE(step)
 (F8.0, ', ', E15.6) ! Output format: first number is the step number, second number is the volume
*enddo
*cfclose

*dim, VOLUME_TABLE, array, 40 ! Create an array to store the volume for each load step, totaling 40 steps

/post1 ! Enter the post-processing module
*do, step, 1, 40, 1 ! Loop through each load step
 SET, step ! Set the current load step
 ALLSEL ! Select all elements and nodes

 ! Select elements with IDs in the range 1 to 15712
 ESEL, S, ELEM, , 1, 15712
 eplo ! Plot elements to visualize the selection

 *get, edtol, elem, 0, count ! Get the total number of selected elements (edtol)
 *get, edmin, elem, 0, num, min ! Get the minimum element ID (edmin)
 ETABLE, ETAB_STRESS, s, eqv ! Define equivalent stress (von Mises stress) as a variable in the stress table

 ! Initialize equivalent stress array
 ESTRESS =
 *dim, ESTRESS,, edtol ! Define the dimensions of the ESTRESS array as the total number of selected elements
 *do, ie, edmin, edtol, 1 ! Loop through all elements (from edmin to edtol)
 *if, ESEL(ie), eq, 1, then ! If the element is selected
 *GET, von, ELEM, ie, ETAB, ETAB_STRESS ! Get the equivalent stress of the current element
 *if, von, gt, 4.7, then ! If the equivalent stress is greater than 4.7
 *if, von, lt, 4.8, then ! And less than 4.8
 ESTRESS(ie) = 1 ! Set the corresponding position in the ESTRESS array to 1 (meets condition)
 *endif
 *endif
 *endif
 *enddo

 ESEL, S, ELEM, , 1, 15712 ! Ensure only elements with IDs in the range 1 to 15712 are selected
 eplo ! Plot elements to visualize the selection
 ESEL, NONE ! Clear all selections
 *do, ie, edmin, edtol, 1 ! Loop through all elements again
 *if, ESTRESS(ie), eq, 1, then ! If the value in the ESTRESS array is 1 (element meets condition)
 ESEL, A,,, ie, ! Add the element to the selection
 *endif
 *enddo
 cm, ee3, elem ! Create a component named "ee3" to store the selected elements

 ! Calculate the total volume
 cmsel, s, ee3, elem ! Select the component "ee3" with the selected elements
 etable, evol, volu ! Define the volume variable
 ssum ! Sum the selected elements to calculate the total volume
 *get, VOLUME_TABLE(step), ssum,, item, evol ! Store the total volume in the VOLUME_TABLE array at the current step position
*enddo

! Output the volume table
*cfopen, volume_output48, txt ! Open a file to output the results
*do, step, 1, 40, 1
 *vwrite, step, VOLUME_TABLE(step)
 (F8.0, ', ', E15.6) ! Output format: first number is the step number, second number is the volume
*enddo
*cfclose

*dim, VOLUME_TABLE, array, 40 ! Create an array to store the volume for each load step, totaling 40 steps

/post1 ! Enter the post-processing module
*do, step, 1, 40, 1 ! Loop through each load step
 SET, step ! Set the current load step
 ALLSEL ! Select all elements and nodes

 ! Select elements with IDs in the range 1 to 15712
 ESEL, S, ELEM, , 1, 15712
 eplo ! Plot elements to visualize the selection

 *get, edtol, elem, 0, count ! Get the total number of selected elements (edtol)
 *get, edmin, elem, 0, num, min ! Get the minimum element ID (edmin)
 ETABLE, ETAB_STRESS, s, eqv ! Define equivalent stress (von Mises stress) as a variable in the stress table

 ! Initialize equivalent stress array
 ESTRESS =
 *dim, ESTRESS,, edtol ! Define the dimensions of the ESTRESS array as the total number of selected elements
 *do, ie, edmin, edtol, 1 ! Loop through all elements (from edmin to edtol)
 *if, ESEL(ie), eq, 1, then ! If the element is selected
 *GET, von, ELEM, ie, ETAB, ETAB_STRESS ! Get the equivalent stress of the current element
 *if, von, gt, 4.8, then ! If the equivalent stress is greater than 4.8
 *if, von, lt, 4.9, then ! And less than 4.9
 ESTRESS(ie) = 1 ! Set the corresponding position in the ESTRESS array to 1 (meets condition)
 *endif
 *endif
 *endif
 *enddo

 ESEL, S, ELEM, , 1, 15712 ! Ensure only elements with IDs in the range 1 to 15712 are selected
 eplo ! Plot elements to visualize the selection
 ESEL, NONE ! Clear all selections
 *do, ie, edmin, edtol, 1 ! Loop through all elements again
 *if, ESTRESS(ie), eq, 1, then ! If the value in the ESTRESS array is 1 (element meets condition)
 ESEL, A,,, ie, ! Add the element to the selection
 *endif
 *enddo
 cm, ee3, elem ! Create a component named "ee3" to store the selected elements

 ! Calculate the total volume
 cmsel, s, ee3, elem ! Select the component "ee3" with the selected elements
 etable, evol, volu ! Define the volume variable
 ssum ! Sum the selected elements to calculate the total volume
 *get, VOLUME_TABLE(step), ssum,, item, evol ! Store the total volume in the VOLUME_TABLE array at the current step position
*enddo

! Output the volume table
*cfopen, volume_output49, txt ! Open a file to output the results
*do, step, 1, 40, 1
 *vwrite, step, VOLUME_TABLE(step)
 (F8.0, ', ', E15.6) ! Output format: first number is the step number, second number is the volume
*enddo
*cfclose

*dim, VOLUME_TABLE, array, 40 ! Create an array to store the volume for each load step, totaling 40 steps

/post1 ! Enter the post-processing module
*do, step, 1, 40, 1 ! Loop through each load step
 SET, step ! Set the current load step
 ALLSEL ! Select all elements and nodes

 ! Select elements with IDs in the range 1 to 15712
 ESEL, S, ELEM, , 1, 15712
 eplo ! Plot elements to visualize the selection

 *get, edtol, elem, 0, count ! Get the total number of selected elements (edtol)
 *get, edmin, elem, 0, num, min ! Get the minimum element ID (edmin)
 ETABLE, ETAB_STRESS, s, eqv ! Define equivalent stress (von Mises stress) as a variable in the stress table

 ! Initialize equivalent stress array
 ESTRESS =
 *dim, ESTRESS,, edtol ! Define the dimensions of the ESTRESS array as the total number of selected elements
 *do, ie, edmin, edtol, 1 ! Loop through all elements (from edmin to edtol)
 *if, ESEL(ie), eq, 1, then ! If the element is selected
 *GET, von, ELEM, ie, ETAB, ETAB_STRESS ! Get the equivalent stress of the current element
 *if, von, gt, 4.9, then ! If the equivalent stress is greater than 4.9
 *if, von, lt, 5.0, then ! And less than 5.0
 ESTRESS(ie) = 1 ! Set the corresponding position in the ESTRESS array to 1 (meets condition)
 *endif
 *endif
 *endif
 *enddo

 ESEL, S, ELEM, , 1, 15712 ! Ensure only elements with IDs in the range 1 to 15712 are selected
 eplo ! Plot elements to visualize the selection
 ESEL, NONE ! Clear all selections
 *do, ie, edmin, edtol, 1 ! Loop through all elements again
 *if, ESTRESS(ie), eq, 1, then ! If the value in the ESTRESS array is 1 (element meets condition)
 ESEL, A,,, ie, ! Add the element to the selection
 *endif
 *enddo
 cm, ee3, elem ! Create a component named "ee3" to store the selected elements

 ! Calculate the total volume
 cmsel, s, ee3, elem ! Select the component "ee3" with the selected elements
 etable, evol, volu ! Define the volume variable
 ssum ! Sum the selected elements to calculate the total volume
 *get, VOLUME_TABLE(step), ssum,, item, evol ! Store the total volume in the VOLUME_TABLE array at the current step position
*enddo

! Output the volume table
*cfopen, volume_output50, txt ! Open a file to output the results
*do, step, 1, 40, 1
 *vwrite, step, VOLUME_TABLE(step)
 (F8.0, ', ', E15.6) ! Output format: first number is the step number, second number is the volume
*enddo
*cfclose

*dim, VOLUME_TABLE, array, 40 ! Create an array to store the volume for each load step, totaling 40 steps

/post1 ! Enter the post-processing module
*do, step, 1, 40, 1 ! Loop through each load step
 SET, step ! Set the current load step
 ALLSEL ! Select all elements and nodes

 ! Select elements with IDs in the range 1 to 15712
 ESEL, S, ELEM, , 1, 15712
 eplo ! Plot elements to visualize the selection

 *get, edtol, elem, 0, count ! Get the total number of selected elements (edtol)
 *get, edmin, elem, 0, num, min ! Get the minimum element ID (edmin)
 ETABLE, ETAB_STRESS, s, eqv ! Define equivalent stress (von Mises stress) as a variable in the stress table

 ! Initialize equivalent stress array
 ESTRESS =
 *dim, ESTRESS,, edtol ! Define the dimensions of the ESTRESS array as the total number of selected elements
 *do, ie, edmin, edtol, 1 ! Loop through all elements (from edmin to edtol)
 *if, ESEL(ie), eq, 1, then ! If the element is selected
 *GET, von, ELEM, ie, ETAB, ETAB_STRESS ! Get the equivalent stress of the current element
 *if, von, gt, 5.0, then ! If the equivalent stress is greater than 5.0
 *if, von, lt, 5.1, then ! And less than 5.1
 ESTRESS(ie) = 1 ! Set the corresponding position in the ESTRESS array to 1 (meets condition)
 *endif
 *endif
 *endif
 *enddo

 ESEL, S, ELEM, , 1, 15712 ! Ensure only elements with IDs in the range 1 to 15712 are selected
 eplo ! Plot elements to visualize the selection
 ESEL, NONE ! Clear all selections
 *do, ie, edmin, edtol, 1 ! Loop through all elements again
 *if, ESTRESS(ie), eq, 1, then ! If the value in the ESTRESS array is 1 (element meets condition)
 ESEL, A,,, ie, ! Add the element to the selection
 *endif
 *enddo
 cm, ee3, elem ! Create a component named "ee3" to store the selected elements

 ! Calculate the total volume
 cmsel, s, ee3, elem ! Select the component "ee3" with the selected elements
 etable, evol, volu ! Define the volume variable
 ssum ! Sum the selected elements to calculate the total volume
 *get, VOLUME_TABLE(step), ssum,, item, evol ! Store the total volume in the VOLUME_TABLE array at the current step position
*enddo

! Output the volume table
*cfopen, volume_output51, txt ! Open a file to output the results
*do, step, 1, 40, 1
 *vwrite, step, VOLUME_TABLE(step)
 (F8.0, ', ', E15.6) ! Output format: first number is the step number, second number is the volume
*enddo
*cfclose

*dim, VOLUME_TABLE, array, 40 ! Create an array to store the volume for each load step, totaling 40 steps

/post1 ! Enter the post-processing module
*do, step, 1, 40, 1 ! Loop through each load step
 SET, step ! Set the current load step
 ALLSEL ! Select all elements and nodes

 ! Select elements with IDs in the range 1 to 15712
 ESEL, S, ELEM, , 1, 15712
 eplo ! Plot elements to visualize the selection

 *get, edtol, elem, 0, count ! Get the total number of selected elements (edtol)
 *get, edmin, elem, 0, num, min ! Get the minimum element ID (edmin)
 ETABLE, ETAB_STRESS, s, eqv ! Define equivalent stress (von Mises stress) as a variable in the stress table

 ! Initialize equivalent stress array
 ESTRESS =
 *dim, ESTRESS,, edtol ! Define the dimensions of the ESTRESS array as the total number of selected elements
 *do, ie, edmin, edtol, 1 ! Loop through all elements (from edmin to edtol)
 *if, ESEL(ie), eq, 1, then ! If the element is selected
 *GET, von, ELEM, ie, ETAB, ETAB_STRESS ! Get the equivalent stress of the current element
 *if, von, gt, 5.1, then ! If the equivalent stress is greater than 5.1
 *if, von, lt, 5.2, then ! And less than 5.2
 ESTRESS(ie) = 1 ! Set the corresponding position in the ESTRESS array to 1 (meets condition)
 *endif
 *endif
 *endif
 *enddo

 ESEL, S, ELEM, , 1, 15712 ! Ensure only elements with IDs in the range 1 to 15712 are selected
 eplo ! Plot elements to visualize the selection
 ESEL, NONE ! Clear all selections
 *do, ie, edmin, edtol, 1 ! Loop through all elements again
 *if, ESTRESS(ie), eq, 1, then ! If the value in the ESTRESS array is 1 (element meets condition)
 ESEL, A,,, ie, ! Add the element to the selection
 *endif
 *enddo
 cm, ee3, elem ! Create a component named "ee3" to store the selected elements

 ! Calculate the total volume
 cmsel, s, ee3, elem ! Select the component "ee3" with the selected elements
 etable, evol, volu ! Define the volume variable
 ssum ! Sum the selected elements to calculate the total volume
 *get, VOLUME_TABLE(step), ssum,, item, evol ! Store the total volume in the VOLUME_TABLE array at the current step position
*enddo

! Output the volume table
*cfopen, volume_output52, txt ! Open a file to output the results
*do, step, 1, 40, 1
 *vwrite, step, VOLUME_TABLE(step)
 (F8.0, ', ', E15.6) ! Output format: first number is the step number, second number is the volume
*enddo
*cfclose

*dim, VOLUME_TABLE, array, 40 ! Create an array to store the volume for each load step, totaling 40 steps

/post1 ! Enter the post-processing module
*do, step, 1, 40, 1 ! Loop through each load step
 SET, step ! Set the current load step
 ALLSEL ! Select all elements and nodes

 ! Select elements with IDs in the range 1 to 15712
 ESEL, S, ELEM, , 1, 15712
 eplo ! Plot elements to visualize the selection

 *get, edtol, elem, 0, count ! Get the total number of selected elements (edtol)
 *get, edmin, elem, 0, num, min ! Get the minimum element ID (edmin)
 ETABLE, ETAB_STRESS, s, eqv ! Define equivalent stress (von Mises stress) as a variable in the stress table

 ! Initialize equivalent stress array
 ESTRESS =
 *dim, ESTRESS,, edtol ! Define the dimensions of the ESTRESS array as the total number of selected elements
 *do, ie, edmin, edtol, 1 ! Loop through all elements (from edmin to edtol)
 *if, ESEL(ie), eq, 1, then ! If the element is selected
 *GET, von, ELEM, ie, ETAB, ETAB_STRESS ! Get the equivalent stress of the current element
 *if, von, gt, 5.2, then ! If the equivalent stress is greater than 5.2
 *if, von, lt, 5.3, then ! And less than 5.3
 ESTRESS(ie) = 1 ! Set the corresponding position in the ESTRESS array to 1 (meets condition)
 *endif
 *endif
 *endif
 *enddo

 ESEL, S, ELEM, , 1, 15712 ! Ensure only elements with IDs in the range 1 to 15712 are selected
 eplo ! Plot elements to visualize the selection
 ESEL, NONE ! Clear all selections
 *do, ie, edmin, edtol, 1 ! Loop through all elements again
 *if, ESTRESS(ie), eq, 1, then ! If the value in the ESTRESS array is 1 (element meets condition)
 ESEL, A,,, ie, ! Add the element to the selection
 *endif
 *enddo
 cm, ee3, elem ! Create a component named "ee3" to store the selected elements

 ! Calculate the total volume
 cmsel, s, ee3, elem ! Select the component "ee3" with the selected elements
 etable, evol, volu ! Define the volume variable
 ssum ! Sum the selected elements to calculate the total volume
 *get, VOLUME_TABLE(step), ssum,, item, evol ! Store the total volume in the VOLUME_TABLE array at the current step position
*enddo

! Output the volume table
*cfopen, volume_output53, txt ! Open a file to output the results
*do, step, 1, 40, 1
 *vwrite, step, VOLUME_TABLE(step)
 (F8.0, ', ', E15.6) ! Output format: first number is the step number, second number is the volume
*enddo
*cfclose

*dim, VOLUME_TABLE, array, 40 ! Create an array to store the volume for each load step, totaling 40 steps

/post1 ! Enter the post-processing module
*do, step, 1, 40, 1 ! Loop through each load step
 SET, step ! Set the current load step
 ALLSEL ! Select all elements and nodes

 ! Select elements with IDs in the range 1 to 15712
 ESEL, S, ELEM, , 1, 15712
 eplo ! Plot elements to visualize the selection

 *get, edtol, elem, 0, count ! Get the total number of selected elements (edtol)
 *get, edmin, elem, 0, num, min ! Get the minimum element ID (edmin)
 ETABLE, ETAB_STRESS, s, eqv ! Define equivalent stress (von Mises stress) as a variable in the stress table

 ! Initialize equivalent stress array
 ESTRESS =
 *dim, ESTRESS,, edtol ! Define the dimensions of the ESTRESS array as the total number of selected elements
 *do, ie, edmin, edtol, 1 ! Loop through all elements (from edmin to edtol)
 *if, ESEL(ie), eq, 1, then ! If the element is selected
 *GET, von, ELEM, ie, ETAB, ETAB_STRESS ! Get the equivalent stress of the current element
 *if, von, gt, 5.3, then ! If the equivalent stress is greater than 5.3
 *if, von, lt, 5.4, then ! And less than 5.4
 ESTRESS(ie) = 1 ! Set the corresponding position in the ESTRESS array to 1 (meets condition)
 *endif
 *endif
 *endif
 *enddo

 ESEL, S, ELEM, , 1, 15712 ! Ensure only elements with IDs in the range 1 to 15712 are selected
 eplo ! Plot elements to visualize the selection
 ESEL, NONE ! Clear all selections
 *do, ie, edmin, edtol, 1 ! Loop through all elements again
 *if, ESTRESS(ie), eq, 1, then ! If the value in the ESTRESS array is 1 (element meets condition)
 ESEL, A,,, ie, ! Add the element to the selection
 *endif
 *enddo
 cm, ee3, elem ! Create a component named "ee3" to store the selected elements

 ! Calculate the total volume
 cmsel, s, ee3, elem ! Select the component "ee3" with the selected elements
 etable, evol, volu ! Define the volume variable
 ssum ! Sum the selected elements to calculate the total volume
 *get, VOLUME_TABLE(step), ssum,, item, evol ! Store the total volume in the VOLUME_TABLE array at the current step position
*enddo

! Output the volume table
*cfopen, volume_output54, txt ! Open a file to output the results
*do, step, 1, 40, 1
 *vwrite, step, VOLUME_TABLE(step)
 (F8.0, ', ', E15.6) ! Output format: first number is the step number, second number is the volume
*enddo
*cfclose

*dim, VOLUME_TABLE, array, 40 ! Create an array to store the volume for each load step, totaling 40 steps

/post1 ! Enter the post-processing module
*do, step, 1, 40, 1 ! Loop through each load step
 SET, step ! Set the current load step
 ALLSEL ! Select all elements and nodes

 ! Select elements with IDs in the range 1 to 15712
 ESEL, S, ELEM, , 1, 15712
 eplo ! Plot elements to visualize the selection

 *get, edtol, elem, 0, count ! Get the total number of selected elements (edtol)
 *get, edmin, elem, 0, num, min ! Get the minimum element ID (edmin)
 ETABLE, ETAB_STRESS, s, eqv ! Define equivalent stress (von Mises stress) as a variable in the stress table

 ! Initialize equivalent stress array
 ESTRESS =
 *dim, ESTRESS,, edtol ! Define the dimensions of the ESTRESS array as the total number of selected elements
 *do, ie, edmin, edtol, 1 ! Loop through all elements (from edmin to edtol)
 *if, ESEL(ie), eq, 1, then ! If the element is selected
 *GET, von, ELEM, ie, ETAB, ETAB_STRESS ! Get the equivalent stress of the current element
 *if, von, gt, 5.4, then ! If the equivalent stress is greater than 5.4
 *if, von, lt, 5.5, then ! And less than 5.5
 ESTRESS(ie) = 1 ! Set the corresponding position in the ESTRESS array to 1 (meets condition)
 *endif
 *endif
 *endif
 *enddo

 ESEL, S, ELEM, , 1, 15712 ! Ensure only elements with IDs in the range 1 to 15712 are selected
 eplo ! Plot elements to visualize the selection
 ESEL, NONE ! Clear all selections
 *do, ie, edmin, edtol, 1 ! Loop through all elements again
 *if, ESTRESS(ie), eq, 1, then ! If the value in the ESTRESS array is 1 (element meets condition)
 ESEL, A,,, ie, ! Add the element to the selection
 *endif
 *enddo
 cm, ee3, elem ! Create a component named "ee3" to store the selected elements

 ! Calculate the total volume
 cmsel, s, ee3, elem ! Select the component "ee3" with the selected elements
 etable, evol, volu ! Define the volume variable
 ssum ! Sum the selected elements to calculate the total volume
 *get, VOLUME_TABLE(step), ssum,, item, evol ! Store the total volume in the VOLUME_TABLE array at the current step position
*enddo

! Output the volume table
*cfopen, volume_output55, txt ! Open a file to output the results
*do, step, 1, 40, 1
 *vwrite, step, VOLUME_TABLE(step)
 (F8.0, ', ', E15.6) ! Output format: first number is the step number, second number is the volume
*enddo
*cfclose

*dim, VOLUME_TABLE, array, 40 ! Create an array to store the volume for each load step, totaling 40 steps

/post1 ! Enter the post-processing module
*do, step, 1, 40, 1 ! Loop through each load step
 SET, step ! Set the current load step
 ALLSEL ! Select all elements and nodes

 ! Select elements with IDs in the range 1 to 15712
 ESEL, S, ELEM, , 1, 15712
 eplo ! Plot elements to visualize the selection

 *get, edtol, elem, 0, count ! Get the total number of selected elements (edtol)
 *get, edmin, elem, 0, num, min ! Get the minimum element ID (edmin)
 ETABLE, ETAB_STRESS, s, eqv ! Define equivalent stress (von Mises stress) as a variable in the stress table

 ! Initialize equivalent stress array
 ESTRESS =
 *dim, ESTRESS,, edtol ! Define the dimensions of the ESTRESS array as the total number of selected elements
 *do, ie, edmin, edtol, 1 ! Loop through all elements (from edmin to edtol)
 *if, ESEL(ie), eq, 1, then ! If the element is selected
 *GET, von, ELEM, ie, ETAB, ETAB_STRESS ! Get the equivalent stress of the current element
 *if, von, gt, 5.5, then ! If the equivalent stress is greater than 5.5
 *if, von, lt, 5.6, then ! And less than 5.6
 ESTRESS(ie) = 1 ! Set the corresponding position in the ESTRESS array to 1 (meets condition)
 *endif
 *endif
 *endif
 *enddo

 ESEL, S, ELEM, , 1, 15712 ! Ensure only elements with IDs in the range 1 to 15712 are selected
 eplo ! Plot elements to visualize the selection
 ESEL, NONE ! Clear all selections
 *do, ie, edmin, edtol, 1 ! Loop through all elements again
 *if, ESTRESS(ie), eq, 1, then ! If the value in the ESTRESS array is 1 (element meets condition)
 ESEL, A,,, ie, ! Add the element to the selection
 *endif
 *enddo
 cm, ee3, elem ! Create a component named "ee3" to store the selected elements

 ! Calculate the total volume
 cmsel, s, ee3, elem ! Select the component "ee3" with the selected elements
 etable, evol, volu ! Define the volume variable
 ssum ! Sum the selected elements to calculate the total volume
 *get, VOLUME_TABLE(step), ssum,, item, evol ! Store the total volume in the VOLUME_TABLE array at the current step position
*enddo

! Output the volume table
*cfopen, volume_output56, txt ! Open a file to output the results
*do, step, 1, 40, 1
 *vwrite, step, VOLUME_TABLE(step)
 (F8.0, ', ', E15.6) ! Output format: first number is the step number, second number is the volume
*enddo
*cfclose

*dim, VOLUME_TABLE, array, 40 ! Create an array to store the volume for each load step, totaling 40 steps

/post1 ! Enter the post-processing module
*do, step, 1, 40, 1 ! Loop through each load step
 SET, step ! Set the current load step
 ALLSEL ! Select all elements and nodes

 ! Select elements with IDs in the range 1 to 15712
 ESEL, S, ELEM, , 1, 15712
 eplo ! Plot elements to visualize the selection

 *get, edtol, elem, 0, count ! Get the total number of selected elements (edtol)
 *get, edmin, elem, 0, num, min ! Get the minimum element ID (edmin)
 ETABLE, ETAB_STRESS, s, eqv ! Define equivalent stress (von Mises stress) as a variable in the stress table

 ! Initialize equivalent stress array
 ESTRESS =
 *dim, ESTRESS,, edtol ! Define the dimensions of the ESTRESS array as the total number of selected elements
 *do, ie, edmin, edtol, 1 ! Loop through all elements (from edmin to edtol)
 *if, ESEL(ie), eq, 1, then ! If the element is selected
 *GET, von, ELEM, ie, ETAB, ETAB_STRESS ! Get the equivalent stress of the current element
 *if, von, gt, 5.6, then ! If the equivalent stress is greater than 5.6
 *if, von, lt, 5.7, then ! And less than 5.7
 ESTRESS(ie) = 1 ! Set the corresponding position in the ESTRESS array to 1 (meets condition)
 *endif
 *endif
 *endif
 *enddo

 ESEL, S, ELEM, , 1, 15712 ! Ensure only elements with IDs in the range 1 to 15712 are selected
 eplo ! Plot elements to visualize the selection
 ESEL, NONE ! Clear all selections
 *do, ie, edmin, edtol, 1 ! Loop through all elements again
 *if, ESTRESS(ie), eq, 1, then ! If the value in the ESTRESS array is 1 (element meets condition)
 ESEL, A,,, ie, ! Add the element to the selection
 *endif
 *enddo
 cm, ee3, elem ! Create a component named "ee3" to store the selected elements

 ! Calculate the total volume
 cmsel, s, ee3, elem ! Select the component "ee3" with the selected elements
 etable, evol, volu ! Define the volume variable
 ssum ! Sum the selected elements to calculate the total volume
 *get, VOLUME_TABLE(step), ssum,, item, evol ! Store the total volume in the VOLUME_TABLE array at the current step position
*enddo

! Output the volume table
*cfopen, volume_output57, txt ! Open a file to output the results
*do, step, 1, 40, 1
 *vwrite, step, VOLUME_TABLE(step)
 (F8.0, ', ', E15.6) ! Output format: first number is the step number, second number is the volume
*enddo
*cfclose

*dim, VOLUME_TABLE, array, 40 ! Create an array to store the volume for each load step, totaling 40 steps

/post1 ! Enter the post-processing module
*do, step, 1, 40, 1 ! Loop through each load step
 SET, step ! Set the current load step
 ALLSEL ! Select all elements and nodes

 ! Select elements with IDs in the range 1 to 15712
 ESEL, S, ELEM, , 1, 15712
 eplo ! Plot elements to visualize the selection

 *get, edtol, elem, 0, count ! Get the total number of selected elements (edtol)
 *get, edmin, elem, 0, num, min ! Get the minimum element ID (edmin)
 ETABLE, ETAB_STRESS, s, eqv ! Define equivalent stress (von Mises stress) as a variable in the stress table

 ! Initialize equivalent stress array
 ESTRESS =
 *dim, ESTRESS,, edtol ! Define the dimensions of the ESTRESS array as the total number of selected elements
 *do, ie, edmin, edtol, 1 ! Loop through all elements (from edmin to edtol)
 *if, ESEL(ie), eq, 1, then ! If the element is selected
 *GET, von, ELEM, ie, ETAB, ETAB_STRESS ! Get the equivalent stress of the current element
 *if, von, gt, 5.7, then ! If the equivalent stress is greater than 5.7
 *if, von, lt, 5.8, then ! And less than 5.8
 ESTRESS(ie) = 1 ! Set the corresponding position in the ESTRESS array to 1 (meets condition)
 *endif
 *endif
 *endif
 *enddo

 ESEL, S, ELEM, , 1, 15712 ! Ensure only elements with IDs in the range 1 to 15712 are selected
 eplo ! Plot elements to visualize the selection
 ESEL, NONE ! Clear all selections
 *do, ie, edmin, edtol, 1 ! Loop through all elements again
 *if, ESTRESS(ie), eq, 1, then ! If the value in the ESTRESS array is 1 (element meets condition)
 ESEL, A,,, ie, ! Add the element to the selection
 *endif
 *enddo
 cm, ee3, elem ! Create a component named "ee3" to store the selected elements

 ! Calculate the total volume
 cmsel, s, ee3, elem ! Select the component "ee3" with the selected elements
 etable, evol, volu ! Define the volume variable
 ssum ! Sum the selected elements to calculate the total volume
 *get, VOLUME_TABLE(step), ssum,, item, evol ! Store the total volume in the VOLUME_TABLE array at the current step position
*enddo

! Output the volume table
*cfopen, volume_output58, txt ! Open a file to output the results
*do, step, 1, 40, 1
 *vwrite, step, VOLUME_TABLE(step)
 (F8.0, ', ', E15.6) ! Output format: first number is the step number, second number is the volume
*enddo
*cfclose

*dim, VOLUME_TABLE, array, 40 ! Create an array to store the volume for each load step, totaling 40 steps

/post1 ! Enter the post-processing module
*do, step, 1, 40, 1 ! Loop through each load step
 SET, step ! Set the current load step
 ALLSEL ! Select all elements and nodes

 ! Select elements with IDs in the range 1 to 15712
 ESEL, S, ELEM, , 1, 15712
 eplo ! Plot elements to visualize the selection

 *get, edtol, elem, 0, count ! Get the total number of selected elements (edtol)
 *get, edmin, elem, 0, num, min ! Get the minimum element ID (edmin)
 ETABLE, ETAB_STRESS, s, eqv ! Define equivalent stress (von Mises stress) as a variable in the stress table

 ! Initialize equivalent stress array
 ESTRESS =
 *dim, ESTRESS,, edtol ! Define the dimensions of the ESTRESS array as the total number of selected elements
 *do, ie, edmin, edtol, 1 ! Loop through all elements (from edmin to edtol)
 *if, ESEL(ie), eq, 1, then ! If the element is selected
 *GET, von, ELEM, ie, ETAB, ETAB_STRESS ! Get the equivalent stress of the current element
 *if, von, gt, 5.8, then ! If the equivalent stress is greater than 5.8
 *if, von, lt, 5.9, then ! And less than 5.9
 ESTRESS(ie) = 1 ! Set the corresponding position in the ESTRESS array to 1 (meets condition)
 *endif
 *endif
 *endif
 *enddo

 ESEL, S, ELEM, , 1, 15712 ! Ensure only elements with IDs in the range 1 to 15712 are selected
 eplo ! Plot elements to visualize the selection
 ESEL, NONE ! Clear all selections
 *do, ie, edmin, edtol, 1 ! Loop through all elements again
 *if, ESTRESS(ie), eq, 1, then ! If the value in the ESTRESS array is 1 (element meets condition)
 ESEL, A,,, ie, ! Add the element to the selection
 *endif
 *enddo
 cm, ee3, elem ! Create a component named "ee3" to store the selected elements

 ! Calculate the total volume
 cmsel, s, ee3, elem ! Select the component "ee3" with the selected elements
 etable, evol, volu ! Define the volume variable
 ssum ! Sum the selected elements to calculate the total volume
 *get, VOLUME_TABLE(step), ssum,, item, evol ! Store the total volume in the VOLUME_TABLE array at the current step position
*enddo

! Output the volume table
*cfopen, volume_output59, txt ! Open a file to output the results
*do, step, 1, 40, 1
 *vwrite, step, VOLUME_TABLE(step)
 (F8.0, ', ', E15.6) ! Output format: first number is the step number, second number is the volume
*enddo
*cfclose

*dim, VOLUME_TABLE, array, 40 ! Create an array to store the volume for each load step, totaling 40 steps

/post1 ! Enter the post-processing module
*do, step, 1, 40, 1 ! Loop through each load step
 SET, step ! Set the current load step
 ALLSEL ! Select all elements and nodes

 ! Select elements with IDs in the range 1 to 15712
 ESEL, S, ELEM, , 1, 15712
 eplo ! Plot elements to visualize the selection

 *get, edtol, elem, 0, count ! Get the total number of selected elements (edtol)
 *get, edmin, elem, 0, num, min ! Get the minimum element ID (edmin)
 ETABLE, ETAB_STRESS, s, eqv ! Define equivalent stress (von Mises stress) as a variable in the stress table

 ! Initialize equivalent stress array
 ESTRESS =
 *dim, ESTRESS,, edtol ! Define the dimensions of the ESTRESS array as the total number of selected elements
 *do, ie, edmin, edtol, 1 ! Loop through all elements (from edmin to edtol)
 *if, ESEL(ie), eq, 1, then ! If the element is selected
 *GET, von, ELEM, ie, ETAB, ETAB_STRESS ! Get the equivalent stress of the current element
 *if, von, gt, 5.9, then ! If the equivalent stress is greater than 5.9
 *if, von, lt, 6.0, then ! And less than 6.0
 ESTRESS(ie) = 1 ! Set the corresponding position in the ESTRESS array to 1 (meets condition)
 *endif
 *endif
 *endif
 *enddo

 ESEL, S, ELEM, , 1, 15712 ! Ensure only elements with IDs in the range 1 to 15712 are selected
 eplo ! Plot elements to visualize the selection
 ESEL, NONE ! Clear all selections
 *do, ie, edmin, edtol, 1 ! Loop through all elements again
 *if, ESTRESS(ie), eq, 1, then ! If the value in the ESTRESS array is 1 (element meets condition)
 ESEL, A,,, ie, ! Add the element to the selection
 *endif
 *enddo
 cm, ee3, elem ! Create a component named "ee3" to store the selected elements

 ! Calculate the total volume
 cmsel, s, ee3, elem ! Select the component "ee3" with the selected elements
 etable, evol, volu ! Define the volume variable
 ssum ! Sum the selected elements to calculate the total volume
 *get, VOLUME_TABLE(step), ssum,, item, evol ! Store the total volume in the VOLUME_TABLE array at the current step position
*enddo

! Output the volume table
*cfopen, volume_output60, txt ! Open a file to output the results
*do, step, 1, 40, 1
 *vwrite, step, VOLUME_TABLE(step)
 (F8.0, ', ', E15.6) ! Output format: first number is the step number, second number is the volume
*enddo
*cfclose

*dim, VOLUME_TABLE, array, 40 ! Create an array to store the volume for each load step, totaling 40 steps

/post1 ! Enter the post-processing module
*do, step, 1, 40, 1 ! Loop through each load step
 SET, step ! Set the current load step
 ALLSEL ! Select all elements and nodes

 ! Select elements with IDs in the range 1 to 15712
 ESEL, S, ELEM, , 1, 15712
 eplo ! Plot elements to visualize the selection

 *get, edtol, elem, 0, count ! Get the total number of selected elements (edtol)
 *get, edmin, elem, 0, num, min ! Get the minimum element ID (edmin)
 ETABLE, ETAB_STRESS, s, eqv ! Define equivalent stress (von Mises stress) as a variable in the stress table

 ! Initialize equivalent stress array
 ESTRESS =
 *dim, ESTRESS,, edtol ! Define the dimensions of the ESTRESS array as the total number of selected elements
 *do, ie, edmin, edtol, 1 ! Loop through all elements (from edmin to edtol)
 *if, ESEL(ie), eq, 1, then ! If the element is selected
 *GET, von, ELEM, ie, ETAB, ETAB_STRESS ! Get the equivalent stress of the current element
 *if, von, gt, 6.0, then ! If the equivalent stress is greater than 6.0
 *if, von, lt, 6.1, then ! And less than 6.1
 ESTRESS(ie) = 1 ! Set the corresponding position in the ESTRESS array to 1 (meets condition)
 *endif
 *endif
 *endif
 *enddo

 ESEL, S, ELEM, , 1, 15712 ! Ensure only elements with IDs in the range 1 to 15712 are selected
 eplo ! Plot elements to visualize the selection
 ESEL, NONE ! Clear all selections
 *do, ie, edmin, edtol, 1 ! Loop through all elements again
 *if, ESTRESS(ie), eq, 1, then ! If the value in the ESTRESS array is 1 (element meets condition)
 ESEL, A,,, ie, ! Add the element to the selection
 *endif
 *enddo
 cm, ee3, elem ! Create a component named "ee3" to store the selected elements

 ! Calculate the total volume
 cmsel, s, ee3, elem ! Select the component "ee3" with the selected elements
 etable, evol, volu ! Define the volume variable
 ssum ! Sum the selected elements to calculate the total volume
 *get, VOLUME_TABLE(step), ssum,, item, evol ! Store the total volume in the VOLUME_TABLE array at the current step position
*enddo

! Output the volume table
*cfopen, volume_output61, txt ! Open a file to output the results
*do, step, 1, 40, 1
 *vwrite, step, VOLUME_TABLE(step)
 (F8.0, ', ', E15.6) ! Output format: first number is the step number, second number is the volume
*enddo
*cfclose

*dim, VOLUME_TABLE, array, 40 ! Create an array to store the volume for each load step, totaling 40 steps

/post1 ! Enter the post-processing module
*do, step, 1, 40, 1 ! Loop through each load step
 SET, step ! Set the current load step
 ALLSEL ! Select all elements and nodes

 ! Select elements with IDs in the range 1 to 15712
 ESEL, S, ELEM, , 1, 15712
 eplo ! Plot elements to visualize the selection

 *get, edtol, elem, 0, count ! Get the total number of selected elements (edtol)
 *get, edmin, elem, 0, num, min ! Get the minimum element ID (edmin)
 ETABLE, ETAB_STRESS, s, eqv ! Define equivalent stress (von Mises stress) as a variable in the stress table

 ! Initialize equivalent stress array
 ESTRESS =
 *dim, ESTRESS,, edtol ! Define the dimensions of the ESTRESS array as the total number of selected elements
 *do, ie, edmin, edtol, 1 ! Loop through all elements (from edmin to edtol)
 *if, ESEL(ie), eq, 1, then ! If the element is selected
 *GET, von, ELEM, ie, ETAB, ETAB_STRESS ! Get the equivalent stress of the current element
 *if, von, gt, 6.1, then ! If the equivalent stress is greater than 6.1
 *if, von, lt, 6.2, then ! And less than 6.2
 ESTRESS(ie) = 1 ! Set the corresponding position in the ESTRESS array to 1 (meets condition)
 *endif
 *endif
 *endif
 *enddo

 ESEL, S, ELEM, , 1, 15712 ! Ensure only elements with IDs in the range 1 to 15712 are selected
 eplo ! Plot elements to visualize the selection
 ESEL, NONE ! Clear all selections
 *do, ie, edmin, edtol, 1 ! Loop through all elements again
 *if, ESTRESS(ie), eq, 1, then ! If the value in the ESTRESS array is 1 (element meets condition)
 ESEL, A,,, ie, ! Add the element to the selection
 *endif
 *enddo
 cm, ee3, elem ! Create a component named "ee3" to store the selected elements

 ! Calculate the total volume
 cmsel, s, ee3, elem ! Select the component "ee3" with the selected elements
 etable, evol, volu ! Define the volume variable
 ssum ! Sum the selected elements to calculate the total volume
 *get, VOLUME_TABLE(step), ssum,, item, evol ! Store the total volume in the VOLUME_TABLE array at the current step position
*enddo

! Output the volume table
*cfopen, volume_output62, txt ! Open a file to output the results
*do, step, 1, 40, 1
 *vwrite, step, VOLUME_TABLE(step)
 (F8.0, ', ', E15.6) ! Output format: first number is the step number, second number is the volume
*enddo
*cfclose

*dim, VOLUME_TABLE, array, 40 ! Create an array to store the volume for each load step, totaling 40 steps

/post1 ! Enter the post-processing module
*do, step, 1, 40, 1 ! Loop through each load step
 SET, step ! Set the current load step
 ALLSEL ! Select all elements and nodes

 ! Select elements with IDs in the range 1 to 15712
 ESEL, S, ELEM, , 1, 15712
 eplo ! Plot elements to visualize the selection

 *get, edtol, elem, 0, count ! Get the total number of selected elements (edtol)
 *get, edmin, elem, 0, num, min ! Get the minimum element ID (edmin)
 ETABLE, ETAB_STRESS, s, eqv ! Define equivalent stress (von Mises stress) as a variable in the stress table

 ! Initialize equivalent stress array
 ESTRESS =
 *dim, ESTRESS,, edtol ! Define the dimensions of the ESTRESS array as the total number of selected elements
 *do, ie, edmin, edtol, 1 ! Loop through all elements (from edmin to edtol)
 *if, ESEL(ie), eq, 1, then ! If the element is selected
 *GET, von, ELEM, ie, ETAB, ETAB_STRESS ! Get the equivalent stress of the current element
 *if, von, gt, 6.2, then ! If the equivalent stress is greater than 6.2
 *if, von, lt, 6.3, then ! And less than 6.3
 ESTRESS(ie) = 1 ! Set the corresponding position in the ESTRESS array to 1 (meets condition)
 *endif
 *endif
 *endif
 *enddo

 ESEL, S, ELEM, , 1, 15712 ! Ensure only elements with IDs in the range 1 to 15712 are selected
 eplo ! Plot elements to visualize the selection
 ESEL, NONE ! Clear all selections
 *do, ie, edmin, edtol, 1 ! Loop through all elements again
 *if, ESTRESS(ie), eq, 1, then ! If the value in the ESTRESS array is 1 (element meets condition)
 ESEL, A,,, ie, ! Add the element to the selection
 *endif
 *enddo
 cm, ee3, elem ! Create a component named "ee3" to store the selected elements

 ! Calculate the total volume
 cmsel, s, ee3, elem ! Select the component "ee3" with the selected elements
 etable, evol, volu ! Define the volume variable
 ssum ! Sum the selected elements to calculate the total volume
 *get, VOLUME_TABLE(step), ssum,, item, evol ! Store the total volume in the VOLUME_TABLE array at the current step position
*enddo

! Output the volume table
*cfopen, volume_output63, txt ! Open a file to output the results
*do, step, 1, 40, 1
 *vwrite, step, VOLUME_TABLE(step)
 (F8.0, ', ', E15.6) ! Output format: first number is the step number, second number is the volume
*enddo
*cfclose

*dim, VOLUME_TABLE, array, 40 ! Create an array to store the volume for each load step, totaling 40 steps

/post1 ! Enter the post-processing module
*do, step, 1, 40, 1 ! Loop through each load step
 SET, step ! Set the current load step
 ALLSEL ! Select all elements and nodes

 ! Select elements with IDs in the range 1 to 15712
 ESEL, S, ELEM, , 1, 15712
 eplo ! Plot elements to visualize the selection

 *get, edtol, elem, 0, count ! Get the total number of selected elements (edtol)
 *get, edmin, elem, 0, num, min ! Get the minimum element ID (edmin)
 ETABLE, ETAB_STRESS, s, eqv ! Define equivalent stress (von Mises stress) as a variable in the stress table

 ! Initialize equivalent stress array
 ESTRESS =
 *dim, ESTRESS,, edtol ! Define the dimensions of the ESTRESS array as the total number of selected elements
 *do, ie, edmin, edtol, 1 ! Loop through all elements (from edmin to edtol)
 *if, ESEL(ie), eq, 1, then ! If the element is selected
 *GET, von, ELEM, ie, ETAB, ETAB_STRESS ! Get the equivalent stress of the current element
 *if, von, gt, 6.3, then ! If the equivalent stress is greater than 6.3
 *if, von, lt, 6.4, then ! And less than 6.4
 ESTRESS(ie) = 1 ! Set the corresponding position in the ESTRESS array to 1 (meets condition)
 *endif
 *endif
 *endif
 *enddo

 ESEL, S, ELEM, , 1, 15712 ! Ensure only elements with IDs in the range 1 to 15712 are selected
 eplo ! Plot elements to visualize the selection
 ESEL, NONE ! Clear all selections
 *do, ie, edmin, edtol, 1 ! Loop through all elements again
 *if, ESTRESS(ie), eq, 1, then ! If the value in the ESTRESS array is 1 (element meets condition)
 ESEL, A,,, ie, ! Add the element to the selection
 *endif
 *enddo
 cm, ee3, elem ! Create a component named "ee3" to store the selected elements

 ! Calculate the total volume
 cmsel, s, ee3, elem ! Select the component "ee3" with the selected elements
 etable, evol, volu ! Define the volume variable
 ssum ! Sum the selected elements to calculate the total volume
 *get, VOLUME_TABLE(step), ssum,, item, evol ! Store the total volume in the VOLUME_TABLE array at the current step position
*enddo

! Output the volume table
*cfopen, volume_output64, txt ! Open a file to output the results
*do, step, 1, 40, 1
 *vwrite, step, VOLUME_TABLE(step)
 (F8.0, ', ', E15.6) ! Output format: first number is the step number, second number is the volume
*enddo
*cfclose

*dim, VOLUME_TABLE, array, 40 ! Create an array to store the volume for each load step, totaling 40 steps

/post1 ! Enter the post-processing module
*do, step, 1, 40, 1 ! Loop through each load step
 SET, step ! Set the current load step
 ALLSEL ! Select all elements and nodes

 ! Select elements with IDs in the range 1 to 15712
 ESEL, S, ELEM, , 1, 15712
 eplo ! Plot elements to visualize the selection

 *get, edtol, elem, 0, count ! Get the total number of selected elements (edtol)
 *get, edmin, elem, 0, num, min ! Get the minimum element ID (edmin)
 ETABLE, ETAB_STRESS, s, eqv ! Define equivalent stress (von Mises stress) as a variable in the stress table

 ! Initialize equivalent stress array
 ESTRESS =
 *dim, ESTRESS,, edtol ! Define the dimensions of the ESTRESS array as the total number of selected elements
 *do, ie, edmin, edtol, 1 ! Loop through all elements (from edmin to edtol)
 *if, ESEL(ie), eq, 1, then ! If the element is selected
 *GET, von, ELEM, ie, ETAB, ETAB_STRESS ! Get the equivalent stress of the current element
 *if, von, gt, 6.4, then ! If the equivalent stress is greater than 6.4
 *if, von, lt, 6.5, then ! And less than 6.5
 ESTRESS(ie) = 1 ! Set the corresponding position in the ESTRESS array to 1 (meets condition)
 *endif
 *endif
 *endif
 *enddo

 ESEL, S, ELEM, , 1, 15712 ! Ensure only elements with IDs in the range 1 to 15712 are selected
 eplo ! Plot elements to visualize the selection
 ESEL, NONE ! Clear all selections
 *do, ie, edmin, edtol, 1 ! Loop through all elements again
 *if, ESTRESS(ie), eq, 1, then ! If the value in the ESTRESS array is 1 (element meets condition)
 ESEL, A,,, ie, ! Add the element to the selection
 *endif
 *enddo
 cm, ee3, elem ! Create a component named "ee3" to store the selected elements

 ! Calculate the total volume
 cmsel, s, ee3, elem ! Select the component "ee3" with the selected elements
 etable, evol, volu ! Define the volume variable
 ssum ! Sum the selected elements to calculate the total volume
 *get, VOLUME_TABLE(step), ssum,, item, evol ! Store the total volume in the VOLUME_TABLE array at the current step position
*enddo

! Output the volume table
*cfopen, volume_output65, txt ! Open a file to output the results
*do, step, 1, 40, 1
 *vwrite, step, VOLUME_TABLE(step)
 (F8.0, ', ', E15.6) ! Output format: first number is the step number, second number is the volume
*enddo
*cfclose

*dim, VOLUME_TABLE, array, 40 ! Create an array to store the volume for each load step, totaling 40 steps

/post1 ! Enter the post-processing module
*do, step, 1, 40, 1 ! Loop through each load step
 SET, step ! Set the current load step
 ALLSEL ! Select all elements and nodes

 ! Select elements with IDs in the range 1 to 15712
 ESEL, S, ELEM, , 1, 15712
 eplo ! Plot elements to visualize the selection

 *get, edtol, elem, 0, count ! Get the total number of selected elements (edtol)
 *get, edmin, elem, 0, num, min ! Get the minimum element ID (edmin)
 ETABLE, ETAB_STRESS, s, eqv ! Define equivalent stress (von Mises stress) as a variable in the stress table

 ! Initialize equivalent stress array
 ESTRESS =
 *dim, ESTRESS,, edtol ! Define the dimensions of the ESTRESS array as the total number of selected elements
 *do, ie, edmin, edtol, 1 ! Loop through all elements (from edmin to edtol)
 *if, ESEL(ie), eq, 1, then ! If the element is selected
 *GET, von, ELEM, ie, ETAB, ETAB_STRESS ! Get the equivalent stress of the current element
 *if, von, gt, 6.5, then ! If the equivalent stress is greater than 6.5
 *if, von, lt, 6.6, then ! And less than 6.6
 ESTRESS(ie) = 1 ! Set the corresponding position in the ESTRESS array to 1 (meets condition)
 *endif
 *endif
 *endif
 *enddo

 ESEL, S, ELEM, , 1, 15712 ! Ensure only elements with IDs in the range 1 to 15712 are selected
 eplo ! Plot elements to visualize the selection
 ESEL, NONE ! Clear all selections
 *do, ie, edmin, edtol, 1 ! Loop through all elements again
 *if, ESTRESS(ie), eq, 1, then ! If the value in the ESTRESS array is 1 (element meets condition)
 ESEL, A,,, ie, ! Add the element to the selection
 *endif
 *enddo
 cm, ee3, elem ! Create a component named "ee3" to store the selected elements

 ! Calculate the total volume
 cmsel, s, ee3, elem ! Select the component "ee3" with the selected elements
 etable, evol, volu ! Define the volume variable
 ssum ! Sum the selected elements to calculate the total volume
 *get, VOLUME_TABLE(step), ssum,, item, evol ! Store the total volume in the VOLUME_TABLE array at the current step position
*enddo

! Output the volume table
*cfopen, volume_output66, txt ! Open a file to output the results
*do, step, 1, 40, 1
 *vwrite, step, VOLUME_TABLE(step)
 (F8.0, ', ', E15.6) ! Output format: first number is the step number, second number is the volume
*enddo
*cfclose

*dim, VOLUME_TABLE, array, 40 ! Create an array to store the volume for each load step, totaling 40 steps

/post1 ! Enter the post-processing module
*do, step, 1, 40, 1 ! Loop through each load step
 SET, step ! Set the current load step
 ALLSEL ! Select all elements and nodes

 ! Select elements with IDs in the range 1 to 15712
 ESEL, S, ELEM, , 1, 15712
 eplo ! Plot elements to visualize the selection

 *get, edtol, elem, 0, count ! Get the total number of selected elements (edtol)
 *get, edmin, elem, 0, num, min ! Get the minimum element ID (edmin)
 ETABLE, ETAB_STRESS, s, eqv ! Define equivalent stress (von Mises stress) as a variable in the stress table

 ! Initialize equivalent stress array
 ESTRESS =
 *dim, ESTRESS,, edtol ! Define the dimensions of the ESTRESS array as the total number of selected elements
 *do, ie, edmin, edtol, 1 ! Loop through all elements (from edmin to edtol)
 *if, ESEL(ie), eq, 1, then ! If the element is selected
 *GET, von, ELEM, ie, ETAB, ETAB_STRESS ! Get the equivalent stress of the current element
 *if, von, gt, 6.6, then ! If the equivalent stress is greater than 6.6
 *if, von, lt, 6.7, then ! And less than 6.7
 ESTRESS(ie) = 1 ! Set the corresponding position in the ESTRESS array to 1 (meets condition)
 *endif
 *endif
 *endif
 *enddo

 ESEL, S, ELEM, , 1, 15712 ! Ensure only elements with IDs in the range 1 to 15712 are selected
 eplo ! Plot elements to visualize the selection
 ESEL, NONE ! Clear all selections
 *do, ie, edmin, edtol, 1 ! Loop through all elements again
 *if, ESTRESS(ie), eq, 1, then ! If the value in the ESTRESS array is 1 (element meets condition)
 ESEL, A,,, ie, ! Add the element to the selection
 *endif
 *enddo
 cm, ee3, elem ! Create a component named "ee3" to store the selected elements

 ! Calculate the total volume
 cmsel, s, ee3, elem ! Select the component "ee3" with the selected elements
 etable, evol, volu ! Define the volume variable
 ssum ! Sum the selected elements to calculate the total volume
 *get, VOLUME_TABLE(step), ssum,, item, evol ! Store the total volume in the VOLUME_TABLE array at the current step position
*enddo

! Output the volume table
*cfopen, volume_output67, txt ! Open a file to output the results
*do, step, 1, 40, 1
 *vwrite, step, VOLUME_TABLE(step)
 (F8.0, ', ', E15.6) ! Output format: first number is the step number, second number is the volume
*enddo
*cfclose

*dim, VOLUME_TABLE, array, 40 ! Create an array to store the volume for each load step, totaling 40 steps

/post1 ! Enter the post-processing module
*do, step, 1, 40, 1 ! Loop through each load step
 SET, step ! Set the current load step
 ALLSEL ! Select all elements and nodes

 ! Select elements with IDs in the range 1 to 15712
 ESEL, S, ELEM, , 1, 15712
 eplo ! Plot elements to visualize the selection

 *get, edtol, elem, 0, count ! Get the total number of selected elements (edtol)
 *get, edmin, elem, 0, num, min ! Get the minimum element ID (edmin)
 ETABLE, ETAB_STRESS, s, eqv ! Define equivalent stress (von Mises stress) as a variable in the stress table

 ! Initialize equivalent stress array
 ESTRESS =
 *dim, ESTRESS,, edtol ! Define the dimensions of the ESTRESS array as the total number of selected elements
 *do, ie, edmin, edtol, 1 ! Loop through all elements (from edmin to edtol)
 *if, ESEL(ie), eq, 1, then ! If the element is selected
 *GET, von, ELEM, ie, ETAB, ETAB_STRESS ! Get the equivalent stress of the current element
 *if, von, gt, 6.7, then ! If the equivalent stress is greater than 6.7
 *if, von, lt, 6.8, then ! And less than 6.8
 ESTRESS(ie) = 1 ! Set the corresponding position in the ESTRESS array to 1 (meets condition)
 *endif
 *endif
 *endif
 *enddo

 ESEL, S, ELEM, , 1, 15712 ! Ensure only elements with IDs in the range 1 to 15712 are selected
 eplo ! Plot elements to visualize the selection
 ESEL, NONE ! Clear all selections
 *do, ie, edmin, edtol, 1 ! Loop through all elements again
 *if, ESTRESS(ie), eq, 1, then ! If the value in the ESTRESS array is 1 (element meets condition)
 ESEL, A,,, ie, ! Add the element to the selection
 *endif
 *enddo
 cm, ee3, elem ! Create a component named "ee3" to store the selected elements

 ! Calculate the total volume
 cmsel, s, ee3, elem ! Select the component "ee3" with the selected elements
 etable, evol, volu ! Define the volume variable
 ssum ! Sum the selected elements to calculate the total volume
 *get, VOLUME_TABLE(step), ssum,, item, evol ! Store the total volume in the VOLUME_TABLE array at the current step position
*enddo

! Output the volume table
*cfopen, volume_output68, txt ! Open a file to output the results
*do, step, 1, 40, 1
 *vwrite, step, VOLUME_TABLE(step)
 (F8.0, ', ', E15.6) ! Output format: first number is the step number, second number is the volume
*enddo
*cfclose

*dim, VOLUME_TABLE, array, 40 ! Create an array to store the volume for each load step, totaling 40 steps

/post1 ! Enter the post-processing module
*do, step, 1, 40, 1 ! Loop through each load step
 SET, step ! Set the current load step
 ALLSEL ! Select all elements and nodes

 ! Select elements with IDs in the range 1 to 15712
 ESEL, S, ELEM, , 1, 15712
 eplo ! Plot elements to visualize the selection

 *get, edtol, elem, 0, count ! Get the total number of selected elements (edtol)
 *get, edmin, elem, 0, num, min ! Get the minimum element ID (edmin)
 ETABLE, ETAB_STRESS, s, eqv ! Define equivalent stress (von Mises stress) as a variable in the stress table

 ! Initialize equivalent stress array
 ESTRESS =
 *dim, ESTRESS,, edtol ! Define the dimensions of the ESTRESS array as the total number of selected elements
 *do, ie, edmin, edtol, 1 ! Loop through all elements (from edmin to edtol)
 *if, ESEL(ie), eq, 1, then ! If the element is selected
 *GET, von, ELEM, ie, ETAB, ETAB_STRESS ! Get the equivalent stress of the current element
 *if, von, gt, 6.8, then ! If the equivalent stress is greater than 6.8
 *if, von, lt, 6.9, then ! And less than 6.9
 ESTRESS(ie) = 1 ! Set the corresponding position in the ESTRESS array to 1 (meets condition)
 *endif
 *endif
 *endif
 *enddo

 ESEL, S, ELEM, , 1, 15712 ! Ensure only elements with IDs in the range 1 to 15712 are selected
 eplo ! Plot elements to visualize the selection
 ESEL, NONE ! Clear all selections
 *do, ie, edmin, edtol, 1 ! Loop through all elements again
 *if, ESTRESS(ie), eq, 1, then ! If the value in the ESTRESS array is 1 (element meets condition)
 ESEL, A,,, ie, ! Add the element to the selection
 *endif
 *enddo
 cm, ee3, elem ! Create a component named "ee3" to store the selected elements

 ! Calculate the total volume
 cmsel, s, ee3, elem ! Select the component "ee3" with the selected elements
 etable, evol, volu ! Define the volume variable
 ssum ! Sum the selected elements to calculate the total volume
 *get, VOLUME_TABLE(step), ssum,, item, evol ! Store the total volume in the VOLUME_TABLE array at the current step position
*enddo

! Output the volume table
*cfopen, volume_output69, txt ! Open a file to output the results
*do, step, 1, 40, 1
 *vwrite, step, VOLUME_TABLE(step)
 (F8.0, ', ', E15.6) ! Output format: first number is the step number, second number is the volume
*enddo
*cfclose

*dim, VOLUME_TABLE, array, 40 ! Create an array to store the volume for each load step, totaling 40 steps

/post1 ! Enter the post-processing module
*do, step, 1, 40, 1 ! Loop through each load step
 SET, step ! Set the current load step
 ALLSEL ! Select all elements and nodes

 ! Select elements with IDs in the range 1 to 15712
 ESEL, S, ELEM, , 1, 15712
 eplo ! Plot elements to visualize the selection

 *get, edtol, elem, 0, count ! Get the total number of selected elements (edtol)
 *get, edmin, elem, 0, num, min ! Get the minimum element ID (edmin)
 ETABLE, ETAB_STRESS, s, eqv ! Define equivalent stress (von Mises stress) as a variable in the stress table

 ! Initialize equivalent stress array
 ESTRESS =
 *dim, ESTRESS,, edtol ! Define the dimensions of the ESTRESS array as the total number of selected elements
 *do, ie, edmin, edtol, 1 ! Loop through all elements (from edmin to edtol)
 *if, ESEL(ie), eq, 1, then ! If the element is selected
 *GET, von, ELEM, ie, ETAB, ETAB_STRESS ! Get the equivalent stress of the current element
 *if, von, gt, 6.9, then ! If the equivalent stress is greater than 6.9
 *if, von, lt, 7.0, then ! And less than 7.0
 ESTRESS(ie) = 1 ! Set the corresponding position in the ESTRESS array to 1 (meets condition)
 *endif
 *endif
 *endif
 *enddo

 ESEL, S, ELEM, , 1, 15712 ! Ensure only elements with IDs in the range 1 to 15712 are selected
 eplo ! Plot elements to visualize the selection
 ESEL, NONE ! Clear all selections
 *do, ie, edmin, edtol, 1 ! Loop through all elements again
 *if, ESTRESS(ie), eq, 1, then ! If the value in the ESTRESS array is 1 (element meets condition)
 ESEL, A,,, ie, ! Add the element to the selection
 *endif
 *enddo
 cm, ee3, elem ! Create a component named "ee3" to store the selected elements

 ! Calculate the total volume
 cmsel, s, ee3, elem ! Select the component "ee3" with the selected elements
 etable, evol, volu ! Define the volume variable
 ssum ! Sum the selected elements to calculate the total volume
 *get, VOLUME_TABLE(step), ssum,, item, evol ! Store the total volume in the VOLUME_TABLE array at the current step position
*enddo

! Output the volume table
*cfopen, volume_output70, txt ! Open a file to output the results
*do, step, 1, 40, 1
 *vwrite, step, VOLUME_TABLE(step)
 (F8.0, ', ', E15.6) ! Output format: first number is the step number, second number is the volume
*enddo
*cfclose

*dim, VOLUME_TABLE, array, 40 ! Create an array to store the volume for each load step, totaling 40 steps

/post1 ! Enter the post-processing module
*do, step, 1, 40, 1 ! Loop through each load step
 SET, step ! Set the current load step
 ALLSEL ! Select all elements and nodes

 ! Select elements with IDs in the range 1 to 15712
 ESEL, S, ELEM, , 1, 15712
 eplo ! Plot elements to visualize the selection

 *get, edtol, elem, 0, count ! Get the total number of selected elements (edtol)
 *get, edmin, elem, 0, num, min ! Get the minimum element ID (edmin)
 ETABLE, ETAB_STRESS, s, eqv ! Define equivalent stress (von Mises stress) as a variable in the stress table

 ! Initialize equivalent stress array
 ESTRESS =
 *dim, ESTRESS,, edtol ! Define the dimensions of the ESTRESS array as the total number of selected elements
 *do, ie, edmin, edtol, 1 ! Loop through all elements (from edmin to edtol)
 *if, ESEL(ie), eq, 1, then ! If the element is selected
 *GET, von, ELEM, ie, ETAB, ETAB_STRESS ! Get the equivalent stress of the current element
 *if, von, gt, 7.0, then ! If the equivalent stress is greater than 7.0
 *if, von, lt, 7.1, then ! And less than 7.1
 ESTRESS(ie) = 1 ! Set the corresponding position in the ESTRESS array to 1 (meets condition)
 *endif
 *endif
 *endif
 *enddo

 ESEL, S, ELEM, , 1, 15712 ! Ensure only elements with IDs in the range 1 to 15712 are selected
 eplo ! Plot elements to visualize the selection
 ESEL, NONE ! Clear all selections
 *do, ie, edmin, edtol, 1 ! Loop through all elements again
 *if, ESTRESS(ie), eq, 1, then ! If the value in the ESTRESS array is 1 (element meets condition)
 ESEL, A,,, ie, ! Add the element to the selection
 *endif
 *enddo
 cm, ee3, elem ! Create a component named "ee3" to store the selected elements

 ! Calculate the total volume
 cmsel, s, ee3, elem ! Select the component "ee3" with the selected elements
 etable, evol, volu ! Define the volume variable
 ssum ! Sum the selected elements to calculate the total volume
 *get, VOLUME_TABLE(step), ssum,, item, evol ! Store the total volume in the VOLUME_TABLE array at the current step position
*enddo

! Output the volume table
*cfopen, volume_output71, txt ! Open a file to output the results
*do, step, 1, 40, 1
 *vwrite, step, VOLUME_TABLE(step)
 (F8.0, ', ', E15.6) ! Output format: first number is the step number, second number is the volume
*enddo
*cfclose

*dim, VOLUME_TABLE, array, 40 ! Create an array to store the volume for each load step, totaling 40 steps

/post1 ! Enter the post-processing module
*do, step, 1, 40, 1 ! Loop through each load step
 SET, step ! Set the current load step
 ALLSEL ! Select all elements and nodes

 ! Select elements with IDs in the range 1 to 15712
 ESEL, S, ELEM, , 1, 15712
 eplo ! Plot elements to visualize the selection

 *get, edtol, elem, 0, count ! Get the total number of selected elements (edtol)
 *get, edmin, elem, 0, num, min ! Get the minimum element ID (edmin)
 ETABLE, ETAB_STRESS, s, eqv ! Define equivalent stress (von Mises stress) as a variable in the stress table

 ! Initialize equivalent stress array
 ESTRESS =
 *dim, ESTRESS,, edtol ! Define the dimensions of the ESTRESS array as the total number of selected elements
 *do, ie, edmin, edtol, 1 ! Loop through all elements (from edmin to edtol)
 *if, ESEL(ie), eq, 1, then ! If the element is selected
 *GET, von, ELEM, ie, ETAB, ETAB_STRESS ! Get the equivalent stress of the current element
 *if, von, gt, 7.1, then ! If the equivalent stress is greater than 7.1
 *if, von, lt, 7.2, then ! And less than 7.2
 ESTRESS(ie) = 1 ! Set the corresponding position in the ESTRESS array to 1 (meets condition)
 *endif
 *endif
 *endif
 *enddo

 ESEL, S, ELEM, , 1, 15712 ! Ensure only elements with IDs in the range 1 to 15712 are selected
 eplo ! Plot elements to visualize the selection
 ESEL, NONE ! Clear all selections
 *do, ie, edmin, edtol, 1 ! Loop through all elements again
 *if, ESTRESS(ie), eq, 1, then ! If the value in the ESTRESS array is 1 (element meets condition)
 ESEL, A,,, ie, ! Add the element to the selection
 *endif
 *enddo
 cm, ee3, elem ! Create a component named "ee3" to store the selected elements

 ! Calculate the total volume
 cmsel, s, ee3, elem ! Select the component "ee3" with the selected elements
 etable, evol, volu ! Define the volume variable
 ssum ! Sum the selected elements to calculate the total volume
 *get, VOLUME_TABLE(step), ssum,, item, evol ! Store the total volume in the VOLUME_TABLE array at the current step position
*enddo

! Output the volume table
*cfopen, volume_output72, txt ! Open a file to output the results
*do, step, 1, 40, 1
 *vwrite, step, VOLUME_TABLE(step)
 (F8.0, ', ', E15.6) ! Output format: first number is the step number, second number is the volume
*enddo
*cfclose

*dim, VOLUME_TABLE, array, 40 ! Create an array to store the volume for each load step, totaling 40 steps

/post1 ! Enter the post-processing module
*do, step, 1, 40, 1 ! Loop through each load step
 SET, step ! Set the current load step
 ALLSEL ! Select all elements and nodes

 ! Select elements with IDs in the range 1 to 15712
 ESEL, S, ELEM, , 1, 15712
 eplo ! Plot elements to visualize the selection

 *get, edtol, elem, 0, count ! Get the total number of selected elements (edtol)
 *get, edmin, elem, 0, num, min ! Get the minimum element ID (edmin)
 ETABLE, ETAB_STRESS, s, eqv ! Define equivalent stress (von Mises stress) as a variable in the stress table

 ! Initialize equivalent stress array
 ESTRESS =
 *dim, ESTRESS,, edtol ! Define the dimensions of the ESTRESS array as the total number of selected elements
 *do, ie, edmin, edtol, 1 ! Loop through all elements (from edmin to edtol)
 *if, ESEL(ie), eq, 1, then ! If the element is selected
 *GET, von, ELEM, ie, ETAB, ETAB_STRESS ! Get the equivalent stress of the current element
 *if, von, gt, 7.2, then ! If the equivalent stress is greater than 7.2
 *if, von, lt, 7.3, then ! And less than 7.3
 ESTRESS(ie) = 1 ! Set the corresponding position in the ESTRESS array to 1 (meets condition)
 *endif
 *endif
 *endif
 *enddo

 ESEL, S, ELEM, , 1, 15712 ! Ensure only elements with IDs in the range 1 to 15712 are selected
 eplo ! Plot elements to visualize the selection
 ESEL, NONE ! Clear all selections
 *do, ie, edmin, edtol, 1 ! Loop through all elements again
 *if, ESTRESS(ie), eq, 1, then ! If the value in the ESTRESS array is 1 (element meets condition)
 ESEL, A,,, ie, ! Add the element to the selection
 *endif
 *enddo
 cm, ee3, elem ! Create a component named "ee3" to store the selected elements

 ! Calculate the total volume
 cmsel, s, ee3, elem ! Select the component "ee3" with the selected elements
 etable, evol, volu ! Define the volume variable
 ssum ! Sum the selected elements to calculate the total volume
 *get, VOLUME_TABLE(step), ssum,, item, evol ! Store the total volume in the VOLUME_TABLE array at the current step position
*enddo

! Output the volume table
*cfopen, volume_output73, txt ! Open a file to output the results
*do, step, 1, 40, 1
 *vwrite, step, VOLUME_TABLE(step)
 (F8.0, ', ', E15.6) ! Output format: first number is the step number, second number is the volume
*enddo
*cfclose

*dim, VOLUME_TABLE, array, 40 ! Create an array to store the volume for each load step, totaling 40 steps

/post1 ! Enter the post-processing module
*do, step, 1, 40, 1 ! Loop through each load step
 SET, step ! Set the current load step
 ALLSEL ! Select all elements and nodes

 ! Select elements with IDs in the range 1 to 15712
 ESEL, S, ELEM, , 1, 15712
 eplo ! Plot elements to visualize the selection

 *get, edtol, elem, 0, count ! Get the total number of selected elements (edtol)
 *get, edmin, elem, 0, num, min ! Get the minimum element ID (edmin)
 ETABLE, ETAB_STRESS, s, eqv ! Define equivalent stress (von Mises stress) as a variable in the stress table

 ! Initialize equivalent stress array
 ESTRESS =
 *dim, ESTRESS,, edtol ! Define the dimensions of the ESTRESS array as the total number of selected elements
 *do, ie, edmin, edtol, 1 ! Loop through all elements (from edmin to edtol)
 *if, ESEL(ie), eq, 1, then ! If the element is selected
 *GET, von, ELEM, ie, ETAB, ETAB_STRESS ! Get the equivalent stress of the current element
 *if, von, gt, 7.3, then ! If the equivalent stress is greater than 7.3
 *if, von, lt, 7.4, then ! And less than 7.4
 ESTRESS(ie) = 1 ! Set the corresponding position in the ESTRESS array to 1 (meets condition)
 *endif
 *endif
 *endif
 *enddo

 ESEL, S, ELEM, , 1, 15712 ! Ensure only elements with IDs in the range 1 to 15712 are selected
 eplo ! Plot elements to visualize the selection
 ESEL, NONE ! Clear all selections
 *do, ie, edmin, edtol, 1 ! Loop through all elements again
 *if, ESTRESS(ie), eq, 1, then ! If the value in the ESTRESS array is 1 (element meets condition)
 ESEL, A,,, ie, ! Add the element to the selection
 *endif
 *enddo
 cm, ee3, elem ! Create a component named "ee3" to store the selected elements

 ! Calculate the total volume
 cmsel, s, ee3, elem ! Select the component "ee3" with the selected elements
 etable, evol, volu ! Define the volume variable
 ssum ! Sum the selected elements to calculate the total volume
 *get, VOLUME_TABLE(step), ssum,, item, evol ! Store the total volume in the VOLUME_TABLE array at the current step position
*enddo

! Output the volume table
*cfopen, volume_output74, txt ! Open a file to output the results
*do, step, 1, 40, 1
 *vwrite, step, VOLUME_TABLE(step)
 (F8.0, ', ', E15.6) ! Output format: first number is the step number, second number is the volume
*enddo
*cfclose

*dim, VOLUME_TABLE, array, 40 ! Create an array to store the volume for each load step, totaling 40 steps

/post1 ! Enter the post-processing module
*do, step, 1, 40, 1 ! Loop through each load step
 SET, step ! Set the current load step
 ALLSEL ! Select all elements and nodes

 ! Select elements with IDs in the range 1 to 15712
 ESEL, S, ELEM, , 1, 15712
 eplo ! Plot elements to visualize the selection

 *get, edtol, elem, 0, count ! Get the total number of selected elements (edtol)
 *get, edmin, elem, 0, num, min ! Get the minimum element ID (edmin)
 ETABLE, ETAB_STRESS, s, eqv ! Define equivalent stress (von Mises stress) as a variable in the stress table

 ! Initialize equivalent stress array
 ESTRESS =
 *dim, ESTRESS,, edtol ! Define the dimensions of the ESTRESS array as the total number of selected elements
 *do, ie, edmin, edtol, 1 ! Loop through all elements (from edmin to edtol)
 *if, ESEL(ie), eq, 1, then ! If the element is selected
 *GET, von, ELEM, ie, ETAB, ETAB_STRESS ! Get the equivalent stress of the current element
 *if, von, gt, 7.4, then ! If the equivalent stress is greater than 7.4
 *if, von, lt, 7.5, then ! And less than 7.5
 ESTRESS(ie) = 1 ! Set the corresponding position in the ESTRESS array to 1 (meets condition)
 *endif
 *endif
 *endif
 *enddo

 ESEL, S, ELEM, , 1, 15712 ! Ensure only elements with IDs in the range 1 to 15712 are selected
 eplo ! Plot elements to visualize the selection
 ESEL, NONE ! Clear all selections
 *do, ie, edmin, edtol, 1 ! Loop through all elements again
 *if, ESTRESS(ie), eq, 1, then ! If the value in the ESTRESS array is 1 (element meets condition)
 ESEL, A,,, ie, ! Add the element to the selection
 *endif
 *enddo
 cm, ee3, elem ! Create a component named "ee3" to store the selected elements

 ! Calculate the total volume
 cmsel, s, ee3, elem ! Select the component "ee3" with the selected elements
 etable, evol, volu ! Define the volume variable
 ssum ! Sum the selected elements to calculate the total volume
 *get, VOLUME_TABLE(step), ssum,, item, evol ! Store the total volume in the VOLUME_TABLE array at the current step position
*enddo

! Output the volume table
*cfopen, volume_output75, txt ! Open a file to output the results
*do, step, 1, 40, 1
 *vwrite, step, VOLUME_TABLE(step)
 (F8.0, ', ', E15.6) ! Output format: first number is the step number, second number is the volume
*enddo
*cfclose

*dim, VOLUME_TABLE, array, 40 ! Create an array to store the volume for each load step, totaling 40 steps

/post1 ! Enter the post-processing module
*do, step, 1, 40, 1 ! Loop through each load step
 SET, step ! Set the current load step
 ALLSEL ! Select all elements and nodes

 ! Select elements with IDs in the range 1 to 15712
 ESEL, S, ELEM, , 1, 15712
 eplo ! Plot elements to visualize the selection

 *get, edtol, elem, 0, count ! Get the total number of selected elements (edtol)
 *get, edmin, elem, 0, num, min ! Get the minimum element ID (edmin)
 ETABLE, ETAB_STRESS, s, eqv ! Define equivalent stress (von Mises stress) as a variable in the stress table

 ! Initialize equivalent stress array
 ESTRESS =
 *dim, ESTRESS,, edtol ! Define the dimensions of the ESTRESS array as the total number of selected elements
 *do, ie, edmin, edtol, 1 ! Loop through all elements (from edmin to edtol)
 *if, ESEL(ie), eq, 1, then ! If the element is selected
 *GET, von, ELEM, ie, ETAB, ETAB_STRESS ! Get the equivalent stress of the current element
 *if, von, gt, 7.5, then ! If the equivalent stress is greater than 7.5
 *if, von, lt, 7.6, then ! And less than 7.6
 ESTRESS(ie) = 1 ! Set the corresponding position in the ESTRESS array to 1 (meets condition)
 *endif
 *endif
 *endif
 *enddo

 ESEL, S, ELEM, , 1, 15712 ! Ensure only elements with IDs in the range 1 to 15712 are selected
 eplo ! Plot elements to visualize the selection
 ESEL, NONE ! Clear all selections
 *do, ie, edmin, edtol, 1 ! Loop through all elements again
 *if, ESTRESS(ie), eq, 1, then ! If the value in the ESTRESS array is 1 (element meets condition)
 ESEL, A,,, ie, ! Add the element to the selection
 *endif
 *enddo
 cm, ee3, elem ! Create a component named "ee3" to store the selected elements

 ! Calculate the total volume
 cmsel, s, ee3, elem ! Select the component "ee3" with the selected elements
 etable, evol, volu ! Define the volume variable
 ssum ! Sum the selected elements to calculate the total volume
 *get, VOLUME_TABLE(step), ssum,, item, evol ! Store the total volume in the VOLUME_TABLE array at the current step position
*enddo

! Output the volume table
*cfopen, volume_output76, txt ! Open a file to output the results
*do, step, 1, 40, 1
 *vwrite, step, VOLUME_TABLE(step)
 (F8.0, ', ', E15.6) ! Output format: first number is the step number, second number is the volume
*enddo
*cfclose

*dim, VOLUME_TABLE, array, 40 ! Create an array to store the volume for each load step, totaling 40 steps

/post1 ! Enter the post-processing module
*do, step, 1, 40, 1 ! Loop through each load step
 SET, step ! Set the current load step
 ALLSEL ! Select all elements and nodes

 ! Select elements with IDs in the range 1 to 15712
 ESEL, S, ELEM, , 1, 15712
 eplo ! Plot elements to visualize the selection

 *get, edtol, elem, 0, count ! Get the total number of selected elements (edtol)
 *get, edmin, elem, 0, num, min ! Get the minimum element ID (edmin)
 ETABLE, ETAB_STRESS, s, eqv ! Define equivalent stress (von Mises stress) as a variable in the stress table

 ! Initialize equivalent stress array
 ESTRESS =
 *dim, ESTRESS,, edtol ! Define the dimensions of the ESTRESS array as the total number of selected elements
 *do, ie, edmin, edtol, 1 ! Loop through all elements (from edmin to edtol)
 *if, ESEL(ie), eq, 1, then ! If the element is selected
 *GET, von, ELEM, ie, ETAB, ETAB_STRESS ! Get the equivalent stress of the current element
 *if, von, gt, 7.6, then ! If the equivalent stress is greater than 7.6
 *if, von, lt, 7.7, then ! And less than 7.7
 ESTRESS(ie) = 1 ! Set the corresponding position in the ESTRESS array to 1 (meets condition)
 *endif
 *endif
 *endif
 *enddo

 ESEL, S, ELEM, , 1, 15712 ! Ensure only elements with IDs in the range 1 to 15712 are selected
 eplo ! Plot elements to visualize the selection
 ESEL, NONE ! Clear all selections
 *do, ie, edmin, edtol, 1 ! Loop through all elements again
 *if, ESTRESS(ie), eq, 1, then ! If the value in the ESTRESS array is 1 (element meets condition)
 ESEL, A,,, ie, ! Add the element to the selection
 *endif
 *enddo
 cm, ee3, elem ! Create a component named "ee3" to store the selected elements

 ! Calculate the total volume
 cmsel, s, ee3, elem ! Select the component "ee3" with the selected elements
 etable, evol, volu ! Define the volume variable
 ssum ! Sum the selected elements to calculate the total volume
 *get, VOLUME_TABLE(step), ssum,, item, evol ! Store the total volume in the VOLUME_TABLE array at the current step position
*enddo

! Output the volume table
*cfopen, volume_output77, txt ! Open a file to output the results
*do, step, 1, 40, 1
 *vwrite, step, VOLUME_TABLE(step)
 (F8.0, ', ', E15.6) ! Output format: first number is the step number, second number is the volume
*enddo
*cfclose

*dim, VOLUME_TABLE, array, 40 ! Create an array to store the volume for each load step, totaling 40 steps

/post1 ! Enter the post-processing module
*do, step, 1, 40, 1 ! Loop through each load step
 SET, step ! Set the current load step
 ALLSEL ! Select all elements and nodes

 ! Select elements with IDs in the range 1 to 15712
 ESEL, S, ELEM, , 1, 15712
 eplo ! Plot elements to visualize the selection

 *get, edtol, elem, 0, count ! Get the total number of selected elements (edtol)
 *get, edmin, elem, 0, num, min ! Get the minimum element ID (edmin)
 ETABLE, ETAB_STRESS, s, eqv ! Define equivalent stress (von Mises stress) as a variable in the stress table

 ! Initialize equivalent stress array
 ESTRESS =
 *dim, ESTRESS,, edtol ! Define the dimensions of the ESTRESS array as the total number of selected elements
 *do, ie, edmin, edtol, 1 ! Loop through all elements (from edmin to edtol)
 *if, ESEL(ie), eq, 1, then ! If the element is selected
 *GET, von, ELEM, ie, ETAB, ETAB_STRESS ! Get the equivalent stress of the current element
 *if, von, gt, 7.7, then ! If the equivalent stress is greater than 7.7
 *if, von, lt, 7.8, then ! And less than 7.8
 ESTRESS(ie) = 1 ! Set the corresponding position in the ESTRESS array to 1 (meets condition)
 *endif
 *endif
 *endif
 *enddo

 ESEL, S, ELEM, , 1, 15712 ! Ensure only elements with IDs in the range 1 to 15712 are selected
 eplo ! Plot elements to visualize the selection
 ESEL, NONE ! Clear all selections
 *do, ie, edmin, edtol, 1 ! Loop through all elements again
 *if, ESTRESS(ie), eq, 1, then ! If the value in the ESTRESS array is 1 (element meets condition)
 ESEL, A,,, ie, ! Add the element to the selection
 *endif
 *enddo
 cm, ee3, elem ! Create a component named "ee3" to store the selected elements

 ! Calculate the total volume
 cmsel, s, ee3, elem ! Select the component "ee3" with the selected elements
 etable, evol, volu ! Define the volume variable
 ssum ! Sum the selected elements to calculate the total volume
 *get, VOLUME_TABLE(step), ssum,, item, evol ! Store the total volume in the VOLUME_TABLE array at the current step position
*enddo

! Output the volume table
*cfopen, volume_output78, txt ! Open a file to output the results
*do, step, 1, 40, 1
 *vwrite, step, VOLUME_TABLE(step)
 (F8.0, ', ', E15.6) ! Output format: first number is the step number, second number is the volume
*enddo
*cfclose

*dim, VOLUME_TABLE, array, 40 ! Create an array to store the volume for each load step, totaling 40 steps

/post1 ! Enter the post-processing module
*do, step, 1, 40, 1 ! Loop through each load step
 SET, step ! Set the current load step
 ALLSEL ! Select all elements and nodes

 ! Select elements with IDs in the range 1 to 15712
 ESEL, S, ELEM, , 1, 15712
 eplo ! Plot elements to visualize the selection

 *get, edtol, elem, 0, count ! Get the total number of selected elements (edtol)
 *get, edmin, elem, 0, num, min ! Get the minimum element ID (edmin)
 ETABLE, ETAB_STRESS, s, eqv ! Define equivalent stress (von Mises stress) as a variable in the stress table

 ! Initialize equivalent stress array
 ESTRESS =
 *dim, ESTRESS,, edtol ! Define the dimensions of the ESTRESS array as the total number of selected elements
 *do, ie, edmin, edtol, 1 ! Loop through all elements (from edmin to edtol)
 *if, ESEL(ie), eq, 1, then ! If the element is selected
 *GET, von, ELEM, ie, ETAB, ETAB_STRESS ! Get the equivalent stress of the current element
 *if, von, gt, 7.8, then ! If the equivalent stress is greater than 7.8
 *if, von, lt, 7.9, then ! And less than 7.9
 ESTRESS(ie) = 1 ! Set the corresponding position in the ESTRESS array to 1 (meets condition)
 *endif
 *endif
 *endif
 *enddo

 ESEL, S, ELEM, , 1, 15712 ! Ensure only elements with IDs in the range 1 to 15712 are selected
 eplo ! Plot elements to visualize the selection
 ESEL, NONE ! Clear all selections
 *do, ie, edmin, edtol, 1 ! Loop through all elements again
 *if, ESTRESS(ie), eq, 1, then ! If the value in the ESTRESS array is 1 (element meets condition)
 ESEL, A,,, ie, ! Add the element to the selection
 *endif
 *enddo
 cm, ee3, elem ! Create a component named "ee3" to store the selected elements

 ! Calculate the total volume
 cmsel, s, ee3, elem ! Select the component "ee3" with the selected elements
 etable, evol, volu ! Define the volume variable
 ssum ! Sum the selected elements to calculate the total volume
 *get, VOLUME_TABLE(step), ssum,, item, evol ! Store the total volume in the VOLUME_TABLE array at the current step position
*enddo

! Output the volume table
*cfopen, volume_output79, txt ! Open a file to output the results
*do, step, 1, 40, 1
 *vwrite, step, VOLUME_TABLE(step)
 (F8.0, ', ', E15.6) ! Output format: first number is the step number, second number is the volume
*enddo
*cfclose

*dim, VOLUME_TABLE, array, 40 ! Create an array to store the volume for each load step, totaling 40 steps

/post1 ! Enter the post-processing module
*do, step, 1, 40, 1 ! Loop through each load step
 SET, step ! Set the current load step
 ALLSEL ! Select all elements and nodes

 ! Select elements with IDs in the range 1 to 15712
 ESEL, S, ELEM, , 1, 15712
 eplo ! Plot elements to visualize the selection

 *get, edtol, elem, 0, count ! Get the total number of selected elements (edtol)
 *get, edmin, elem, 0, num, min ! Get the minimum element ID (edmin)
 ETABLE, ETAB_STRESS, s, eqv ! Define equivalent stress (von Mises stress) as a variable in the stress table

 ! Initialize equivalent stress array
 ESTRESS =
 *dim, ESTRESS,, edtol ! Define the dimensions of the ESTRESS array as the total number of selected elements
 *do, ie, edmin, edtol, 1 ! Loop through all elements (from edmin to edtol)
 *if, ESEL(ie), eq, 1, then ! If the element is selected
 *GET, von, ELEM, ie, ETAB, ETAB_STRESS ! Get the equivalent stress of the current element
 *if, von, gt, 7.9, then ! If the equivalent stress is greater than 7.9
 *if, von, lt, 8.0, then ! And less than 8.0
 ESTRESS(ie) = 1 ! Set the corresponding position in the ESTRESS array to 1 (meets condition)
 *endif
 *endif
 *endif
 *enddo

 ESEL, S, ELEM, , 1, 15712 ! Ensure only elements with IDs in the range 1 to 15712 are selected
 eplo ! Plot elements to visualize the selection
 ESEL, NONE ! Clear all selections
 *do, ie, edmin, edtol, 1 ! Loop through all elements again
 *if, ESTRESS(ie), eq, 1, then ! If the value in the ESTRESS array is 1 (element meets condition)
 ESEL, A,,, ie, ! Add the element to the selection
 *endif
 *enddo
 cm, ee3, elem ! Create a component named "ee3" to store the selected elements

 ! Calculate the total volume
 cmsel, s, ee3, elem ! Select the component "ee3" with the selected elements
 etable, evol, volu ! Define the volume variable
 ssum ! Sum the selected elements to calculate the total volume
 *get, VOLUME_TABLE(step), ssum,, item, evol ! Store the total volume in the VOLUME_TABLE array at the current step position
*enddo

! Output the volume table
*cfopen, volume_output80, txt ! Open a file to output the results
*do, step, 1, 40, 1
 *vwrite, step, VOLUME_TABLE(step)
 (F8.0, ', ', E15.6) ! Output format: first number is the step number, second number is the volume
*enddo
*cfclose

*dim, VOLUME_TABLE, array, 40 ! Create an array to store the volume for each load step, totaling 40 steps

/post1 ! Enter the post-processing module
*do, step, 1, 40, 1 ! Loop through each load step
 SET, step ! Set the current load step
 ALLSEL ! Select all elements and nodes

 ! Select elements with IDs in the range 1 to 15712
 ESEL, S, ELEM, , 1, 15712
 eplo ! Plot elements to visualize the selection

 *get, edtol, elem, 0, count ! Get the total number of selected elements (edtol)
 *get, edmin, elem, 0, num, min ! Get the minimum element ID (edmin)
 ETABLE, ETAB_STRESS, s, eqv ! Define equivalent stress (von Mises stress) as a variable in the stress table

 ! Initialize equivalent stress array
 ESTRESS =
 *dim, ESTRESS,, edtol ! Define the dimensions of the ESTRESS array as the total number of selected elements
 *do, ie, edmin, edtol, 1 ! Loop through all elements (from edmin to edtol)
 *if, ESEL(ie), eq, 1, then ! If the element is selected
 *GET, von, ELEM, ie, ETAB, ETAB_STRESS ! Get the equivalent stress of the current element
 *if, von, gt, 8.0, then ! If the equivalent stress is greater than 8.0
 *if, von, lt, 8.1, then ! And less than 8.1
 ESTRESS(ie) = 1 ! Set the corresponding position in the ESTRESS array to 1 (meets condition)
 *endif
 *endif
 *endif
 *enddo

 ESEL, S, ELEM, , 1, 15712 ! Ensure only elements with IDs in the range 1 to 15712 are selected
 eplo ! Plot elements to visualize the selection
 ESEL, NONE ! Clear all selections
 *do, ie, edmin, edtol, 1 ! Loop through all elements again
 *if, ESTRESS(ie), eq, 1, then ! If the value in the ESTRESS array is 1 (element meets condition)
 ESEL, A,,, ie, ! Add the element to the selection
 *endif
 *enddo
 cm, ee3, elem ! Create a component named "ee3" to store the selected elements

 ! Calculate the total volume
 cmsel, s, ee3, elem ! Select the component "ee3" with the selected elements
 etable, evol, volu ! Define the volume variable
 ssum ! Sum the selected elements to calculate the total volume
 *get, VOLUME_TABLE(step), ssum,, item, evol ! Store the total volume in the VOLUME_TABLE array at the current step position
*enddo

! Output the volume table
*cfopen, volume_output81, txt ! Open a file to output the results
*do, step, 1, 40, 1
 *vwrite, step, VOLUME_TABLE(step)
 (F8.0, ', ', E15.6) ! Output format: first number is the step number, second number is the volume
*enddo
*cfclose

*dim, VOLUME_TABLE, array, 40 ! Create an array to store the volume for each load step, totaling 40 steps

/post1 ! Enter the post-processing module
*do, step, 1, 40, 1 ! Loop through each load step
 SET, step ! Set the current load step
 ALLSEL ! Select all elements and nodes

 ! Select elements with IDs in the range 1 to 15712
 ESEL, S, ELEM, , 1, 15712
 eplo ! Plot elements to visualize the selection

 *get, edtol, elem, 0, count ! Get the total number of selected elements (edtol)
 *get, edmin, elem, 0, num, min ! Get the minimum element ID (edmin)
 ETABLE, ETAB_STRESS, s, eqv ! Define equivalent stress (von Mises stress) as a variable in the stress table

 ! Initialize equivalent stress array
 ESTRESS =
 *dim, ESTRESS,, edtol ! Define the dimensions of the ESTRESS array as the total number of selected elements
 *do, ie, edmin, edtol, 1 ! Loop through all elements (from edmin to edtol)
 *if, ESEL(ie), eq, 1, then ! If the element is selected
 *GET, von, ELEM, ie, ETAB, ETAB_STRESS ! Get the equivalent stress of the current element
 *if, von, gt, 8.1, then ! If the equivalent stress is greater than 8.1
 *if, von, lt, 8.2, then ! And less than 8.2
 ESTRESS(ie) = 1 ! Set the corresponding position in the ESTRESS array to 1 (meets condition)
 *endif
 *endif
 *endif
 *enddo

 ESEL, S, ELEM, , 1, 15712 ! Ensure only elements with IDs in the range 1 to 15712 are selected
 eplo ! Plot elements to visualize the selection
 ESEL, NONE ! Clear all selections
 *do, ie, edmin, edtol, 1 ! Loop through all elements again
 *if, ESTRESS(ie), eq, 1, then ! If the value in the ESTRESS array is 1 (element meets condition)
 ESEL, A,,, ie, ! Add the element to the selection
 *endif
 *enddo
 cm, ee3, elem ! Create a component named "ee3" to store the selected elements

 ! Calculate the total volume
 cmsel, s, ee3, elem ! Select the component "ee3" with the selected elements
 etable, evol, volu ! Define the volume variable
 ssum ! Sum the selected elements to calculate the total volume
 *get, VOLUME_TABLE(step), ssum,, item, evol ! Store the total volume in the VOLUME_TABLE array at the current step position
*enddo

! Output the volume table
*cfopen, volume_output82, txt ! Open a file to output the results
*do, step, 1, 40, 1
 *vwrite, step, VOLUME_TABLE(step)
 (F8.0, ', ', E15.6) ! Output format: first number is the step number, second number is the volume
*enddo
*cfclose

*dim, VOLUME_TABLE, array, 40 ! Create an array to store the volume for each load step, totaling 40 steps

/post1 ! Enter the post-processing module
*do, step, 1, 40, 1 ! Loop through each load step
 SET, step ! Set the current load step
 ALLSEL ! Select all elements and nodes

 ! Select elements with IDs in the range 1 to 15712
 ESEL, S, ELEM, , 1, 15712
 eplo ! Plot elements to visualize the selection

 *get, edtol, elem, 0, count ! Get the total number of selected elements (edtol)
 *get, edmin, elem, 0, num, min ! Get the minimum element ID (edmin)
 ETABLE, ETAB_STRESS, s, eqv ! Define equivalent stress (von Mises stress) as a variable in the stress table

 ! Initialize equivalent stress array
 ESTRESS =
 *dim, ESTRESS,, edtol ! Define the dimensions of the ESTRESS array as the total number of selected elements
 *do, ie, edmin, edtol, 1 ! Loop through all elements (from edmin to edtol)
 *if, ESEL(ie), eq, 1, then ! If the element is selected
 *GET, von, ELEM, ie, ETAB, ETAB_STRESS ! Get the equivalent stress of the current element
 *if, von, gt, 8.2, then ! If the equivalent stress is greater than 8.2
 *if, von, lt, 8.3, then ! And less than 8.3
 ESTRESS(ie) = 1 ! Set the corresponding position in the ESTRESS array to 1 (meets condition)
 *endif
 *endif
 *endif
 *enddo

 ESEL, S, ELEM, , 1, 15712 ! Ensure only elements with IDs in the range 1 to 15712 are selected
 eplo ! Plot elements to visualize the selection
 ESEL, NONE ! Clear all selections
 *do, ie, edmin, edtol, 1 ! Loop through all elements again
 *if, ESTRESS(ie), eq, 1, then ! If the value in the ESTRESS array is 1 (element meets condition)
 ESEL, A,,, ie, ! Add the element to the selection
 *endif
 *enddo
 cm, ee3, elem ! Create a component named "ee3" to store the selected elements

 ! Calculate the total volume
 cmsel, s, ee3, elem ! Select the component "ee3" with the selected elements
 etable, evol, volu ! Define the volume variable
 ssum ! Sum the selected elements to calculate the total volume
 *get, VOLUME_TABLE(step), ssum,, item, evol ! Store the total volume in the VOLUME_TABLE array at the current step position
*enddo

! Output the volume table
*cfopen, volume_output83, txt ! Open a file to output the results
*do, step, 1, 40, 1
 *vwrite, step, VOLUME_TABLE(step)
 (F8.0, ', ', E15.6) ! Output format: first number is the step number, second number is the volume
*enddo
*cfclose

*dim, VOLUME_TABLE, array, 40 ! Create an array to store the volume for each load step, totaling 40 steps

/post1 ! Enter the post-processing module
*do, step, 1, 40, 1 ! Loop through each load step
 SET, step ! Set the current load step
 ALLSEL ! Select all elements and nodes

 ! Select elements with IDs in the range 1 to 15712
 ESEL, S, ELEM, , 1, 15712
 eplo ! Plot elements to visualize the selection

 *get, edtol, elem, 0, count ! Get the total number of selected elements (edtol)
 *get, edmin, elem, 0, num, min ! Get the minimum element ID (edmin)
 ETABLE, ETAB_STRESS, s, eqv ! Define equivalent stress (von Mises stress) as a variable in the stress table

 ! Initialize equivalent stress array
 ESTRESS =
 *dim, ESTRESS,, edtol ! Define the dimensions of the ESTRESS array as the total number of selected elements
 *do, ie, edmin, edtol, 1 ! Loop through all elements (from edmin to edtol)
 *if, ESEL(ie), eq, 1, then ! If the element is selected
 *GET, von, ELEM, ie, ETAB, ETAB_STRESS ! Get the equivalent stress of the current element
 *if, von, gt, 8.3, then ! If the equivalent stress is greater than 8.3
 *if, von, lt, 8.4, then ! And less than 8.4
 ESTRESS(ie) = 1 ! Set the corresponding position in the ESTRESS array to 1 (meets condition)
 *endif
 *endif
 *endif
 *enddo

 ESEL, S, ELEM, , 1, 15712 ! Ensure only elements with IDs in the range 1 to 15712 are selected
 eplo ! Plot elements to visualize the selection
 ESEL, NONE ! Clear all selections
 *do, ie, edmin, edtol, 1 ! Loop through all elements again
 *if, ESTRESS(ie), eq, 1, then ! If the value in the ESTRESS array is 1 (element meets condition)
 ESEL, A,,, ie, ! Add the element to the selection
 *endif
 *enddo
 cm, ee3, elem ! Create a component named "ee3" to store the selected elements

 ! Calculate the total volume
 cmsel, s, ee3, elem ! Select the component "ee3" with the selected elements
 etable, evol, volu ! Define the volume variable
 ssum ! Sum the selected elements to calculate the total volume
 *get, VOLUME_TABLE(step), ssum,, item, evol ! Store the total volume in the VOLUME_TABLE array at the current step position
*enddo

! Output the volume table
*cfopen, volume_output84, txt ! Open a file to output the results
*do, step, 1, 40, 1
 *vwrite, step, VOLUME_TABLE(step)
 (F8.0, ', ', E15.6) ! Output format: first number is the step number, second number is the volume
*enddo
*cfclose

*dim, VOLUME_TABLE, array, 40 ! Create an array to store the volume for each load step, totaling 40 steps

/post1 ! Enter the post-processing module
*do, step, 1, 40, 1 ! Loop through each load step
 SET, step ! Set the current load step
 ALLSEL ! Select all elements and nodes

 ! Select elements with IDs in the range 1 to 15712
 ESEL, S, ELEM, , 1, 15712
 eplo ! Plot elements to visualize the selection

 *get, edtol, elem, 0, count ! Get the total number of selected elements (edtol)
 *get, edmin, elem, 0, num, min ! Get the minimum element ID (edmin)
 ETABLE, ETAB_STRESS, s, eqv ! Define equivalent stress (von Mises stress) as a variable in the stress table

 ! Initialize equivalent stress array
 ESTRESS =
 *dim, ESTRESS,, edtol ! Define the dimensions of the ESTRESS array as the total number of selected elements
 *do, ie, edmin, edtol, 1 ! Loop through all elements (from edmin to edtol)
 *if, ESEL(ie), eq, 1, then ! If the element is selected
 *GET, von, ELEM, ie, ETAB, ETAB_STRESS ! Get the equivalent stress of the current element
 *if, von, gt, 8.4, then ! If the equivalent stress is greater than 8.4
 *if, von, lt, 8.5, then ! And less than 8.5
 ESTRESS(ie) = 1 ! Set the corresponding position in the ESTRESS array to 1 (meets condition)
 *endif
 *endif
 *endif
 *enddo

 ESEL, S, ELEM, , 1, 15712 ! Ensure only elements with IDs in the range 1 to 15712 are selected
 eplo ! Plot elements to visualize the selection
 ESEL, NONE ! Clear all selections
 *do, ie, edmin, edtol, 1 ! Loop through all elements again
 *if, ESTRESS(ie), eq, 1, then ! If the value in the ESTRESS array is 1 (element meets condition)
 ESEL, A,,, ie, ! Add the element to the selection
 *endif
 *enddo
 cm, ee3, elem ! Create a component named "ee3" to store the selected elements

 ! Calculate the total volume
 cmsel, s, ee3, elem ! Select the component "ee3" with the selected elements
 etable, evol, volu ! Define the volume variable
 ssum ! Sum the selected elements to calculate the total volume
 *get, VOLUME_TABLE(step), ssum,, item, evol ! Store the total volume in the VOLUME_TABLE array at the current step position
*enddo

! Output the volume table
*cfopen, volume_output85, txt ! Open a file to output the results
*do, step, 1, 40, 1
 *vwrite, step, VOLUME_TABLE(step)
 (F8.0, ', ', E15.6) ! Output format: first number is the step number, second number is the volume
*enddo
*cfclose

*dim, VOLUME_TABLE, array, 40 ! Create an array to store the volume for each load step, totaling 40 steps

/post1 ! Enter the post-processing module
*do, step, 1, 40, 1 ! Loop through each load step
 SET, step ! Set the current load step
 ALLSEL ! Select all elements and nodes

 ! Select elements with IDs in the range 1 to 15712
 ESEL, S, ELEM, , 1, 15712
 eplo ! Plot elements to visualize the selection

 *get, edtol, elem, 0, count ! Get the total number of selected elements (edtol)
 *get, edmin, elem, 0, num, min ! Get the minimum element ID (edmin)
 ETABLE, ETAB_STRESS, s, eqv ! Define equivalent stress (von Mises stress) as a variable in the stress table

 ! Initialize equivalent stress array
 ESTRESS =
 *dim, ESTRESS,, edtol ! Define the dimensions of the ESTRESS array as the total number of selected elements
 *do, ie, edmin, edtol, 1 ! Loop through all elements (from edmin to edtol)
 *if, ESEL(ie), eq, 1, then ! If the element is selected
 *GET, von, ELEM, ie, ETAB, ETAB_STRESS ! Get the equivalent stress of the current element
 *if, von, gt, 8.5, then ! If the equivalent stress is greater than 8.5
 *if, von, lt, 8.6, then ! And less than 8.6
 ESTRESS(ie) = 1 ! Set the corresponding position in the ESTRESS array to 1 (meets condition)
 *endif
 *endif
 *endif
 *enddo

 ESEL, S, ELEM, , 1, 15712 ! Ensure only elements with IDs in the range 1 to 15712 are selected
 eplo ! Plot elements to visualize the selection
 ESEL, NONE ! Clear all selections
 *do, ie, edmin, edtol, 1 ! Loop through all elements again
 *if, ESTRESS(ie), eq, 1, then ! If the value in the ESTRESS array is 1 (element meets condition)
 ESEL, A,,, ie, ! Add the element to the selection
 *endif
 *enddo
 cm, ee3, elem ! Create a component named "ee3" to store the selected elements

 ! Calculate the total volume
 cmsel, s, ee3, elem ! Select the component "ee3" with the selected elements
 etable, evol, volu ! Define the volume variable
 ssum ! Sum the selected elements to calculate the total volume
 *get, VOLUME_TABLE(step), ssum,, item, evol ! Store the total volume in the VOLUME_TABLE array at the current step position
*enddo

! Output the volume table
*cfopen, volume_output86, txt ! Open a file to output the results
*do, step, 1, 40, 1
 *vwrite, step, VOLUME_TABLE(step)
 (F8.0, ', ', E15.6) ! Output format: first number is the step number, second number is the volume
*enddo
*cfclose

*dim, VOLUME_TABLE, array, 40 ! Create an array to store the volume for each load step, totaling 40 steps

/post1 ! Enter the post-processing module
*do, step, 1, 40, 1 ! Loop through each load step
 SET, step ! Set the current load step
 ALLSEL ! Select all elements and nodes

 ! Select elements with IDs in the range 1 to 15712
 ESEL, S, ELEM, , 1, 15712
 eplo ! Plot elements to visualize the selection

 *get, edtol, elem, 0, count ! Get the total number of selected elements (edtol)
 *get, edmin, elem, 0, num, min ! Get the minimum element ID (edmin)
 ETABLE, ETAB_STRESS, s, eqv ! Define equivalent stress (von Mises stress) as a variable in the stress table

 ! Initialize equivalent stress array
 ESTRESS =
 *dim, ESTRESS,, edtol ! Define the dimensions of the ESTRESS array as the total number of selected elements
 *do, ie, edmin, edtol, 1 ! Loop through all elements (from edmin to edtol)
 *if, ESEL(ie), eq, 1, then ! If the element is selected
 *GET, von, ELEM, ie, ETAB, ETAB_STRESS ! Get the equivalent stress of the current element
 *if, von, gt, 8.6, then ! If the equivalent stress is greater than 8.6
 *if, von, lt, 8.7, then ! And less than 8.7
 ESTRESS(ie) = 1 ! Set the corresponding position in the ESTRESS array to 1 (meets condition)
 *endif
 *endif
 *endif
 *enddo

 ESEL, S, ELEM, , 1, 15712 ! Ensure only elements with IDs in the range 1 to 15712 are selected
 eplo ! Plot elements to visualize the selection
 ESEL, NONE ! Clear all selections
 *do, ie, edmin, edtol, 1 ! Loop through all elements again
 *if, ESTRESS(ie), eq, 1, then ! If the value in the ESTRESS array is 1 (element meets condition)
 ESEL, A,,, ie, ! Add the element to the selection
 *endif
 *enddo
 cm, ee3, elem ! Create a component named "ee3" to store the selected elements

 ! Calculate the total volume
 cmsel, s, ee3, elem ! Select the component "ee3" with the selected elements
 etable, evol, volu ! Define the volume variable
 ssum ! Sum the selected elements to calculate the total volume
 *get, VOLUME_TABLE(step), ssum,, item, evol ! Store the total volume in the VOLUME_TABLE array at the current step position
*enddo

! Output the volume table
*cfopen, volume_output87, txt ! Open a file to output the results
*do, step, 1, 40, 1
 *vwrite, step, VOLUME_TABLE(step)
 (F8.0, ', ', E15.6) ! Output format: first number is the step number, second number is the volume
*enddo
*cfclose

*dim, VOLUME_TABLE, array, 40 ! Create an array to store the volume for each load step, totaling 40 steps

/post1 ! Enter the post-processing module
*do, step, 1, 40, 1 ! Loop through each load step
 SET, step ! Set the current load step
 ALLSEL ! Select all elements and nodes

 ! Select elements with IDs in the range 1 to 15712
 ESEL, S, ELEM, , 1, 15712
 eplo ! Plot elements to visualize the selection

 *get, edtol, elem, 0, count ! Get the total number of selected elements (edtol)
 *get, edmin, elem, 0, num, min ! Get the minimum element ID (edmin)
 ETABLE, ETAB_STRESS, s, eqv ! Define equivalent stress (von Mises stress) as a variable in the stress table

 ! Initialize equivalent stress array
 ESTRESS =
 *dim, ESTRESS,, edtol ! Define the dimensions of the ESTRESS array as the total number of selected elements
 *do, ie, edmin, edtol, 1 ! Loop through all elements (from edmin to edtol)
 *if, ESEL(ie), eq, 1, then ! If the element is selected
 *GET, von, ELEM, ie, ETAB, ETAB_STRESS ! Get the equivalent stress of the current element
 *if, von, gt, 8.7, then ! If the equivalent stress is greater than 8.7
 *if, von, lt, 8.8, then ! And less than 8.8
 ESTRESS(ie) = 1 ! Set the corresponding position in the ESTRESS array to 1 (meets condition)
 *endif
 *endif
 *endif
 *enddo

 ESEL, S, ELEM, , 1, 15712 ! Ensure only elements with IDs in the range 1 to 15712 are selected
 eplo ! Plot elements to visualize the selection
 ESEL, NONE ! Clear all selections
 *do, ie, edmin, edtol, 1 ! Loop through all elements again
 *if, ESTRESS(ie), eq, 1, then ! If the value in the ESTRESS array is 1 (element meets condition)
 ESEL, A,,, ie, ! Add the element to the selection
 *endif
 *enddo
 cm, ee3, elem ! Create a component named "ee3" to store the selected elements

 ! Calculate the total volume
 cmsel, s, ee3, elem ! Select the component "ee3" with the selected elements
 etable, evol, volu ! Define the volume variable
 ssum ! Sum the selected elements to calculate the total volume
 *get, VOLUME_TABLE(step), ssum,, item, evol ! Store the total volume in the VOLUME_TABLE array at the current step position
*enddo

! Output the volume table
*cfopen, volume_output88, txt ! Open a file to output the results
*do, step, 1, 40, 1
 *vwrite, step, VOLUME_TABLE(step)
 (F8.0, ', ', E15.6) ! Output format: first number is the step number, second number is the volume
*enddo
*cfclose

*dim, VOLUME_TABLE, array, 40 ! Create an array to store the volume for each load step, totaling 40 steps

/post1 ! Enter the post-processing module
*do, step, 1, 40, 1 ! Loop through each load step
 SET, step ! Set the current load step
 ALLSEL ! Select all elements and nodes

 ! Select elements with IDs in the range 1 to 15712
 ESEL, S, ELEM, , 1, 15712
 eplo ! Plot elements to visualize the selection

 *get, edtol, elem, 0, count ! Get the total number of selected elements (edtol)
 *get, edmin, elem, 0, num, min ! Get the minimum element ID (edmin)
 ETABLE, ETAB_STRESS, s, eqv ! Define equivalent stress (von Mises stress) as a variable in the stress table

 ! Initialize equivalent stress array
 ESTRESS =
 *dim, ESTRESS,, edtol ! Define the dimensions of the ESTRESS array as the total number of selected elements
 *do, ie, edmin, edtol, 1 ! Loop through all elements (from edmin to edtol)
 *if, ESEL(ie), eq, 1, then ! If the element is selected
 *GET, von, ELEM, ie, ETAB, ETAB_STRESS ! Get the equivalent stress of the current element
 *if, von, gt, 8.8, then ! If the equivalent stress is greater than 8.8
 *if, von, lt, 8.9, then ! And less than 8.9
 ESTRESS(ie) = 1 ! Set the corresponding position in the ESTRESS array to 1 (meets condition)
 *endif
 *endif
 *endif
 *enddo

 ESEL, S, ELEM, , 1, 15712 ! Ensure only elements with IDs in the range 1 to 15712 are selected
 eplo ! Plot elements to visualize the selection
 ESEL, NONE ! Clear all selections
 *do, ie, edmin, edtol, 1 ! Loop through all elements again
 *if, ESTRESS(ie), eq, 1, then ! If the value in the ESTRESS array is 1 (element meets condition)
 ESEL, A,,, ie, ! Add the element to the selection
 *endif
 *enddo
 cm, ee3, elem ! Create a component named "ee3" to store the selected elements

 ! Calculate the total volume
 cmsel, s, ee3, elem ! Select the component "ee3" with the selected elements
 etable, evol, volu ! Define the volume variable
 ssum ! Sum the selected elements to calculate the total volume
 *get, VOLUME_TABLE(step), ssum,, item, evol ! Store the total volume in the VOLUME_TABLE array at the current step position
*enddo

! Output the volume table
*cfopen, volume_output89, txt ! Open a file to output the results
*do, step, 1, 40, 1
 *vwrite, step, VOLUME_TABLE(step)
 (F8.0, ', ', E15.6) ! Output format: first number is the step number, second number is the volume
*enddo
*cfclose

*dim, VOLUME_TABLE, array, 40 ! Create an array to store the volume for each load step, totaling 40 steps

/post1 ! Enter the post-processing module
*do, step, 1, 40, 1 ! Loop through each load step
 SET, step ! Set the current load step
 ALLSEL ! Select all elements and nodes

 ! Select elements with IDs in the range 1 to 15712
 ESEL, S, ELEM, , 1, 15712
 eplo ! Plot elements to visualize the selection

 *get, edtol, elem, 0, count ! Get the total number of selected elements (edtol)
 *get, edmin, elem, 0, num, min ! Get the minimum element ID (edmin)
 ETABLE, ETAB_STRESS, s, eqv ! Define equivalent stress (von Mises stress) as a variable in the stress table

 ! Initialize equivalent stress array
 ESTRESS =
 *dim, ESTRESS,, edtol ! Define the dimensions of the ESTRESS array as the total number of selected elements
 *do, ie, edmin, edtol, 1 ! Loop through all elements (from edmin to edtol)
 *if, ESEL(ie), eq, 1, then ! If the element is selected
 *GET, von, ELEM, ie, ETAB, ETAB_STRESS ! Get the equivalent stress of the current element
 *if, von, gt, 8.9, then ! If the equivalent stress is greater than 8.9
 *if, von, lt, 9.0, then ! And less than 9.0
 ESTRESS(ie) = 1 ! Set the corresponding position in the ESTRESS array to 1 (meets condition)
 *endif
 *endif
 *endif
 *enddo

 ESEL, S, ELEM, , 1, 15712 ! Ensure only elements with IDs in the range 1 to 15712 are selected
 eplo ! Plot elements to visualize the selection
 ESEL, NONE ! Clear all selections
 *do, ie, edmin, edtol, 1 ! Loop through all elements again
 *if, ESTRESS(ie), eq, 1, then ! If the value in the ESTRESS array is 1 (element meets condition)
 ESEL, A,,, ie, ! Add the element to the selection
 *endif
 *enddo
 cm, ee3, elem ! Create a component named "ee3" to store the selected elements

 ! Calculate the total volume
 cmsel, s, ee3, elem ! Select the component "ee3" with the selected elements
 etable, evol, volu ! Define the volume variable
 ssum ! Sum the selected elements to calculate the total volume
 *get, VOLUME_TABLE(step), ssum,, item, evol ! Store the total volume in the VOLUME_TABLE array at the current step position
*enddo

! Output the volume table
*cfopen, volume_output90, txt ! Open a file to output the results
*do, step, 1, 40, 1
 *vwrite, step, VOLUME_TABLE(step)
 (F8.0, ', ', E15.6) ! Output format: first number is the step number, second number is the volume
*enddo
*cfclose
